# Barriers and Delays in Tuberculosis Diagnosis and Treatment Services: Does Gender Matter?

**DOI:** 10.1155/2014/461935

**Published:** 2014-04-28

**Authors:** Wei-Teng Yang, Celine R. Gounder, Tokunbo Akande, Jan-Walter De Neve, Katherine N. McIntire, Aditya Chandrasekhar, Alan de Lima Pereira, Naveen Gummadi, Santanu Samanta, Amita Gupta

**Affiliations:** ^1^Johns Hopkins Bloomberg School of Public Health, Baltimore, MD 21205, USA; ^2^Department of Medicine, Johns Hopkins University School of Medicine, Baltimore, MD 21287, USA; ^3^Harvard School of Public Health, Boston, MA 02115, USA; ^4^Narayana Hrudayalaya Hospital, Hyderabad 500055, India; ^5^All India Institute of Medical Sciences, New Delhi 110029, India; ^6^Center for Clinical Global Health Education, 600 North Wolfe Street, Phipps 540B, Baltimore, MD 21287, USA

## Abstract

*Background*. Tuberculosis (TB) remains a global public health problem with known gender-related disparities. We reviewed the quantitative evidence for gender-related differences in accessing TB services from symptom onset to treatment initiation. *Methods*. Following a systematic review process, we: searched 12 electronic databases; included quantitative studies assessing gender differences in accessing TB diagnostic and treatment services; abstracted data; and assessed study validity. We defined barriers and delays at the individual and provider/system levels using a conceptual framework of the TB care continuum and examined gender-related differences. *Results*. Among 13,448 articles, 137 were included: many assessed individual-level barriers (52%) and delays (42%), 76% surveyed persons presenting for care with diagnosed or suspected TB, 24% surveyed community members, and two-thirds were from African and Asian regions. Many studies reported no gender differences. Among studies reporting disparities, women faced greater barriers (financial: 64% versus 36%; physical: 100% versus 0%; stigma: 85% versus 15%; health literacy: 67% versus 33%; and provider-/system-level: 100% versus 0%) and longer delays (presentation to diagnosis: 45% versus 0%) than men. *Conclusions*. Many studies found no quantitative gender-related differences in barriers and delays limiting access to TB services. When differences were identified, women experienced greater barriers and longer delays than men.

## 1. Introduction

Tuberculosis (TB) remains a significant global public health issue. Significantly, the TB disease burden is unequally distributed among men and women. Of the estimated 8.7 million incident TB cases and 1.4 million deaths caused by TB globally in 2011, roughly one-third occurred among women (2.9 million incident TB cases and 0.5 million deaths) [1]. Currently, it is unclear whether these disparities are due to sex-related differences (i.e., biology), gender-based differences (i.e., sociocultural practices and different social roles of men and women), or both [2–4]. Until recently, gender-related differences in the epidemiology, diagnosis, treatment, outcomes, and socioeconomic costs of TB have received relatively little attention. To address this knowledge gap, the World Health Organization (WHO) has proposed a framework and priorities for research on gender and TB [5].

To date, gender-based research supports that men and women respond differently to illness and face different barriers when accessing TB diagnostic and treatment services [2]. Barriers that limit access to TB services occur at the individual and provider/system levels. Individual-level barriers involve physical (distance to TB services and access to transport), financial (the direct and indirect costs of seeking TB services), stigma (stigma surrounding TB and its association with HIV), health literacy (TB-related knowledge and education), and sociocultural (gender roles and status in the family) factors, whereas provider/system-level barriers include provider degree of suspicion for TB, the number and types of providers seen before TB diagnosis, provider adherence to national TB program guidelines, and patient satisfaction with TB services. A comprehensive understanding of gender-related differences in barriers and delays at each level is needed so that researchers and policymakers can formulate and prioritize gender-specific interventions to improve the global impact of TB services.

Although several reviews have examined gender-related barriers and delays in seeking TB care [2, 3, 6–11], none have simultaneously assessed the contribution of both barriers and delays in a systematic manner. Furthermore, previous reviews have assessed a narrow study population. Currently, no review has captured the full continuum of TB care by including studies that have surveyed the general population, high-risk populations (e.g., homeless or HIV-infected persons), TB suspects who may not have sought care (e.g., untreated individuals with chest symptoms in the community), and TB patients and suspects presenting for care.

Our review aims to address these limitations. Using a partially-adopted, published framework [5], we systematically reviewed the literature to examine the quantitative evidence for gender-related differences in the barriers and delays that limit access to TB services along the continuum of care from symptom onset to treatment initiation. In this report, we present the findings from our quantitative review, which have important implications for TB service programs, research, and policymakers alike.

## 2. Methods

### 2.1. Systematic Review Process

#### 2.1.1. Search Strategy

We searched 12 electronic databases for human and English articles published between January 1953 and October 2010. We developed our search strategy for MEDLINE using PubMed with a combination of controlled vocabulary and keyword terms and phrases (see Supplementary Material available online at http://dx.doi.org/10.1155/2014/461935). The strategy was then translated for the Excerpta Medica Database (EMBASE), the Cumulative Index to Nursing and Allied Health Literature (CINAHL), Global Health, Popline, Africa Wide, LILACS, Web of Science, and the inclusive databases of the Cochrane Library using their respective thesaurus terms, synonyms, and keywords. Citations from each database were imported into a reference management system, and duplicates were removed.

#### 2.1.2. Study Selection Criteria

We included quantitative studies that reported on gender-related differences in barriers to and/or delays in accessing TB diagnostic and treatment services and studied human participants aged 15 years or older. Studies that did not provide a gender comparison as well as case reports, editorials, review articles, commentaries, practice guidelines, and studies of treatment compliance and/or outcomes were excluded. Participants were defined as persons with diagnosed or suspected TB, persons from either the general population or high-risk populations (e.g., HIV-infected, homeless, and prisoner), or health care providers. Diagnosed TB included both pulmonary and extrapulmonary forms, and TB diagnosis could be made by sputum smear microscopy, culture, or chest X-ray using histopathological or clinical criteria.

#### 2.1.3. Study Selection Process

**Figure 1:**
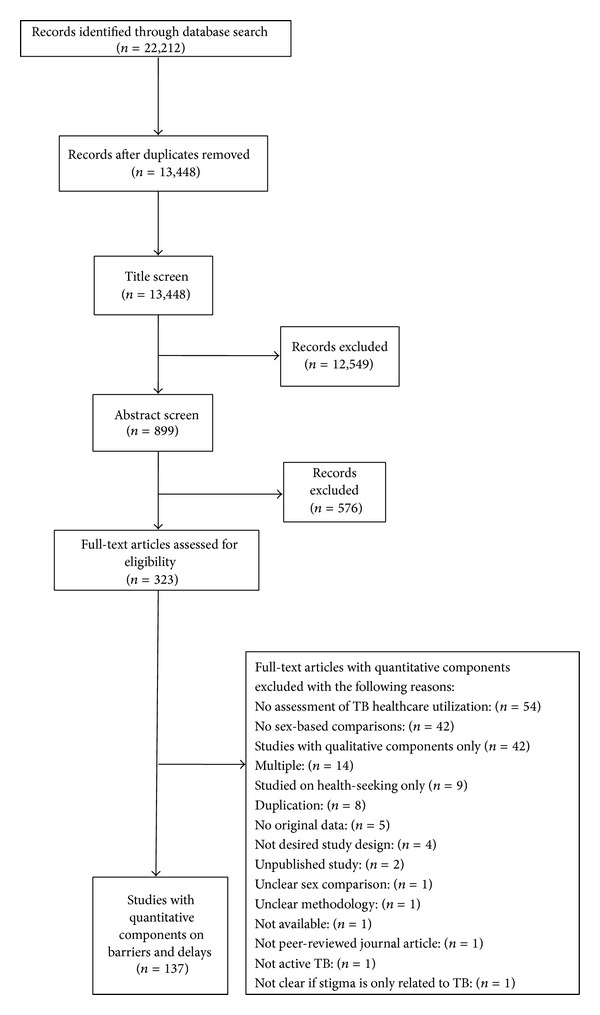
Study selection process.

Following deduplication, studies were reviewed sequentially by title, abstract, and in full-text form (Figure 1). At each stage, two reviewers independently evaluated each study against study selection criteria. Articles were included or excluded only when both reviewers were in agreement, and conflicts were resolved by a third, independent reviewer (AC, AG, or CRG). To ensure sufficient concordance between reviewers, a pilot review and reviewer discussion were conducted at each stage before proceeding with the remaining studies. Six reviewers conducted the title screen (ADP, JWDN, NG, SS, TA, and WTY), and four reviewers conducted the abstract screen and the full-text screen (ADP, JWDN, TA, and WTY). Following the full-text screen, included articles underwent the full-text assessment, which included data abstraction and a study validity assessment.

#### 2.1.4. Data Abstraction

Four reviewers (ADP, JWDN, TA, and WTY) independently abstracted quantitative data from each included full-text article in duplicate, and any conflicts were resolved through discussion with a third, independent reviewer (AG or CRG). Abstracted summary measures included differences in means or proportions, risk ratios, odds ratios, and hazards ratios.

#### 2.1.5. Validity Assessment

We used validity assessment tools to examine the quality of studies that inform our review; the assessment was not used to exclude studies. We assessed observational studies using items adopted from the methods and results sections of the Strengthening the Reporting of Observational Studies in Epidemiology (STROBE) checklist [148]. We used items adopted from the Consolidated Standards of Reporting Trials (CONSORT) checklist extension for clustered randomized trials to assess an included clustered randomized trial [149] and a pragmatic randomized controlled trial [150]. Two reviewers independently assessed the validity of each study using the adopted items (TA and WTY), and conflicts were resolved through discussion and arbitration with a third reviewer (CRG).

### 2.2. Outcomes and Definitions

**Figure 2:**
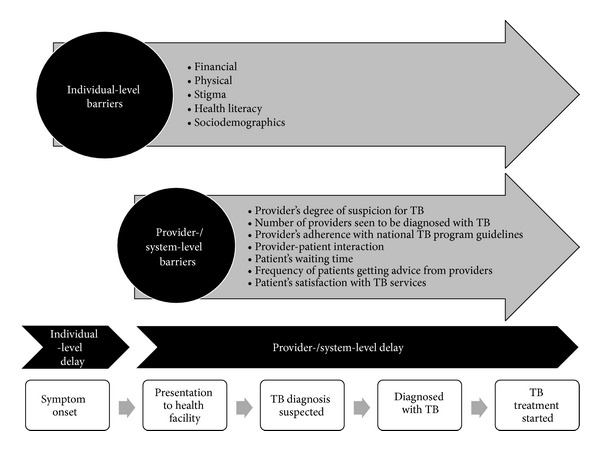
Conceptual framework illustrating barriers and delays that limit access to TB diagnostic and treatment services. The figure illustrates the conceptual framework of the tuberculosis (TB) care continuum from symptom onset to treatment initiation that we used to define barriers and delays that limit access to TB diagnostic and treatment services at the individual and provider/system levels. Individual-level barriers impact access to TB services along the full continuum of TB care, and provider-/system-level barriers impact access to TB services from patient presentation to any health care provider through TB treatment initiation. Barriers may contribute to delays between each step along the TB care continuum. Accordingly, we define individual-level delay as the delay between symptom onset and presentation to any health care provider; provider/system delay as the delay between presentation to any health care provider and diagnosis, the delay between presentation to any health care provider and treatment initiation or the delay between diagnosis and treatment initiation; and combined individual/provider/system delay as the delay between symptom onset and diagnosis or the delay between symptom onset and treatment initiation.

Outcomes were quantitative associations between gender and both barriers and delays that limit access to TB services along the full continuum of TB care from symptom onset through diagnosis and treatment initiation.  Figure 2 presents the conceptual framework that we used to define barriers and delays at the individual and provider/system levels at various time points along the continuum of TB care. Individual-level barriers were defined to be financial (the direct or indirect costs of TB care, including costs of travel, diagnosis, and/or treatment as well as the opportunity costs of lost employment, compensation, or household work); physical (distance, travel logistics, and/or access to TB care facilities); stigma (TB-specific sociocultural barriers arising from community or individual prejudice related to TB diagnosis or treatment, including social isolation, marriage prospects, fertility concerns, and association with HIV); health literacy (TB-related knowledge and education); and sociodemographic (age, race, rural versus urban residence, social caste, norms of practice, and social hierarchies). Provider-/system-level barriers were defined as any of the following: provider degree of suspicion for TB, number of providers seen before TB diagnosis, provider adherence to national TB program guidelines, provider-patient interaction, patient waiting time, frequency of getting advice, and patient satisfaction with TB services. Delay was defined as any time period between points along the TB care pathway under our conceptual framework from symptom onset to TB treatment initiation (Figure 2). Although barriers and delays are highly interrelated, few studies assess the contribution of barriers to delays quantitatively. Therefore, we present results for barriers and delays separately. We presented the impact of certain barriers on delays whenever possible.

## 3. Results

### 3.1. Study Characteristics

Our search strategy yielded 13,448 citations. Of these, 323 articles were reviewed in full-text form, and 137 studies met our selection criteria and were included in our review (Figure 1). Among the included studies, there was one (<1%) cluster-randomized clinical trial [91], one (<1%) pragmatic randomized controlled trial [55], eight (6%) cohort studies [33, 37, 67, 68, 87, 92, 136, 137], one (<1%) case-control study [69], and 126 (92%) cross-sectional studies [12–32, 34–36, 38–54, 56–66, 70–86, 88–90, 93–135, 138–147, 151]. Most studies (76%) assessed persons presenting for care with diagnosed or suspected TB, and the median sample size was 335 (IQR 190–1000) with women comprising less than half of the study population (median, interquartile range [IQR]: 42%, 34–49%). Most studies were published between 2000 and 2010, and two-thirds were conducted in Africa and Asia (Table 1).

### 3.2. Outcomes

Overall, the included studies reported on gender-related barriers and delays at the individual, provider/system, and combined individual/provider/system levels. Specifically, 71 (52%) studies assessed individual-level barriers, 19 (14%) studies assessed provider-/system-level barriers, and 7 (5%) studies assessed combined individual-/provider-/system-level barriers. Individual-level delays were assessed by 58 (42%) studies, 37 (27%) studies assessed provider/system-level delays, and 25 (18%) studies assessed combined individual-/provider-/system-level delays. Key findings are summarized below by outcome type (barrier or delay) and level of impact (individual, provider/system, combined individual/provider/system) (Table 2 and Supplementary Table S1).

### 3.3. Individual-Level Barriers

#### 3.3.1. Financial

Of 137 studies, 21 (15%) examined gender-related financial barriers to accessing TB services. Overall, a large number of studies found that women faced more financial barriers to seeking TB service than men. Fewer studies found either no difference in financial barriers between men and women or men faced greater financial barriers to accessing care (e.g., the opportunity cost of lost wages or income). While both men and women reported financial barriers to seeking TB services, the nature of these barriers differed. Women were more likely to be financially dependent on others [19, 26], unemployed, or without income [16, 17, 20]. Women also experienced greater healthcare seeking costs due to transport or the need for an escort [12, 17, 31], which may impact a woman's autonomy in seeking care. One study found that women may have also experienced greater financial barriers than men because they were more likely to see private providers than public providers [18]. The total direct costs of seeking TB diagnostic services as a proportion of income were higher for women than men in urban Zambia, largely because women had lower monthly incomes than men [13]. In Malawi, the indirect household costs of seeking care were higher for women [15].

#### 3.3.2. Physical

Of 137 studies, only nine (7%) explored gender-related physical barriers to accessing TB services. All nine studies found that distance and travel time to a health facility were similar for men and women. However, one study noted that distance to a clinic was more likely to result in delayed diagnosis among women than men [14].

#### 3.3.3. Stigma

Of 137 studies, 18% investigated gender-related differences in TB-related stigma as a barrier to accessing TB diagnostic and treatment services. Of these, 12 found no gender-related differences in stigma, 11 found that women reported greater TB-related stigma than men, and two studies found that men experienced greater TB-related stigma than women. Only two studies specifically examined the impact of TB-related stigma on gender-based differences in individual-level delays in seeking TB services; one study found that the impact of stigma on delay was greater among women than men [47], and the other study found no gender-based difference [48]. Four studies examined the impact of TB-related stigma on marriage and marital prospects, and all reported that women were more likely than men to believe that TB would have an adverse impact on marriage prospects and marriage [35, 39, 43, 44].

#### 3.3.4. Health Literacy

Of 137 studies, 36% described gender-related differences in TB-related knowledge and education as barriers to accessing TB services, and the majority of these (80%) examined differences in knowledge of the etiology, transmission, symptoms, diagnosis, and/or treatment of TB.

Of the 39 studies that assessed TB-related health literacy, 18 found that men and women had similar levels of TB-related knowledge, and, among those, six were conducted strictly in urban settings, and five were conducted in both urban and rural settings. Fourteen studies found that men had higher levels of TB-related knowledge than women; nine of these were conducted in strictly rural settings, and four were conducted in both rural and urban settings. Seven studies found that women had higher levels of TB-related knowledge than men; only one of these was conducted in a strictly rural setting. In addition, among ten studies that examined general educational attainment and literacy as barriers to accessing TB services, seven found that men were more educated and/or had higher literacy rates than women, and the remaining three studies found no gender-related differences.

Only two studies looked at the impact of TB-related knowledge and education on individual-level delays in presenting to TB services; one found that women suffered longer delays than men due to poor TB-related knowledge and education [14], and one found no gender-related differences [59]. One intervention trial found that, compared to women who did not receive brief instruction before submitting sputum samples, women who received instruction yielded significantly increased rates of both sputum positivity and return for submission of a second sputum sample. However, no significant changes were found among men who received such instruction [55]. This suggests that the intervention removed poor knowledge as a barrier for women to provide good sputum samples and to return for second sputum submission. Among two studies that examined the impact of TB-related knowledge on the likelihood of seeking tertiary-level care, one found that TB-related knowledge was more predictive of seeking hospital care among men than among women [41], and one found no gender-related difference [61].

#### 3.3.5. Sociodemographic

Only six (4%) studies explored gender-related differences in sociodemographic barriers (factors of older age, family size, marital status, or caste) to accessing TB services. Older women were more likely than older men to either delay or not seek care [79–81]. Compared to men, lower caste was more likely to predict individual-level delays among women [80], but family size had no gender-related differential impact on delays in seeking care [36]. Two studies explored the impact of being unmarried, separated, divorced, or widowed on seeking TB care [17, 71]. Among TB patients in Kenya, there was no gender-related difference in the impact of marital status on seeking care for TB [71]. However, in Bangladesh, women were more likely to be adversely affected than men [17].

### 3.4. Provider-/System-Level Barriers

Of 137 studies, 19 (14%) assessed gender-related barriers to accessing TB services at the provider and system levels. Overall, these studies were highly heterogeneous both in the barriers that were assessed and the findings.

Barriers to accessing diagnostic and/or treatment services at the provider and system levels were examined by nine (47%) studies. Of these, eight studies examined gender-related barriers to TB diagnosis and screening. In Thailand, it was found that providers were more likely to adhere to TB diagnostic guidelines among males with suspected TB compared to females with suspected TB [83]. In Malawi, males and females with suspected TB made a similar number of visits to a health facility before being diagnosed with TB [15, 90], and, in India, males and females with suspected TB were offered sputum smear microscopy with similar frequency [89]. In contrast, women in Gambia sought care from a larger number of healthcare providers to obtain a TB diagnosis than men [86], and, in Vietnam, women took more health-seeking actions for their symptoms than men but were offered sputum smear examinations significantly less often [21]. Among patients hospitalized and diagnosed with TB in the United States, women faced greater provider-/system-level delays in undergoing sputum smear microscopy than men [85]. However, among HIV-infected patients in the United States, men and women were screened for TB with similar frequency [87]. Only one study assessed gender-related barriers to TB treatment following a diagnosis of TB and found no differences between male and female patients with respect to provider-related factors [28].

Gender-related differences in patient satisfaction with TB services were examined by seven (37%) studies [17, 34, 35, 37, 52, 73, 84]. In Nepal and Egypt, males and females with suspected TB had similar levels of satisfaction with TB services [34, 35]. However, women in Egypt were less satisfied with drug availability than men, and women in Bangladesh and Syria were less satisfied with TB clinic hours, providers, and services than men, all of which were also predictors of health-seeking [17, 35, 37]. Compared to men, a greater proportion of women in Tanzania reported that a good provider-patient relationship was an important factor in their satisfaction with TB services [73]. Vietnamese TB patients reported no gender-related differences in the health education they received about their disease [52]. In another Tanzanian study where patients were randomized to community-based versus clinic-based TB treatment, male patients were more satisfied with community-based treatment than female patients [84]. Divided opinion regarding venue of treatment was noted in the study. Some patients preferred community-based treatment due to convenience, reduced transport costs, saved time, and reduced lost wages, whereas others preferred clinic-based treatment because it led to greater access to other clinical services and health education [84].

The remaining three studies reported on gender-related differences in health literacy among providers and TB-related hospitalization. Two studies assessed gender-based differences in TB-related knowledge among health workers and found no gender-based differences among providers in Oman and Iraq where patients may be more likely to seek care from providers of the same sex [75, 88]. One study in Tajikistan found that male TB patients were more likely to be hospitalized for treatment than female TB patients; other predictors of hospitalization in this study included positive sputum smear and availability of hospital beds [82].

### 3.5. Combined Individual-/Provider-/System-Level Barriers

Seven (5%) studies assessed gender-related differences in TB case detection rates, which were impacted by combined individual-/provider-/system-level barriers. Community-based active case finding was one strategy used to overcome combined level barriers to accessing TB diagnostic services [152, 153]. Seven studies compared community-based active case finding versus passive case finding (i.e., self-referral). Of these, five found that community-based active case finding increased TB case detection rates more significantly among women than men [29, 91–94]; one found greater increases in case detection rates among men than women [95]; and one found no difference in the change of case detection rates between men and women [18].

### 3.6. Individual-Level Delays

Almost half of the included studies (42%) appraised gender-related differences in individual-level delays. Of these, 38 found that symptomatic women were as likely as symptomatic men to delay or not seek TB services. However, among the 20 studies that found gender-related differences, 13 found that symptomatic women were more likely to delay or not seek TB services than symptomatic men, whereas seven studies found that symptomatic women were less likely to delay or not seek TB services than symptomatic men. The majority of studies were performed among study populations of persons who had already presented for care with diagnosed or suspected TB. Only five studies assessed persons with suspected TB in the general population. Of these, one study found that women were quicker to seek care for a prolonged cough [61], two studies found that women were slower to seek care [21, 97], and two studies found no difference in delay by gender [56, 111].

### 3.7. Provider-/System-Level Delays

Of 137 studies, 37 (27%) assessed gender-related differences in provider-/system-level delays in accessing TB services. The time between the presentation of a person with suspected TB to a health facility and TB diagnosis was most commonly assessed. Of 22 studies, 55% found no gender-related difference in the delay from presentation to TB diagnosis. All of the remaining 10 studies found that women experienced longer delays than men. Among 13 studies that examined the delay from presentation to TB treatment initiation, nine found no gender-related difference, three found that women had longer delays than men [14, 81, 135], and only one study found that men experienced longer delays than women [101]. Similarly, among seven studies that measured the delay between TB diagnosis and TB treatment initiation, four found no gender-related difference [33, 79, 104, 137], two found that women had longer delays than men [14, 19], and only one found that men had longer delays than women [35].

### 3.8. Combined Individual-/Provider-/System-Level Delays

Of 137 studies, 25 (18%) reported on gender-related differences in combined individual-/provider-/system-level delays. The delay between symptom onset and TB treatment initiation was most commonly assessed, and 13 out of these 18 (68%) studies found no gender-related difference. When a gender-related difference was observed, women faced longer delays than men [27, 79, 100, 140, 143]. One multicountry study found that, compared to men, women experienced longer delays in Yemen and shorter delays in Egypt but similar delays in other countries [141]. Among nine studies that assessed gender-related differences in the delay between symptom onset and TB diagnosis, 5 found no gender-related difference [33, 35, 114, 133, 146], whereas four studies found that women experienced longer delays than men [32, 36, 79, 142].

### 3.9. Quality of Included Studies

We assessed 126 cross-sectional studies, one case-control study, and eight cohort studies using the STROBE criteria [148], and we assessed two randomized trials using the CONSORT criteria [149, 150]. The majority of studies suffered from poor quality reporting of research design, methods, analyses, and results (see Supplementary Tables S2 and S3). Key weaknesses specific to and pervasive among the cross-sectional studies (92% of included studies) were inadequate reporting regarding the numbers of males and females at each study stage from eligibility assessment through enrollment, participation, follow-up, and analysis; explanation of nonparticipation for males and females at each stage; information on prevalence of exposures and confounders among the male and female participants; presentation of unadjusted and confounder-adjusted estimates for males and females; and explanation for selection of confounders for adjustment.

## 4. Discussion

Guided by a systematic review process, our review aimed to assess the quantitative evidence for gender-related differences in the barriers and delays that impact access to TB diagnostic and treatment services at the individual and provider/system levels. While, collectively, the included studies reported on barriers and delays at each level, more studies examined individual-level barriers and delays, and most studies surveyed persons presenting for care with diagnosed or suspected TB and were conducted in Africa and Asia. Overall, our review identified that many studies found no quantitative gender-related differences. However, when differences were reported, more studies found that women experienced greater barriers and longer delays at each level than men. In particular, many studies reported gender-related differences in financial, stigma, and health literacy barriers, which are interrelated and represent potential targets for gender-specific interventions that may be integrated into current and future TB service strategies.

While both genders experienced financial barriers to accessing TB services, the majority of studies that found gender-related differences reported that women experienced greater financial barriers than men, and the identified barriers were gender-specific. Specifically, the male role of primary income earner in many households prevented men from leaving work to access TB services, whereas, for women, their financial dependence on spouses and families limited access to TB services. Similar gender-related differences have been observed in financial barriers that limit access to diagnostic and treatment services for HIV and malaria [154–157]. Instituting more flexible hours and locations for TB services may help overcome the opportunity cost of lost wages and may improve case detection and treatment initiation among men. For women, barriers due to financial dependence may be compounded by the deprioritization of women's health care within the household below the needs of men and children. Because maternal health is prioritized by some households [158], efforts to integrate TB services with maternal healthcare may overcome some financial barriers and facilitate access to TB services among some women.

Regarding TB-related stigma, our review found that women were fearful of having a diagnosis of TB disclosed to their spouse, family, or community. Women experienced greater stigma than men, when gender-related differences were found. The impact of disease-related stigma has been well studied in the context of HIV, where anticipated or experienced stigma may lead patients to conceal symptoms, avoid or delay seeking care, hide their diagnoses, and be nonadherent with treatment [159–163]. Specifically, TB has been associated with dirtiness, immorality, substance abuse, and sexual promiscuity or deviancy [164–166], and, in communities with high rates of TB/HIV coinfection, TB may be further stigmatized by its association with HIV [167]. In addition to the psychosocial consequences of a TB diagnosis, our review also found that women were concerned about marital prospects and rejection by their spouse or families. Thus, TB-related stigma may also manifest as a financial barrier among those women who depend on spouses and family for financial support.

While stigma barriers may be addressed by interventions to improve TB-related health literacy, our review suggests that such programs may be particularly beneficial for women in rural areas. Among the included studies that reported gender difference in TB-related knowledge, men had greater TB-related knowledge and higher general literacy rates than women, and the majority of these (64%) were conducted in rural settings. It may be important to examine the interaction between female literacy and the impact of poverty on care seeking as this interaction has impacted care seeking among women in the context of other health services [168, 169].

Although only a few studies assessed the impact of barriers on delays, individual-level barriers appear to impact individual-level delays in TB care seeking in gender-specific ways. Symptomatic women were more likely to delay or not seek care than symptomatic men when gender-related differences in individual-level delays were reported. Individual-level TB-related stigma can represent both an obstacle and a motivation to seeking care [48], and marital status, which is intimately interlinked with issues of financial and social dependency as well as spousal and family support or rejection, also had a variable impact on gender-related differences in access to services [17, 71]. Regarding sociodemographic barriers, older age was a more significant barrier to accessing TB services among women than men [79, 81]. Given the complexity of these relationships, it is important to go beyond comparing the frequency and severity of individual-level barriers among women and men. Researchers and policymakers must also understand the impact of individual-level barriers on individual-level delays and how these barriers cause delays in accessing TB services among women and men. Qualitative studies may play an invaluable role here and inform researchers on the mechanisms of barriers and delays, which can be the points of intervention in the future.

Similarly, it is important to understand gender-related differences in provider-/system-level barriers and delays. In our review, fewer studies assessed barriers and delays at the provider/system level. However, when disparities were found, women were more likely to face barriers to accessing TB services than men. In addition, gender-specific individual barriers, such as financial and stigma barriers, may also impact the provider/system level but were not assessed by the studies included in our review. Surprisingly, in the context of other diseases, there are few reports on gender-related disparities in barriers and delays that limit access to care, particularly at the provider/system levels among patients in resource-limited settings. Provider-/system-level barriers and delays that lead to gender-related disparities in health often result from the lack of attention to the different needs of men and women while planning and providing health services, particularly with respect to service availability (e.g., geographical location, transportation available, service hours, and waiting time), affordability, acceptability (e.g., social and cultural competency, respect, privacy, confidentiality, and autonomy), and accountability [170, 171]. Furthermore, health providers and health systems may compound individual-level and community-level disparities by failing to recognize that gender-based differences exist or by failing to acknowledge the need for corrective interventions [1].

In addition to the paucity of data on barriers and delays at the provider/system levels, our review revealed several other research gaps. To comprehensively identify gender-related barriers and delays, study populations need to include persons with suspected TB who have not presented for care. There is also an urgent need for more granular analyses of gender disparities in accessing TB services for each step along the diagnostic and treatment continuum (i.e., symptom onset to symptom recognition; symptom recognition to seeking care; seeking care to TB diagnosis; TB diagnosis to notification; and notification to treatment initiation) at all levels. More generally, prospectively designed gender analyses are needed, and standardized ethnographic and cultural epidemiologic tools [5] also need to be used prospectively to systematically collect and compare gender-related sociocultural variables across studies, which may help to identify common as well as unique gender-related barriers.

The studies included in our review span different continents and differ among degree of urbanization and type of study population. Therefore, it is important to recognize heterogeneity while summarizing our findings. While most of the included studies were conducted in the Africa, South East Asia, and West Pacific regions, the frequency of some reported barriers by gender was not always proportional to numbers of studies from these regions. For example, financial barriers and delays at the individual and provider/system levels were reported proportionally by region, regardless of gender. However, women in South East Asia were noted to face more stigma, and women in West Pacific and both men and women in South East Asia had lower health literacy than persons from Africa (see Supplementary Table S4). These findings implicate region-specific priorities in interventions to improve access to TB care. Regarding study population type, included studies that assessed the general population (one quarter of the included studies) almost exclusively reported on stigma and health literacy barriers. Compared to studies among persons with diagnosed or suspected TB that found gender disparities, studies that assessed the general population were less likely to report that women face greater stigma and more likely to report that women have lower health literacy than men (see Supplementary Table S5). There is very little data to assess barriers and delays in different degrees of urbanization, as high percentage of studies were conducted in mixed urban and rural setting. However, studies from rural areas more frequently reported on worse health literacy among women (see Supplementary Table S6). The implication was already discussed above.

Many have called for more research on gender-related disparities in TB [4, 5, 8, 172, 173]. Accordingly, our systematic review aimed to assess the quantitative gender-related differences in barriers and delays that limit access to TB diagnostic and treatment services, which have been recognized as important for optimal TB control. However, a number of biases may have impacted our results and the individual studies that were included in our review. Although we strove to capture all high-quality studies addressing the topic of this review, some studies may have been missed, particularly those that were not published because they failed to document gender-related differences in accessing TB services, which may have resulted in an over representation of studies that demonstrated a difference (i.e., publication bias). In addition, our review was subject to biases introduced by the exclusion of non-English articles as studies from countries where English is not a primary language, particularly Latin American countries or East Asia, may be underrepresented. A noted limitation of the included studies was that the majority was cross-sectional studies and assessed patients with a confirmed TB diagnosis and/or those presenting for TB care. Those experiencing the greatest barriers to TB services are also least likely to be diagnosed with TB. Because persons presenting for care have already surmounted many individual-level barriers, comparisons of gender-related differences in these study populations will suffer from selection bias. In addition, sample size among the included studies was highly variable, and the quality of study reporting was generally poor. Finally, the summary measures and definitions of barriers and delays were inconsistently used, making it difficult to weigh the relative importance of findings from the included studies or to conduct a meta-analysis or stratified analysis.

## 5. Conclusions

Overall, the scientific community is recognizing that gender-related differences in health may be greater than is known and is increasingly prioritizing the need for routine gender-related analyses [174–177]. Notably, the WHO has developed a strategy to mainstream the analysis of the role of gender in health and to monitor and address systemic gender-related health inequities [178]. In the context of TB, gender analyses are critical to inform interventions to optimize the global impact of TB services. Our systematic review indicated that, when gender-related differences were found, women experienced greater barriers and longer delays than men and identified several gender-specific components within individual-level financial, stigma, and health literacy barriers that are amenable to intervention. However, our review also revealed research gaps and clearly highlighted that well-designed gender analyses are critical. Finally, qualitative accounts of the gender differences presented here would inform mechanisms of barriers and provide insight for interventions.

## Supplementary Material

Supplementary Material contains six data tables, which provide detailed summaries of various aspects of the included studies. Tables S1, S2 and S3 present individual study characteristics, the results of the quality assessment and individual study methodologies, respectively. Tables S4, S5, and S6 summarize WHO regional distribution, study population and degree of urbanization, respectively, by outcome type.

## Figures and Tables

**Table 1:** Characteristics of included studies.

Study characteristic	Description
Study design: *n* (%)	Clustered randomized trial: 1 (<1%); pragmatic randomized clinical trial: 1 (<1%); cohort study: 8 (6%); case-control study: 1 (<1%); cross-sectional study: 126 (92%)

Study population: *n* (%)	Individuals with diagnosed/suspected TB who presented to care: 76%; individuals in the community or population: 24%

Year of publication: *n* (%)	2000–2010: 123 (90%); 1990–1999: 11 (8%); 1980–1989: 2 (1%); 1970–1979: 1 (1%)

WHO regional distribution: *n* (%)	AFRO: 37 (27%); SEARO: 31 (23%); WPRO: 25 (18%); AMRO: 17 (13%); EMRO: 12 (9%); EURO: 11 (8%); multiple regions: 4 (3%)

Sample size	Range: 39–209,560,379; median (IQR): 335 (190–1,000)

Proportion of women	Range: 23–73%; median (IQR): 42% (34–49%)

AFRO: African region; AMRO: region of the Americas; EMRO: Eastern Mediterranean region; EURO: European region; IQR: interquartile range; SEARO: South East Asia region; TB: tuberculosis; WHO: World Health Organization; WPRO: Western Pacific region.

**Table 2:** Summary of quantitative gender-related findings by outcome type.

Outcome type	Number of studies	Gender difference				No gender difference	
		Women > Men		Men > Women			
		*n* (%)	List of studies	*n* (%)	List of studies	*n* (%)	List of studies
Individual-level barriers							
Financial	21^a^	11 (52%)	[12–14], [15]^a^, [16–22]	5 (24%)	[23, 24], [15]^a^, [25, 26]	6 (29%)	[27–32]
Physical	9	1 (11%)	[14]			8 (89%)	[26, 30–36]
Stigma^b^	25	11 (44%)	[17, 18, 22, 37–44]	2 (8%)	[45, 46]	12 (48%)	[24–26, 35, 47–54]
Health literacy	49	17 (35%)	[26, 34–36, 38, 41, 44, 50, 55–63]	8 (16%)	[24, 28, 40, 42, 43, 64–66]	24 (50%)	[14, 20, 22, 25, 30, 37, 45–47, 51–53, 67–78]
Sociodemographic	6	4 (67%)	[17, 79–81]			2 (33%)	[36, 71]
Provider-/system- level barriers	19	8 (42%)	[17, 29, 37, 82–86]			11 (58%)	[15, 28, 34, 35, 52, 73, 75, 87–90]
Combined individual-, provider-, and system-level barriers	7	5 (72%)	[29, 91–94]	1 (14%)	[95]	1 (14%)	[18]
Individual-level delay	58	13 (22%)	[14, 17, 21, 51, 73, 79, 96–102]	7 (12%)	[37, 61, 103–107]	38 (66%)	[16, 18–20, 28, 30–32, 36, 56, 71, 81, 108–133]
Provider-/system-level delay	37	11 (30%)	[14, 19, 20, 36, 81, 85, 120, 128, 131, 134, 135]	2 (5%)	[35, 101]	24 (65%)	[16, 18, 32, 33, 79, 96, 99, 100, 104, 106, 107, 113, 115, 117, 118, 121, 122, 124, 132, 133, 136–139]
Combined individual-, provider-, and system-level delay	25^c^	9 (36%)	[140], [141]^c^, [27, 32, 36, 79, 100, 142, 143]	1 (4%)	[141]^c^	17 (68%)	[33, 69, 110], [141]^c^, [35, 86, 114, 117, 124, 129, 131–133, 144–147]

^a^This study is included in both gender difference categories as it reported that the direct costs of seeking care were higher for men and that the household costs of seeking care were higher for women.

^b^One study was not included because the direction of association between gender and stigma could not be assessed [30].

^c^This study is included in all three gender-related finding columns as it is a multicountry study and reported gender-related findings that differed from country to country.
