# Supplementary material for: Barriers and Delays in Tuberculosis Diagnosis and Treatment Services: Does Gender Matter?
Source: Tuberc Res Treat. 2014 Apr 28;2014:461935. doi: 10.1155/2014/461935 (PMC4020203; doi:10.1155/2014/461935)
Supplement: Supplementary file 1 — Supplementary Material contains six data tables, which provide detailed summaries of various aspects of the included studies. Tables S1, S2 and S3 present individual study characteristics, the results of the quality assessment and individual study methodologies, respectively. Tables S4, S5, and S6 summarize WHO regional distribution, study population and degree of urbanization, respectively, by outcome type. [file 461935.f1.zip › SearchStrategy.pdf]

Search Strategy

Search date: 10/03/2010

MEDLINE

Tuberculosis AND Women:

"Tuberculosis"[Mesh] OR "tuberculosis"[All Fields] OR "TB"[All Fields] OR "tuberculous"[All Fields] OR "Tuberculosis, Multidrug-Resistant"[Mesh] OR "multi drug resistant tuberculosis"[All Fields] OR "MDR TB"[All Fields] OR "Extensively Drug-Resistant Tuberculosis"[Mesh] OR "extensively drug resistant tuberculosis"[All Fields] OR "XDR TB"[All Fields] OR "latent tuberculosis infection"[All Fields] OR "LTBI"[All Fields] AND ("Women"[Mesh] OR "Women"[TIAB] OR "woman"[TIAB] OR "Female"[Mesh] OR "female"[All Fields] OR "females"[All Fields] OR "maternal"[All Fields] OR "maternity"[All Fields] OR "motherhood"[All Fields] OR "Pregnant Women"[Mesh] OR "Pregnancy"[Mesh] OR "Breast Feeding"[Mesh] OR "Abortion, Induced"[Mesh] OR "Abortion, Spontaneous"[Mesh] OR "pregnancy"[All Fields] OR "pregnant"[All Fields] OR "abortion"[All Fields] OR "miscarriage"[All Fields] OR "breastfeeding"[All Fields])

Theme: Health care utilization and impact factors

"health services"[MeSH Terms:noexp] OR "Maternal Health Services"[Mesh] OR "Delivery of Health Care"[Mesh:noexp] OR "Health Services Accessibility"[Mesh] OR "Patient Acceptance of Health Care"[Mesh] OR "Health Status Disparities"[Mesh] OR "Healthcare Disparities"[Mesh] OR "Family Health"[Mesh] OR "Infectious Disease Transmission, Vertical"[Mesh] OR "Rape"[Mesh] OR "Violence"[Mesh:noexp] OR "Domestic Violence"[Mesh] OR "Poverty"[Mesh] OR "resource limited"[All Fields] OR "Unemployment"[Mesh] OR "Social Problems"[Mesh] OR "Substance-Related Disorders"[Mesh] OR "Malnutrition"[Mesh] OR "Socioeconomic Factors"[Mesh] OR "Social Support"[Mesh] OR "Depression"[Mesh] OR "Health Knowledge, Attitudes, Practice"[Mesh] OR "Healthcare utilization"[All Fields] OR "healthcare seeking"[All Fields] OR "health services"[All Fields] OR "health service"[All Fields] OR "health care utilization"[All Fields] OR "health care seeking"[All Fields] OR "health seeking"[All Fields] OR "health disparities"[All Fields] OR "family health"[All Fields] OR "barriers"[All Fields] OR "barrier"[All Fields] OR "prevention of mother-to-child transmission"[All Fields] OR "PMTCT"[All Fields] OR "antenatal"[All Fields] OR "prenatal"[All Fields] OR "perinatal"[All Fields] OR "stigma"[All Fields] OR "stigmatization"[All Fields] OR "taboo"[All Fields] OR "drug use"[All Fields] OR "drug abuse"[All Fields] OR "assault"[All Fields] OR "depression"[All Fields] OR "unemployment"[All Fields] OR "socioeconomic"[All Fields] OR "psychosocial"[All Fields] OR "caregiver"[All Fields] OR "poverty"[All Fields] OR "malnutrition"[All Fields] OR "social problems"[All Fields] OR "substance abuse"[All Fields] OR "violence"[All Fields] OR "rape"[All Fields] OR (("perception"[tiab] OR "perceptions"[tiab] OR "behavior"[tiab] OR "behaviour"[tiab] OR "costs"[tiab] OR "cost"[tiab] OR "knowledge"[tiab] OR "prejudice"[tiab] OR "misconception"[tiab]) AND ("TB"[tiab] OR "tuberculosis"[tiab]))

All Limiters Filter:

NOT (case reports[pt] OR editorial[pt] OR comment[pt] OR practice guideline[pt]) NOT ("animals"[MeSH Terms] NOT ("humans"[MeSH Terms] AND "animals"[MeSH Terms])) AND English[lang]

4430 (run 10/3/2010)

EMBASE

'tuberculosis'/exp OR 'tuberculosis' OR 'tb' OR 'tuberculous' OR 'multidrug resistant tuberculosis'/exp OR 'multi drug resistant tuberculosis' OR 'mdr tb' OR 'extensively drug resistant tuberculosis'/exp OR 'extensively drug resistant tuberculosis' OR 'xdr tb' OR 'latent tuberculosis infection' OR 'ltbi' AND ('women' OR 'woman' OR 'female'/de OR 'female' OR 'females' OR 'maternal' OR 'maternity' OR 'motherhood' OR 'pregnant woman'/exp OR 'pregnant woman' OR 'pregnant women' OR 'pregnancy'/exp OR 'pregnancy' OR 'pregnant' OR 'breast feeding'/exp OR 'breast feeding' OR 'abortion'/exp OR 'abortion' OR 'spontaneous abortion'/exp OR 'miscarriage')

AND

'health service'/de OR 'maternal welfare'/exp OR 'health care delivery'/exp OR 'health services accessibility':ab,ti OR 'patient attitude'/exp OR 'patient acceptance of health care':ab,ti OR 'health disparity'/exp OR 'health care disparity'/exp OR 'family health'/exp OR 'vertical transmission'/exp OR 'rape'/exp OR 'violence'/de OR 'domestic violence'/exp OR 'poverty'/exp OR 'unemployment'/exp OR 'social problem'/exp OR 'social problem':ab,ti OR 'drug dependence'/exp OR 'addiction'/de OR 'malnutrition'/exp OR 'socioeconomics'/exp OR 'social support'/exp OR 'depression'/exp OR 'attitude to health'/exp OR 'healthcare utilization':ab,ti OR 'healthcare seeking':ab,ti OR 'health services':ab,ti OR 'health service':ab,ti OR 'health care utilization'/exp OR 'health care utilization':ab,ti OR 'health care seeking':ab,ti OR 'health seeking':ab,ti OR 'health disparity':ab,ti OR 'health disparities':ab,ti OR 'health care disparity':ab,ti OR 'family health':ab,ti OR 'barriers':ab,ti OR 'barrier':ab,ti OR 'prevention of mother-to-child transmission':ab,ti OR 'pmtct':ab,ti OR 'prenatal care'/exp OR 'antenatal':ab,ti OR 'prenatal':ab,ti OR 'perinatal':ab,ti OR 'stigma'/exp OR 'stigma':ab,ti OR 'stigmatization':ab,ti OR 'taboo'/exp OR 'taboo':ab,ti OR 'drug use'/de OR 'drug use':ab,ti OR 'drug abuse'/exp OR 'drug abuse':ab,ti OR 'assault'/exp OR 'assault':ab,ti OR 'depression':ab,ti OR 'unemployment':ab,ti OR 'socioeconomic':ab,ti OR 'psychosocial':ab,ti OR 'caregiver burden'/exp OR 'caregiver':ab,ti OR 'poverty':ab,ti OR 'malnutrition':ab,ti OR 'social problems':ab,ti OR 'substance abuse':ab,ti OR 'violence':ab,ti OR 'rape':ab,ti OR 'resource limited':ab,ti OR ('perception':ab,ti OR 'perceptions':ab,ti OR 'behavior':ab,ti OR 'behaviour':ab,ti OR 'cost of illness'/exp OR 'health care cost'/exp OR 'costs':ab,ti OR 'cost':ab,ti OR 'knowledge':ab,ti OR 'prejudice':ab,ti OR 'misconception':ab,ti AND ('tb':ab,ti OR 'tuberculosis':ab,ti))

8547 (run 10/3/2010)

NOT ('animal'/exp NOT ('animal'/exp AND 'human'/exp)) NOT ('editorial':it OR 'note':it OR 'letter':it) AND [english]/lim

---

CINAHL

(MH "Tuberculosis+") OR tuberculosis OR tuberculous OR TB OR (MH "Tuberculosis, Multidrug-Resistant") OR "extensive drug resistant tuberculosis" OR "extensively drug resistant tuberculosis" OR "latent tuberculosis infection" OR "MDR TB" OR "LTBI" OR "XDR TB"

AND

(MH "Female") OR (MH "Women") OR woman OR women OR female OR females OR "pregnant women" OR "pregnant woman" OR (MH "Motherhood") OR maternity OR motherhood OR maternal OR (MH "Breast Feeding") OR "breast feeding" OR (MH "Abortion, Induced+") OR (MH "Abortion, Spontaneous") OR (MH "Abortion, Habitual") OR (MH "Abortion, Incomplete") OR (MH "Abortion, Criminal") OR abortion OR miscarriage

AND

(MH "Health Services Accessibility+") OR barrier OR barriers OR (MH "Health Services+") OR (MH "Health Care Delivery+") OR "health disparities" OR "health disparity" OR (MH "Family Health") OR "family health" OR (MH "Maternal Health Services+") OR (MH "Disease Transmission, Vertical") OR (MH "Rape") OR rape OR (MH "Violence+") OR (MH "Domestic Violence+") OR violence OR (MH "Poverty+") OR poverty OR "resource limited" OR (MH "Socioeconomic Factors+") OR "socioeconomic factors" OR "psychosocial factors" OR (MH "Unemployment") OR unemployment OR (MH "Social Problems+") OR "social problems" OR (MH "Substance Use Disorders+") OR (MH "Malnutrition") OR malnutrition OR (MH "Support, Psychosocial+") OR "social support" OR (MH "Depression+") OR depression OR (MH "Health Knowledge") OR "health knowledge" OR "health care utilization" OR "health seeking" OR "health care seeking" OR "prevention of mother to child transmission" OR "PMCTC" OR "antenatal care" OR "prenatal care" OR "perinatal care" OR (MH "Prenatal Care") OR (MH "Stigma") OR stigma OR stigmatization OR taboo OR (MH "Substance Abuse") OR "drug abuse" OR "drug use" OR (MH "Assault and Battery") OR assault OR (MH "Attitude to Health+") OR (MH "Attitude to Illness+") OR (MH "Caregiver Burden") OR caregiver

866 (limited to English only; run 10/3/2010)

Global Health 10/11/10 – 893 hits

Search Code: [mp=abstract, title, original title, broad terms, heading words]

<u>TB/Women terms:</u>
(exp miliary tuberculosis/ or exp extrapulmonary tuberculosis/ or exp tuberculosis/ or exp Mycobacterium tuberculosis/ or (tuberculosis or tuberculous or tb or xdr td or ltbi).mp) AND (exp

women/ or exp females/ or exp maternity/ or exp pregnant adolescents/ or exp induced abortion/ or exp spontaneous abortion/ or exp abortion/ or exp mycotic abortion/ or exp pregnancy/ or exp breast feeding/ or (women or woman or female or females or maternal or maternity or motherhood or pregnant or abortion or miscarriage or pregnancy or breast feeding).mp)

<u>Healthcare/utilization terms:</u>
health services/ or health services.mp or health service.mp. or maternal health services.mp. or exp maternity services/ or maternity services.mp. or delivery of health care.mp. or health services accessibility.mp. or exp access/ or patient acceptance.mp. or exp disparity/ or disparity.mp or family health.mp. or infectious disease transmission.mp. or exp disease transmission/ or disease transmission.mp. or exp vertical transmission/ or vertical transmission.mp. or exp maternal transmission/ or maternal transmission.mp. or exp rape/ or rape.mp. or violence.mp. or exp aggressive behaviour/ or aggressive behavior.mp or domestic violence.mp. or exp poverty/ or poverty.mp or resource limited.mp. or exp unemployment/ or unemployment.mp. or social problems.mp. or substance related disorders.mp. or exp drug abuse/ or drug abuse.mp. or exp substance abuse/ or substance abuse.mp. or exp drug addiction/ or drug addiction.mp. or exp malnutrition/ or malnutrition.mp or socioeconomic factors.mp. or exp socioeconomic status/ or socioeconomic status.mp. or exp low income groups/ or low income groups.mp. or exp socioeconomics/ or socioeconomics.mp. or exp economic depression/ or social support.mp. or exp support systems/ or support systems.mp. or exp depression/ or depression.mp. or exp inbreeding depression/ or healthcare utilization.mp. or health care utilization.mp. or exp resource utilization/ or utilization/ or utilization.mp. or healthcare seeking.mp. or health care seeking.mp. or health seeking.mp. or health disparities.mp. or family health.mp. or exp barriers/ or barriers.mp. or barrier.mp. or prevention of mother to child transmission.mp. or PMTCT.mp. or antenatal.mp. or exp prenatal diagnosis/ or exp prenatal screening/ or exp prenatal development/ or exp prenatal period/ or exp prenatal education/ or prenatal.mp. or perinatal.mp. or exp stigma/ or exp social stigma/ or stigma.mp. or stigmatization.mp. or taboo.mp. or exp aggressive behaviour/or aggressive behaviour.mp. or assault.mp. or psychosocial.mp. or exp psychosocial aspects/ or caregiver.mp. or exp perception/or perception.mp. or exp self perception/ or exp role perception/ or exp behaviour/ or behaviour.mp. or behavior.mp. or exp health care costs/ or exp costs/ or cost.mp. or costs.mp. or exp knowledge/ or knowledge.mp. or exp knowledge level/ or prejudice.mp. or exp discrimination/ or discrimination.mp. or misconception.mp. or exp health beliefs/or health beliefs.mp

Note: Excluded editorials (0) and correspondence (13)

Popline

Tuberculosis

AND

"health service" / "health services" / "maternal health services" / "delivery of health services" / "Delivery of Health Care" / "health services accessibility" / "patients acceptance of health care" / "patient acceptance of health care" / TX patient acceptance of health care / "health care services" / "health care service" / "Health Status Disparities" / "Health Status Disparities" / disparity / Disparities / "Family Health" / "Infectious Disease Transmission" / "Vertical transmission" / "maternal transmission" / "prevention of mother to child transmission" / PMTCT / Rape / Violence / "domestic violence" / SU

Poverty / "resource limited" / Unemployment / "Social Problems" / "Substance Related Disorders" / "substance use disorders" / "substance abuse" / Malnutrition / "Socioeconomic Factors" / socioeconomic / "Social Support" / "social problems" / Depression / "Health Knowledge Attitudes Practice" / utilization / "health care seeking behavior" / "health care seeking" / "healthcare seeking" / "health seeking behavior" / "health seeking behaviours" / "health seeking" / "health disparities" / barriers / barrier / antenatal / prenatal / perinatal / perinatal / stigma / stigmatization / discrimination / discrimination / taboo / "drug use" / "drug abuse" / assault / psychosocial / psychosocial / caregiver / (perception / behavior / behaviour / costs / cost / knowledge / prejudice / misconceptions)

AND

Women / woman / female / females / maternal / maternity / motherhood / Pregnant / Pregnancy / Breast Feeding / breastfeeding / abortion / miscarriage

AND

English language

Africa Wide
Limited to English

Tuberculosis AND Women:

(SU tuberculosis or TX tuberculosis or SU tb or TX tb or SU tuberculous or TX tuberculous or SU "MDR TB" or TX "MDR TB" or SU "XDR TB" or TX "XDR TB" or SU "LTBI" or TX "LTBI") and (SU Women or TX Women or SU woman or TX woman or SU female or TX female or SU females or TX females or SU maternal or TX maternal or SU maternity or TX maternity or SU motherhood or TX motherhood or SU Pregnant or TX Pregnant or SU Pregnancy or TX Pregnancy or SU Breast Feeding or TX Breast Feeding or SU breastfeeding or TX breastfeeding or SU abortion or TX abortion or SU miscarriage or TX miscarriage)

Theme: Health care utilization, family impact factors

SU “health service” or TX “health service” or SU “health services” or TX “health services” or SU “health care services” or TX “health care services” or SU “health care service” or TX “health care service” or SU “maternal health services” or TX “maternal health services” or SU “delivery of health services” or TX “delivery of health services” or SU “Delivery of Health Care” or TX “Delivery of Health Care” or SU “health services accessibility” or TX “health services accessibility”

SU “patients acceptance of health care” or TX “patients acceptance of health care” or SU “patient acceptance of health care” or TX “patient acceptance of health care” or SU “Health Status Disparities” or TX “Health Status Disparities” or SU “health disparities” or TX “health disparities” or SU disparity or TX disparity or SU Disparities or TX Disparities

SU “Family Health” or TX “Family Health” or SU “Infectious Disease Transmission” or TX “Infectious Disease Transmission” or SU” Vertical transmission” or TX “Vertical transmission” or SU “maternal transmission” or TX “maternal transmission” or SU “prevention of mother-to-child transmission” or TX

“prevention of mother-to-child transmission” or SU PMTCT or TX PMTCT or SU Rape or TX Rape or SU Violence or TX violence or SU “domestic violence” or TX “domestic violence” or SU Poverty or TX poverty

SU “resource limited” or TX “resource limited” or SU Unemployment or TX unemployment or SU “Social Problems” or TX “social problems” or SU “Substance Related Disorders” or TX “Substance Related Disorders” or SU “substance use disorders” or TX “substance use disorders” or SU “substance abuse” or TX “substance abuse” or SU Malnutrition or TX Malnutrition or SU “Socioeconomic Factors” or TX “socioeconomic factors” or SU socioeconomic or TX socioeconomic or SU “Social Support” or TX “Social Support” or SU “social problems” or TX “social problems” or SU Depression or TX Depression or SU “Health Knowledge, Attitudes, Practice” or TX “Health Knowledge, Attitudes, Practice” or SU utilization or TX utilization

SU “health care seeking behavior” or TX “health care seeking” or TX “healthcare seeking” or SU “health seeking behavior” or SU “health seeking behaviours” or TX “health seeking” or SU barriers or TX barriers or SU barrier or TX barrier or SU antenatal or TX antenatal or SU prenatal or TX prenatal or SU perinatal or TX perinatal or SU stigma or TX stigma or SU stigmatization or TX stigmatization or SU discrimination or TX discrimination or SU taboo or TX taboo or SU “drug use” or TX “drug use” or SU “drug abuse” or TX “drug abuse” or SU assault or TX assault or SU psychosocial or TX psychosocial or SU caregiver or TX caregiver

(TI perception or AB perception or TI behavior or AB behavior or TI behaviour or AB behavior or TI costs or AB costs or TI cost or AB cost or TI knowledge or AB knowledge or TI prejudice or AB prejudice or TI misconceptions or AB misconceptions) and (TI tuberculosis or AB tuberculosis or TI tb or AB tb)

LILACS

http://bases.bireme.br/cgi-bin/wxislind.exe/iah/online/?IsisScript=iah/iah.xis&base=LILACS&lang=i&form=F

("Tuberculosis" OR "TB" OR "tuberculous" OR "MDR TB" OR "XDR TB" OR "LTBI") [Words] **and**
("Women" OR "woman" OR "female" OR "females" OR "maternal" OR "maternity" OR "motherhood" OR "Pregnant" OR "Pregnancy" OR "Breast Feeding" OR "Abortion" OR "miscarriage" OR "breastfeeding") [Words] **and**
english [Language]

Web of Science

Tuberculosis AND Women:

TS=("Tuberculosis" OR "TB" OR "tuberculous" OR "MDR TB" OR "XDR TB" OR "LTBI") AND TS= ("Women" OR "woman" OR "female" OR "females" OR "maternal" OR "maternity" OR "motherhood" OR "Pregnant" OR "Pregnancy" OR "Breast Feeding" OR "Abortion" OR "miscarriage" OR "breastfeeding")

Theme: Health care utilization, family impact factors

TS=("health services" OR "Delivery of Health Care" OR "Patient Acceptance of Health Care" OR "Health Status Disparities" OR "Healthcare Disparities" OR "Family Health" OR "Infectious Disease Transmission " OR "Vertical transmission" OR " maternal transmission" OR "Rape" OR "Violence" OR "Poverty" OR "resource limited" OR "Unemployment" OR "Social Problems" OR "Substance-Related Disorders" OR "Malnutrition" OR "Socioeconomic Factors" OR "Social Support" OR "Depression")

TS=("Health Knowledge, Attitudes, Practice" OR "Healthcare utilization" OR "healthcare seeking" OR "health services" OR "health service" OR "health care utilization" OR "health care seeking" OR "health seeking" OR "health disparities" OR "family health" OR "barriers" OR "barrier" OR "prevention of mother-to-child transmission" OR "PMTCT" OR "antenatal" OR "prenatal" OR "perinatal" OR "stigma" OR "stigmatization" OR "taboo" OR "drug use" OR "drug abuse" OR "assault" OR "depression" OR "unemployment" OR "socioeconomic" OR "psychosocial" OR "caregiver" OR "malnutrition" OR "social problems" OR "substance abuse")

TS=(("perception" OR "perceptions" OR "behavior" OR "behaviour" OR "costs" OR "cost" OR "knowledge" OR "prejudice" OR "misconception") AND ("TB"OR "tuberculosis"))

Note: Excluded   NOTE (7)   EDITORIAL MATERIAL (13)   LETTER (2)
Limited to English

Cochrane

<u>Tuberculosis AND Women terms:</u>
"Tuberculosis"[Mesh]
"Tuberculosis, Multidrug-Resistant"[Mesh]
"Extensively Drug-Resistant Tuberculosis"[Mesh]

"tuberculosis" OR "TB" OR "tuberculous" OR "LTBI"

AND

"Women"[Mesh]
"Pregnant Women"[Mesh]
"Pregnancy"[Mesh]
"Breast Feeding"[Mesh]
"Abortion, Induced"[Mesh]
"Abortion, Spontaneous"[Mesh]

"Women"[TIAB] OR "woman"[TIAB]
"female" OR "females" OR "maternal" OR "maternity" OR "motherhood" OR "pregnancy" OR "pregnant" OR "abortion" OR "miscarriage" OR "breastfeeding"

<u>Health care utilization, family impact factors terms:</u>
"health services"[MeSH Terms:noexp]
"Maternal Health Services"[Mesh]
Delivery of Health Care[Mesh:noexp]

"Health Services Accessibility"[Mesh]
Patient Acceptance of Health Care[Mesh]
Health Status Disparities[Mesh] or "Healthcare Disparities"[Mesh]
"Family Health"[Mesh]
"Infectious Disease Transmission, Vertical"[Mesh]
"Rape"[Mesh]
Violence[Mesh:noexp]
"Domestic Violence"[Mesh]
"Poverty"[Mesh]
"Unemployment"[Mesh]
Social Problems[Mesh]
Substance-Related Disorders[Mesh]
"Malnutrition"[Mesh]
"Socioeconomic Factors"[Mesh]
Social Support[Mesh]
Depression[Mesh]
"Health Knowledge, Attitudes, Practice"[Mesh]

"health service" or "health services" or Rape or violence or poverty or "resource limited" or unemployment or "social problems" or "substance abuse" or "drug use" or "drug abuse" or malnutrition or socioeconomic or depression or "Healthcare utilization" or "health care utilization" or "healthcare seeking" or "health care seeking" or "health seeking" or "health disparities" or "family health" or barriers or barrier or "prevention of mother-to-child transmission" or PMTCT or antenatal or prenatal or perinatal or stigma or stigmatization or taboo or assault or psychosocial or caregiver
****
Limit to Title/Abstract
("perception" OR "perceptions" OR behavior OR "behaviour" OR "costs" OR "cost" OR knowledge" OR "prejudice" OR "misconception") AND (tuberculosis OR tb)

WHOLIS (title/phrase search and English)
http://www.who.int/library/databases/en/

Tuberculosis OR TB OR tuberculous AND Women OR woman OR female OR females OR maternal OR maternity OR motherhood OR Pregnant OR Pregnancy OR Breast Feeding OR Abortion OR miscarriage

Hand search – no export function

BASE (Bielefeld Academic Search Engine) is a multi-disciplinary search engine to scholarly internet resources, created by Bielefeld University Library in Bielefeld, Germany. It is based on search technology provided by Fast Search & Transfer (FAST), a Norwegian company.
http://base.ub.uni-bielefeld.de/en/index.php
tuberculosis female (doctype:0002 doctype:0001 doctype:0003 doctype:0004 doctype:0005 doctype:0106)
Refined to English

tuberculosis (female* or women or woman) (doctype:0002 doctype:0001 doctype:0003 doctype:0004 doctype:0005 doctype:0106)

Hand search – no export function

Eldis http://www.eldis.org/

Search tuberculosis (search box located in top left corner).
Refine by Document Type
Select Categories
Browse by Categories

Hand search – no export function
