# Supplementary material for: Barriers and Delays in Tuberculosis Diagnosis and Treatment Services: Does Gender Matter?
Source: Tuberc Res Treat. 2014 Apr 28;2014:461935. doi: 10.1155/2014/461935 (PMC4020203; doi:10.1155/2014/461935)
Supplement: Supplementary file 1 — Supplementary Material contains six data tables, which provide detailed summaries of various aspects of the included studies. Tables S1, S2 and S3 present individual study characteristics, the results of the quality assessment and individual study methodologies, respectively. Tables S4, S5, and S6 summarize WHO regional distribution, study population and degree of urbanization, respectively, by outcome type. [file 461935.f1.zip › Table S1.pdf]

TableS1. Summary of included studies

First Author<br>Year<br>Country<br>Setting	Type of study<br>Sample size (N)<br>(% female)<br>Age(years)	Study population	Individual-level delay	Provider-/system-level delay	Type of provider-/system-delay	Combined individual-/provider-/system-level delay	Type of combined delay	Individual-level barriers					Provider-/system-level barriers	Combined individual-/provider-/system-level barrier
								Financial	Physical	Stigma	Health literacy	Sociodemographic		
CROSS-SECTIONAL STUDY														
Cramm<br>2010<br>South Africa<br>Unknown	Cross-sectional<br>1042<br>73.0%<br>Median=38	General population	-	-	-	-	-	-	-	No significant difference in attitude toward people with TB between men and women, however, more women reported their fear that people would talk about it as a reason to delay clinic visit (OR: 1.142, p<0.05)	No significance difference in TB knowledge score between men and women (p=0.083)	-	-	-
Deribew<br>2010<br>Ethiopia<br>Mixed	Cross-sectional<br>750<br>58.0%<br>Mean<br>(SD)=31.2(11.0)	General population	-	-	-	-	-	-	-	A larger proportion of females than males had high prejudice toward patients with TB (aOR: 1.3, 95% CI 1.0, 1.9)	Men were more likely to recognize that weight loss, shortness of breath, chest pain, and poor appetite are TB symptoms (47.2% vs 39.0% p=0.029, 20.5% vs 14.8% p=0.05, 20.8% vs 13.1% p=0.006, 20.8% vs 13.3% p=0.02).	-	-	-

TableS1. Summary of included studies

First Author<br>Year<br>Country<br>Setting	Type of study<br>Sample size (N)<br>(% female)<br>Age(years)	Study population	Individual-level delay	Provider-/system-level delay	Type of provider-/system-delay	Combined individual-/provider-/system-level delay	Type of combined delay	Individual-level barriers					Provider-/system-level barriers	Combined individual-/provider-/system-level barrier
								Financial	Physical	Stigma	Health literacy	Sociodemographic		
Dhingra<br>2010<br>India<br>Urban	Cross-sectional<br>1977<br>42.1%<br>-	New PTB patients;<br>EPTB patients	-	-	-	-	-	-	-	Family level:<br>No significant difference by gender in disclosure of disease in their family (M 98.9% vs F 99.6% p>0.05) or worried about marriage prospect among unmarried patients (M 73% vs W 63.5%, p>0.05) Females were less likely than males to share TB information with family members (52.2% vs 64.9%, p<0.05) and were more likely to experience spouse's change of attitude toward them (11.4% vs 5.5% p<0.05)<br>Work level:<br>Significantly less females disclosed their disease status to colleagues and employers (p<0.05)<br>Society level:<br>Intense stigma was observed with 60% of patients hiding their disease, stigma at this level was found to be more in females than in males (p<0.05)	-	-	-	-
Mesfin[1]<br>2010<br>Ethiopia<br>Mixed	Cross-sectional<br>924<br>45%<br>Median=34 years	New TB cases	-	-	-	-	-	Total cost per person diagnosed is higher in women, because women incurred higher escort-related costs	-	-	-	-	-	-
Mushtaq<br>2010<br>Pakistan<br>Mixed	Cross-sectional<br>1080<br>23.5%<br>61.6% between 31 and 50 years	General population	-	-	-	-	-	-	-	--	No significant difference in good TB knowledge (male 41.2% vs female 47.2%), p=0.095	-	-	-

TableS1. Summary of included studies

First Author<br>Year<br>Country<br>Setting	Type of study<br>Sample size (N)<br>(% female)<br>Age(years)	Study population	Individual-level delay	Provider-/system-level delay	Type of provider-/system-delay	Combined individual-/provider-/system-level delay	Type of combined delay	Individual-level barriers					Provider-/system-level barriers	Combined individual-/provider-/system-level barrier
								Financial	Physical	Stigma	Health literacy	Sociodemographic		
Pungrassami<br>2010<br>Thailand<br>Unknown	Cross-sectional<br>432<br>34.3%<br>Median=37;range18-79	TB cases, including TB and HIV co-infection cases	-	-	-	-	-	-	-	In community perspective, men tended to delay diagnosis with higher stigma, whereas women tended not to delay diagnosis with higher stigma The difference was not statistically significant (p=0.05)	-	-	-	-
Vassal<br>2010<br>Ethiopia<br>Mixed	Cross-sectional<br>184<br>47.3%<br>Median: 25-34 years	TB or HIV cases	-	-	-	-	-	Men had higher indirect cost and women had higher direct cost	-	-	-	-	-	-
Atre[2]<br>2009<br>India<br>Rural	Cross-sectional<br>160<br>50.0%<br>Mean=39.7; 62%<45	Individuals without TB	-	-	-	-	-	-	-	No significant statistical difference in stigma indicators by gender and between male and female vignettes. However, there are qualitative differences	-	-	-	-
Basnet<br>2009<br>Nepal<br>Mixed	Cross-sectional<br>307<br>41.0%<br>-:	TB cases	Compared to males females had higher odds of delay >30 days (aOR= 1.2, CI= 0.73-1.98)	Compared to males, females had lower odds of delay , which was not significant (aOR= 0.8, CI= 0.44-1.37)	*	-	-	-	-	-	-	-	-	-
Berisha<br>2009<br>Kosovo<br>Mixed	Cross-sectional<br>155<br>57.4%<br>Mean= 45.47 (SD 18.71)	TB cases	-	-	-	-	-	-	-	98.9% of womenand 90.9% of men had informed a family member they had TB (p <0.01), while 1.1% of women vs 9% of men had informed a friend they had TB(p<0.01).	More females than males gave a correct answer to question on TB symptoms. 97.8% vs 87.9%; (p<0.05)	-	-	-
Ford<br>2009<br>Peru<br>Urban	Cross-sectional<br>108<br>40%<br>Median= 33 (IQR 21-46)	PTB cases	Male gender was associated with a 48% longer test-seeking delay (p=0.02).	-	-	-	-	-	-	-	-	-	-	-

TableS1. Summary of included studies

First Author<br>Year<br>Country<br>Setting	Type of study<br>Sample size (N)<br>(% female)<br>Age(years)	Study population	Individual-level delay	Provider-/system-level delay	Type of provider-/system-delay	Combined individual-/provider-/ system-level delay	Type of combined delay	Individual-level barriers					Provider-/system-level barriers	Combined individual-/provider-/system-level barrier
								Financial	Physical	Stigma	Health literacy	Sociodemographic		
French<br>2009<br>England<br>Mixed	Cross-sectional<br>22856<br>45%<br>Median= 36 (IQR 26-54)	TB cases	-	-	-	Females had statistically significant longer delays from symptom onset to treatment initiation than males. 71 days vs 65 days (HR= 0.9, p<0.001)	^	-	-	-	-	-	-	-
Gele<br>2009<br>Ethiopia<br>Rural	Cross-sectional<br>226<br>45.3%<br>Mean= 32.2(SD 13.0)	TB cases	There was no significant gender differences in patient delay	-	-	-	-	-	-	-	-	-	-	-
Hoa<br>2009<br>Vietnam<br>Rural	Cross-sectional<br>12143<br>58.3%<br>31.6% between 15-34; 43.6 % between 35-54; 24.8% >= 55	General population	-	-	-	-	-	-	-	Significantly more women associated heavy stigma with a TB diagnosis (48.7% vs 35.1%, p<0.0001)	Significantly higher knowledge score in men; more men knew TB mode of transmission, sputum smear exam, and free TB drugs (77.9%, 65.7% p<0.001, 66% vs 54.5% p<0.001, 57% vs 45.3% p<0.001); fewer men knew hemoptysis as a symptom of TB (15.4% vs 17.3%, p<0.05); higher mean knowledge score was associated with visiting a hospital (p<0.01)	-	-	-

TableS1. Summary of included studies

First Author<br>Year<br>Country<br>Setting	Type of study<br>Sample size (N)<br>(% female)<br>Age(years)	Study population	Individual-level delay	Provider-/system-level delay	Type of provider-/system-delay	Combined individual-/provider-/system-level delay	Type of combined delay	Individual-level barriers					Provider-/system-level barriers	Combined individual-/provider-/system-level barrier
								Financial	Physical	Stigma	Health literacy	Sociodemographic		
Lu<br>2009<br>China<br>Mixed	Cross-sectional<br>69253<br>51.0%<br>Mean (SD)=40.8 (13.4)	General population	-	-	-	-	-	-	-	No significant difference in stigma (M/F OR 1.0 95% CI 1.0-1.1)	Men had higher level of TB awareness (overall awareness OR 1.3 95% CI 1.2-1.3; symptom/sign OR 1.1 95% CI 1.1-1.2; Transmission mode OR 1.2 95% CI 1.2-1.2; Dispensary OR 1.1 95% CI 1.1-1.1; Detection/treatment OR 1.2 95% CI 1.2-1.3; curable disease OR 1.2 95% CI 1.1-1.2)	-	-	-
Mesfin[1]<br>2009<br>Ethiopia<br>Mixed	Cross-sectional<br>924<br>45.0%<br>Median=34	New TB cases	No significant difference in prolonged patient delay and gender (F/M aOR 1.4, 95% CI 0.9-1.8, p-value 0.06)	-	-	-	-	-	-	-	-	-	-	-
Ngadaya<br>2009<br>Tanzania	Cross-sectional<br>206<br>33.5%<br>Mean: 37.3 years for men; 33.7 for women	New SSM+ TB cases	No significant difference in patient delay (M/F OR 0.6 95% CI 0.32-1.10)	-	-	No significant difference in health facility delay (M/F OR 0.8 95%CI 0.44-1.44)	^	-	-	-	-	-	-	-
Yimer[3]<br>2009<br>Ethiopia<br>Rural	Cross-sectional<br>1006<br>53.9%<br>Mean=40 years	TB suspects	Women less likely to see medical health providers than men (aOR 0.8, 95% CI 0.6-0.9);no significant difference by gender in seeking any health provider (aOR 0.78, 95% CI 0.6-1.0)	-	-	-	-	-	-	-	-	-	-	-

TableS1. Summary of included studies

First Author<br>Year<br>Country<br>Setting	Type of study<br>Sample size (N)<br>(% female)<br>Age(years)	Study population	Individual-level delay	Provider-/system-level delay	Type of provider-/system-delay	Combined individual-/provider-/system-level delay	Type of combined delay	Individual-level barriers					Provider-/system-level barriers	Combined individual-/provider-/system-level barrier
								Financial	Physical	Stigma	Health literacy	Sociodemographic		
Yimer[3]<br>2009<br>Ethiopia<br>Rural	Cross-sectional<br>1006<br>53.9%<br>Mean=40 years	TB suspects	-	-	-	-	-	-	-	-	-	-	-	A higher proportion of TB patients detected by active case-finding were female (63.2%, p=0.04). Women had a 3.43 higher odds than men of being detected by ACF vs PCF (p=0.05).
Al-Maniri<br>2008<br>Oman<br>Urban	Cross-sectional<br>257<br>53.9%<br>Mean age 41.3	General Practitioners	-	-	-	-	-	-	-	-	-	-	No correlation between gender and TB Knowledge score (regression score -0.08, CI95% -0.94, 0.77)	-
Armijos<br>2008<br>Ecuador<br>Urban	Cross-sectional<br>212<br>62.7%<br>Range= 18-80	TB suspects	-	-	-	-	-	-	-	More women than men indicated that they would keep an employeewith TB and help them to get treatment (28.9% vs. 5.4%,P = 0.003).	Gender did not predict reported TB familiarity. More men than women (35.4% vs. 14.3%, P = 0.001) identified an infectious cause for TB. Men were lesslikely than women to indicate that TBcan be transmitted by fomites (41.7% vs 23.4%, p= 0.02).	-	-	-
Aspler<br>2008<br>Zambia<br>Urban	Cross-sectional<br>103<br>43.0%<br>Mean=32; range 18-53	PTB andEPTB cases	-	-	-	-	-	Total costs as a proportion of median monthly income were significantly higher for women than men (132% vs 10.1%). Pre-diagnosis costs as a proportion of median monthly income were higher for women than men (113% vs 7%).	-	-	-	-	-	-

TableS1. Summary of included studies

First Author<br>Year<br>Country<br>Setting	Type of study<br>Sample size (N)<br>(% female)<br>Age(years)	Study population	Individual-level delay	Provider-/system-level delay	Type of provider-/system-delay	Combined individual-/provider-/system-level delay	Type of combined delay	Individual-level barriers					Provider-/system-level barriers	Combined individual-/provider-/system-level barrier
								Financial	Physical	Stigma	Health literacy	Sociodemographic		
Ayuo<br>2008<br>Kenya<br>mixed	Cross-sectional<br>230<br>35.7%<br>Median= 28.5; range 12-80	TB cases	No significant difference in mean patient delay was found by gender.	-	-	-		-	-	-	No significant difference in level of education by gender (p=0.08)	Women were as likely as men to be widowed/separated/divorced (19.51% vs 12.16%, p=0.19); Being widowed/separated/divorced was a predictor of longer patient-level delay (p = 0.015).	-	-
Bassili<br>2008<br>Egypt, Iran, Iraq, Pakistan, Somalia, Syria, Yemen<br>Mixed	Cross-sectional<br>5053<br>39.7%<br>Majority 15-35	TB cases	-	-	-	Yemenese women had significantly longer total delay (adjusted OR 2.29, 95% CI 1.26-4.14) and Egyptian women had significantly shorter total delay (adjusted OR 0.62, 95% CI 0.39-0.99), whereas there were no difference by gender in delay among other countries.	^	-	-	-	-	-	-	-
Brassard<br>2008<br>Canada<br>Urban	Cross-sectional<br>164<br>48.8%<br>Mean= 38 (SD 10.8)	High-risk population	-	-	-	-	-	-	-	-	Females had higher odds of reporting no TB knowledge than males. This was not statistically significant. (OR= 1.6, CI= 0.8-3.1)	-	-	-
Chimbanrai<br>2008<br>Thailand<br>Mixed	Cross-sectional<br>300<br>42.7%<br>Mean= 39.9 (SD 14.9)	PTB cases	-	-	-	-	-	-	-	-	Males were found to have poorer TB knowledge than females.(P=0.001)	-	-	-

TableS1. Summary of included studies

First Author<br>Year<br>Country<br>Setting	Type of study<br>Sample size (N)<br>(% female)<br>Age(years)	Study population	Individual-level delay	Provider-/system-level delay	Type of provider-/system-delay	Combined individual-/provider-/system-level delay	Type of combined delay	Individual-level barriers					Provider-/system-level barriers	Combined individual-/provider-/system-level barrier
								Financial	Physical	Stigma	Health literacy	Sociodemographic		
Gosoniu[4]<br>2008<br>Bangladesh, India, Malawi<br>Rural(Bangladesh); Urban (India, Malawi)	Cross-sectional<br>329<br>49.0%<br>-	TB cases	-	-	-	Female sex in Bangladesh, housewife in Malawi, married women (but not men) in India were associated with problem delay from symptom onset to TB diagnosis	$	-	-	-	-	-	-	-
Kilale<br>2008<br>Tanzania<br>Urban	Cross-sectional<br>69<br>39.0%<br>Mean=33.2;range 11-72	TB cases	More men sought care within first 4-8 weeks of symptom onset	-	-	-	-	-	-	-	No significant difference in knowledge of TB transmission by gender (p=0.895)	-	No significant difference in perception of good patient-provider relationship as an important reason service satisfaction (p=0.623)	-
Lin<br>2008<br>China<br>Unknown	Cross-sectional<br>10356<br>34.5%<br>Median (IQR)=38 (27-54)	New SSM+ TB cases	No significant difference in patient delay (p=0.57)	-	-	-	-	-	-	-	-	-	-	-
Lorent<br>2008<br>Ruwanda<br>Urban	Cross-sectional<br>104<br>57.0%<br>Mean (range)=35 (17-84)	New TB cases	No significant difference in patient delay	No significant difference in health care system delay at referral hospital (M/F unadjusted OR 1.14 95% CI 0.52-2.50)	*	-	-	-	-	-	-	-	-	-

TableS1. Summary of included studies

First Author<br>Year<br>Country<br>Setting	Type of study<br>Sample size (N)<br>(% female)<br>Age(years)	Study population	Individual-level delay	Provider-/system-level delay	Type of provider-/system-delay	Combined individual-/provider-/system-level delay	Type of combined delay	Individual-level barriers					Provider-/system-level barriers	Combined individual-/provider-/system-level barrier
								Financial	Physical	Stigma	Health literacy	Sociodemographic		
Maamari<br>2008<br>Syria<br>Mixed	Cross-sectional<br>800<br>36.2%<br>Median (range)=27.5 (15-95)	New SSM+ PTB cases	No significant difference in patient related diagnostic delay (F/M aOR 1.20; 95% CI 0.78-1.86)	-	-	No significant difference in diagnostic delay (F/M aOR 1.11; 95% CI 0.71-1.74); no significant difference in total delay (F/M aOR 1.01; 95% CI 0.65-1.57)	^, $	-	-	-	-	-	-	-
Mahendradhata<br>2008<br>Indonesia<br>Mixed	Cross-sectional<br>421<br>42.3%<br>54.6% between 25-54 years	SSM+ TB cases	-	-	-	No significant difference in total delay (p=0.982)	^	-	-	-	-	-	-	-
Marks<br>2008<br>US<br>Mixed	Cross-sectional<br>209560379 (weighted)<br>52.0% (weighted)<br>-	General population	-	-	-	-	-	-	-	Men felt less ashamed if they or others had TB (aOR 0.8; 99% CI 0.8-0.9)	Men less heard of TB; knew someone with TB; less knowledgeable about TB, felt less at high risk of getting TB, and less felt ashamed if had TB (aORs all below 1 with 99% CI not covering 1.) No gender difference in that TB is spread by breathing (99%CI covers 1)	-	-	-
Meintjes<br>2008<br>South Africa<br>Unknown	Cross-sectional<br>104<br>62.5%<br>Median(IQR)=30(25-38.5)	New TB cases	Men had longer patient delay (M vs F median 30 vs 14 days, p_{adj}=0.02)	No significant difference in provider delay	#	-	-	-	-	-	-	-	-	-

TableS1. Summary of included studies

First Author<br>Year<br>Country<br>Setting	Type of study<br>Sample size (N)<br>(% female)<br>Age(years)	Study population	Individual-level delay	Provider-/system-level delay	Type of provider-/system-delay	Combined individual-/provider-/system-level delay	Type of combined delay	Individual-level barriers					Provider-/system-level barriers	Combined individual-/provider-/system-level barrier
								Financial	Physical	Stigma	Health literacy	Sociodemographic		
Mfinanga<br>2008<br>Tanzania<br>Urban	Cross-sectional<br>639<br>39.3%<br>Mean (SD)=35.1(12.1) years for males; 33.1(11.2) for females	SSM+ TB cases	Women had longer patient delay>30 days (41.0% vs 31.5%, p=0.019)	More women had referral delay >2 days (94.6% vs 86.2%, p=0.019); more women had diagnostic delay>3 days (62.1% vs 47.0%, p=0.001); more women had facility delay>5 days (88.8% vs 79.8%, p=0.005); no significant difference in treatment delay	*, #, @	-	-	Having no employment was associated diagnostic delay among women, but not men	Although distance from residence to facility with DOTS was similar for both men and women, distance>5Km was associated with more diagnostic delay among women, than men.	-	Poor knowledge of TB symptoms were associated with diagnostic delay among women, but not men	-	-	-
Continuation of Mfinanga<br>2008						-	-		Among patients living within 5km from the health facility the proportion of males vs females was 65.8 vs 61.6%; for distance 5-10km it was 85% vs 63%; for distance >10km it was 47% vs 33%	-		-	-	-
Qureshi<br>2008<br>Pakistan<br>Urban	Cross-sectional<br>301<br>46.8%<br>Median among females: 32<br>Median among males: 35	TB cases	Women had longer patient delay (median 35 days in women, 29 days in men, p<0.001).	-	-	-	-	-	-	27% reported the presence of stigma related to TB. Both men and women were affected by stigma	No significant difference in knowledge about of TB.	-	-	-

TableS1. Summary of included studies

First Author<br>Year<br>Country<br>Setting	Type of study<br>Sample size (N)<br>(% female)<br>Age(years)	Study population	Individual-level delay	Provider-/system-level delay	Type of provider-/system-delay	Combined individual-/provider-/system-level delay	Type of combined delay	Individual-level barriers					Provider-/system-level barriers	Combined individual-/provider-/system-level barrier
								Financial	Physical	Stigma	Health literacy	Sociodemographic		
Rumman<br>2008<br>Jordan<br>Mixed	Cross-sectional<br>1544<br>42.6%<br>37.3% between 15-35	General population; TB suspects	Female TB suspects were significantly more likely to delay healthcare-seeking (aOR 1.82, 95% CI 1.15-2.88)	-	-	-	-	-	-	-	-	-	-	-

TableS1. Summary of included studies

First Author<br>Year<br>Country<br>Setting	Type of study<br>Sample size (N)<br>(% female)<br>Age(years)	Study population	Individual-level delay	Provider-/system-level delay	Type of provider-/system-delay	Combined individual-/provider-/system-level delay	Type of combined delay	Individual-level barriers					Provider-/system-level barriers	Combined individual-/provider-/system-level barrier
								Financial	Physical	Stigma	Health literacy	Sociodemographic		
Somma[4]<br>2008<br>Bangladesh, India, Malawi and Colombia<br>Rural (Bangladesh)<br>Urban (Colombia, India, and Malawi)	Cross-sectional<br>427<br>~50%<br>: -	TB cases	-	-	-	-	-	-	-	Females in Bangladesh have a significantly higher stigma index (p=0.04); in India, the male-female difference was suggestive but not significant (p=0.01).<br>In Bangladesh and to a lesser degree in India, impaired self-esteem, shame/embarrassment and perceived low regard of others were likely to be reported by women.<br>Concern about social isolation was also more frequently reported by women in Bangladesh.<br>For women with low self-esteem and concern about social isolation in the South Asian sites, these feelings were often related to the experience of having been actively isolated because of their disease, or forced to move away from their homes.<br>Work-related aspects of stigma were frequently reported, and they were more likely to be an issue for men, except in Colombia where more women said they had decided to stay away from work and groups.<br>In the multivariate analysis, never having been married was associated with greater stigma in India and Malawi but increasing age in Bangladesh was associated with less stigma.	-	-	-	-

TableS1. Summary of included studies

First Author<br>Year<br>Country<br>Setting	Type of study<br>Sample size (N)<br>(% female)<br>Age(years)	Study population	Individual-level delay	Provider-/system-level delay	Type of provider-/system-delay	Combined individual-/provider-/system-level delay	Type of combined delay	Individual-level barriers					Provider-/system-level barriers	Combined individual-/provider-/system-level barrier
								Financial	Physical	Stigma	Health literacy	Sociodemographic		
Thierfelder<br>2008<br>Tajikistan<br>Mixed	Cross-sectional<br>1495<br>45.2%<br>Median=32 years, range 18-84 years	PTB cases	-	-	-	-	-	-	-	-	-	-	Men were more likely to receive treatment in hospital than women. (aOR 1.49 95% CI 1.08-2.05, p=0.02).	-
Thongraung<br>2008<br>Thailand<br>Mixed	Cross-sectional<br>383<br>23.5%<br>40.46% between 25-44	TB cases	-	-	-	-	-	-	-	-	-	-	Men were more likely than women diagnosed adherently to national guidelines (aOR 1.9, 95% CI 1.1-3.2, p=0.01)	-
Wang, J. M.<br>2008<br>China<br>Rural	Cross-sectional<br>1083 (study1); 171 (study2)<br>53.6% (study1); 42.1% (study2)<br>Mean(SD)=43.1(12.9) for study1	General population (study1); TB suspects (study2)	There was no significant gender difference in patient delay among the TB suspects. (p= 0.305)	-	-	-	-	-	-	-	More men actively learn knowledge of TB (20.1% vs 17.7%, p<0.001); significantly more men were aware of where to seek TB diagnosis and treatment, and free service; no significant difference in TB as a transmissible disease by gender (92.6% men vs 91.6%, p>0.05)	-	-	-
Wang, Y.<br>2008<br>China<br>Urban	Cross-sectional<br>1005<br>45.6%<br>33.5% between 15-44	TB suspects	Women were more likely than men to delay health care-seeking (aOR 1.76 95% CI 1.33-2.33).	-	-	-	-	-	-	-	-	-	-	-

TableS1. Summary of included studies

First Author<br>Year<br>Country<br>Setting	Type of study<br>Sample size (N)<br>(% female)<br>Age(years)	Study population	Individual-level delay	Provider-/system-level delay	Type of provider-/system-delay	Combined individual-/provider-/system-level delay	Type of combined delay	Individual-level barriers					Provider-/system-level barriers	Combined individual-/provider-/system-level barrier
								Financial	Physical	Stigma	Health literacy	Sociodemographic		
Weiss[4]<br>2008<br>Bangladesh, India, Malawi<br>Rural(Bangladesh); Urban (India, Malawi)	Cross-sectional<br>329<br>49.0%<br>-	TB cases	-	-	-	-	-	Men felt significantly more financial distress because of TB than women in all three countries (Bangladesh 63.5% vs14% , p<0.01; India: 75.8% vs 37.7% , p<0.01; Malawi: 78% vs 60% , p<0.05)	-	No quantitative difference in social barriers in all three countries.	Significant differences in knowledge about perceived causes were reported at different levels in all three countries (some cause-specific non-significant differences were also reported); more women knew TB is transmitted through environmental exposure either through infection, contact, or airborne exposure.	-	-	-
Chang<br>2007<br>Malaysia<br>Mixed	Cross-sectional<br>316<br>38.9%<br>Median=45; range: 15-70	TB cases	Women were significantly more likely to delay health seeking (57% vs 36%, p=0.006).	No significant difference in diagnostic delay between men and women (51% women vs 48% men, p=0.626)	-	-	-	-	-	-	-	-	-	-
Huong<br>2007<br>Vietnam<br>Mixed	Cross-sectional<br>2093<br>29.0%<br>56% between 25-54	TB cases	Women were significantly associated with longer patient delay (aOR 1.3, 95%CI 1.0-1.7)	No significant difference in health system delay (female vs male aOR 1.2, 95% CI 0.8-1.7); risk of long patient delay if the first provider was private rather than public was reduced by half among men, but to one third among women (test of interaction, p<0.05)	#	Women were significantly associated with longer total delay (aOR 1.6, 95% CI 1.3-2.2)	^	-	-	-	-	-	-	-

TableS1. Summary of included studies

First Author<br>Year<br>Country<br>Setting	Type of study<br>Sample size (N)<br>(% female)<br>Age(years)	Study population	Individual-level delay	Provider-/system-level delay	Type of provider-/system-delay	Combined individual-/provider-/system-level delay	Type of combined delay	Individual-level barriers					Provider-/system-level barriers	Combined individual-/provider-/system-level barrier
								Financial	Physical	Stigma	Health literacy	Sociodemographic		
Karim<br>2007<br>Bangladesh<br>Mixed	Cross Sectional<br>1000<br>50.0%<br>Mean= 37.7	TB cases	Women had significantly longer patient's delay than men.	No significant difference in doctor's, treatment , and health system's delays	*, #, @	Women had significantly longer total delay and total diagnostic delay than men.	^, $	-	-	-	-	Older women had longer patient and treatment delays (test for interaction between age and sex: p=0.031, and 0.029 respectively).	-	-
Karim<br>2007<br>Bangladesh<br>Unknown	Cross-sectional<br>102<br>49.0%<br>Mean: M 40.6years; F 32.6 years	TB patients	-	-	-	-	-	-	-	The overall stigma index for female TB patients was significantly higher than it was for males. (p<0.05)<br>Females were significantly associated with greater prominence of the stigma indicators “shamed or embarrassed” “others refuse to visit” and willingness to disclose their illness to others”<br>Males were significantly associated with more partner refusal of sex due to TB, and decision to stay away from work, groups.	Women reported more schooling than men (mean = 6.6 years vs 4.3 years; p=0.00)	-	-	-
Kaulagekar<br>2007<br>India<br>Mixed	Cross-sectional<br>3001<br>40.0%<br>-	TB cases (prevalent cases)	-	-	-	-	-	-	-	-	-	Women had significantly less treatment seeking than men in scheduled caste or tribe (p<0.001)<br>For women, higher age and being married was associated with decreased health-seeking	-	-
Kemp<br>2007<br>Malawi<br>Urban	Cross-sectional<br>179<br>49.0%<br>-	New PTB cases	-	-	-	-	-	Patient cost: no significant difference in direct cost (13 USD for men vs 12 USD for women); men had higher opportunity cost.<br>Household cost: women lost more income-earning opportunity because of caretaking responsibilities	-	-	-	-	No significant difference in number of care-seeking episodes (5.4 for women vs 5.0 for men)	-

TableS1. Summary of included studies

First Author<br>Year<br>Country<br>Setting	Type of study<br>Sample size (N)<br>(% female)<br>Age(years)	Study population	Individual-level delay	Provider-/system-level delay	Type of provider-/system-delay	Combined individual-/provider-/system-level delay	Type of combined delay	Individual-level barriers					Provider-/system-level barriers	Combined individual-/provider-/system-level barrier
								Financial	Physical	Stigma	Health literacy	Sociodemographic		
Pehme<br>2007<br>Estonia<br>Mixed	Cross-sectional<br>185<br>31.9%<br>-	New PTB cases	-	There was no significant gender differences in either prolonged (aOR=1.46; CI= 0.72-2.97); P=0.30) or extreme health system delay (aOR=0.95; CI= 0.42-2.18; P= 0.91)	*	-	-	-	-	-	-	-	-	-
Selvam<br>2007<br>India<br>Mixed	Cross-sectional<br>601<br>26.8%<br>Mean=43 years	New SSM+ TB cases	No significant difference in patient delay>28 days (p=0.8)	No significant difference in provider delay>14 days (p=0.8)	#	-	-	-	-	-	-	-	-	-

TableS1. Summary of included studies

First Author<br>Year<br>Country<br>Setting	Type of study<br>Sample size (N)<br>(% female)<br>Age(years)	Study population	Individual-level delay	Provider-/system-level delay	Type of provider-/system-delay	Combined individual-/provider-/system-level delay	Type of combined delay	Individual-level barriers					Provider-/system-level barriers	Combined individual-/provider-/system-level barrier
								Financial	Physical	Stigma	Health literacy	Sociodemographic		
Sherma<br>2007<br>India<br>Mixed	Cross-sectional<br>1008<br>40.6%<br>Mean<br>(SD)=35.3(12.0)	General population	-	-	-	-	-	-	-	-	A similar proportion of males and females thought that TB was an infectious disease (89.0% vs 89.5%, p=0.8). Among those who thought TB was an infectious disease, significantly fewer women than men thought TB could be transmitted through the air by coughing/sneezing (65.3% vs 76.4%, p=0.003); less women knew hospitalization as a measure to prevent TB transmission (21% vs 27.7%, p=0.01); more men knew cough >3 wks is a symptom of TB (p=0.01); more men knew sputum exam as a diagnostic for TB (47.3% vs 40.6% p=0.04); no significant difference in the knowledge of places where treatment was available (M 64.3% vs F 67.0%, p=0.37); no significant difference in the knowledge of treatment duration (p=0.17)<br>As many women as men thought that TB treatment, cough hygiene and use of separate utensils/food consumption would prevent TB transmission (89.5% vs 89.5%, p=0.998; 26.4% vs 26.7%, p=0.9; 10.5% vs 8.2%, p=0.2).	-	-	-

TableS1. Summary of included studies

First Author<br>Year<br>Country<br>Setting	Type of study<br>Sample size (N)<br>(% female)<br>Age(years)	Study population	Individual-level delay	Provider-/system-level delay	Type of provider-/system-delay	Combined individual-/provider-/system-level delay	Type of combined delay	Individual-level barriers					Provider-/system-level barriers	Combined individual-/provider-/system-level barrier
								Financial	Physical	Stigma	Health literacy	Sociodemographic		
Tiwari<br>2007<br>Nepal<br>Armed conflict areas	Cross-sectional<br>180<br>30.6%<br>Median: 12-45 years	TB cases	-	-	-	-	-	-	No significant difference in visiting TB services was found by effect of armed conflict (e.g. curfew, casualties, killings) between male and female was found due to location of DOTS center (p=0.05). More women could not go health center during rainy season (p=0.019).	-	Males had significantly higher rate of literacy than females (P=0.001)	-	No significant difference in waiting time (p=0.699), separate exam room (p=0.637), and frequency of staff advice (p=0.268). No significant difference in satisfaction with TB services between men (89.6%) and women (87.0%, p=0.647).	-
Wondimu<br>2007<br>Ethiopia<br>Mixed	Cross-sectional<br>198<br>56.3%<br>Mean(SD)=33.6(14.1) for men; 29.7(11.8) for women	TB cases	Patient delay was significantly longer in females (aHR 0.63, 95% CI 0.47-0.84, p=0.002)	Female patients had significantly shorter health system delays (aHR 1.51, 95% CI 1.12-2.04, p=0.007)	#	-	-	-	-	-	-	-	-	-
Xu<br>2007<br>China<br>Rural	Cross-sectional<br>1204<br>38.4%<br>Median=40-59 years	Hospitalized TB suspects	No significant difference between males and females in patient delay (M/F aRR 1.09 95% CI 0.96-1.23, p=0.19).	-	-	-	-	-	-	-	-	-	-	-
Yan<br>2007<br>China<br>Rural	Cross-sectional<br>889<br>-<br>-	Community residents and PTB patients	-	In 1 of 4 provinces, system delay higher for women than men (35% vs 25%, p=0.04)	*	-	-	-	-	-	-	-	-	-
Zhang<br>2007<br>China<br>Rural	Cross-sectional<br>614<br>48.9%<br>-	General population	-	-	-	-	-	-	-	-	More men knew how TB was spread (44% vs 36% , p<0.05)	-	-	-

TableS1. Summary of included studies

First Author<br>Year<br>Country<br>Setting	Type of study<br>Sample size (N)<br>(% female)<br>Age(years)	Study population	Individual-level delay	Provider-/system-level delay	Type of provider-/system-delay	Combined individual-/provider-/ system-level delay	Type of combined delay	Individual-level barriers					Provider-/system-level barriers	Combined individual-/provider-/system-level barrier
								Financial	Physical	Stigma	Health literacy	Sociodemographic		
Farah<br>2006<br>Norway<br>Unknown	Cross-sectional<br>83<br>47.0%<br>Median=32	TB cases, including EPTB	Sex was not associated with patient delay	Gender was not associated with health system delay	#	Gender was not associated with total delay in TB treatment	^	-	-	-	-	-	-	-
Fochsen<br>2006<br>India<br>Rural	Cross-sectional<br>644 interviewed for health seeking behavior; 445 with cough and sought care<br>25.8% (of 644) and 26.5% (of 445)<br>53.3% of population with cough between 15-54	TB suspects	-	-	-	-	-	-	-	-	-	-	No significant difference by gender in being provided sputum examination (aOR 1.2, 95% CI 0.5-2.9)	-
Pehme<br>2006<br>Estonia<br>Mixed	Cross-sectional<br>185<br>31.9%<br>-	New PTB cases	Males were more likely to have prolonged and extreme patient delay (M/F aOR 2.12 95% CI 1.06-4.23, p<0.05, and 3.28 95% CI 1.30-8.26, p<0.05).	-	-	-	-	-	-	-	-	-	-	-
Rojpibulstit<br>2006<br>Thailand<br>Mixed	Cross-sectional<br>202<br>22.8%<br>Mean(SD)=43.3(16.2)	SSM+PTB cases	No significant difference in patient delay (F/M aHR 1.00 95% CI 0.68-1.46)	No significant difference in health system delay (F/M aHR 0.86 95% CI 0.59-1.24)	*	-	-	-	-	-	-	-	-	-
Saly<br>2006<br>Cambodia<br>Rural	Cross-sectional<br>308<br>49.7%<br>Mean(SD)=46(15.1)	SSM+ TB cases	-	-	-	No significant difference in total delay	^	-	-	-	-	-	-	
Sarmiento<br>2006<br>USA<br>Urban	Cross-sectional<br>39<br>30.8%<br>Mean(SD)=41.6(14.4)	New TB cases	-	-	-	No significant difference in total delay (M 21.3 wks F 13.4 wks, p=0.497)	$	-	-	-	-	-	-	

TableS1. Summary of included studies

First Author<br>Year<br>Country<br>Setting	Type of study<br>Sample size (N)<br>(% female)<br>Age(years)	Study population	Individual-level delay	Provider-/system-level delay	Type of provider-/system-delay	Combined individual-/provider-/system-level delay	Type of combined delay	Individual-level barriers					Provider-/system-level barriers	Combined individual-/provider-/system-level barrier
								Financial	Physical	Stigma	Health literacy	Sociodemographic		
van der Werf<br>2006<br>Ukraine<br>Urban	Cross-sectional<br>190<br>34.7%<br>Mean: 41.9 years	New PTB cases	No significant difference in patient delay > 30 days (F/M univariate OR 1.22 95% CI 0.66-2.24)	-	-	-	-	-	-	-	-	-	-	-
Wandwalo<br>2006<br>Tanzania<br>Urban	Cross-sectional study<br>268<br>37.3%<br>Mean (SD)=32.6 (10.9)	TB cases	-	-	-	-	-	-	-	-	-	-	Males were more satisfied with community-based DOT than females (OR 4.96, 95% CI 1.38-17.86)	-
Becerra<br>2005<br>Peru<br>Urban	Cross-sectional<br>3547<br>50.0%<br>Mean=25.7	Contact of new TB cases and self-referred TB suspects	-	-	-	-	-	-	-	-	-	-	-	Women were significantly more likely found by active than passive case-finding (aOR 3.9, 95% CI 0.93-23.1, p=0.053)
Cheng<br>2005<br>China<br>Unknown	Cross-sectional<br>190<br>35.7%<br>Mean=48.1 (SD 16.6)	SSM+ PTB cases	The females gender was found to be associated with longer patient delays but this was not statistically significant (HR= 0.95, CI= 0.66-1.39)	Median health system delay was 6 times longer in females than in males and this was statistically significant. (HR= 0.63, CI= 0.43-0.92, p < 0.05).	*	-	-	-	-	-	-	-	-	-

TableS1. Summary of included studies

First Author<br>Year<br>Country<br>Setting	Type of study<br>Sample size (N)<br>(% female)<br>Age(years)	Study population	Individual-level delay	Provider-/system-level delay	Type of provider-/system-delay	Combined individual-/provider-/system-level delay	Type of combined delay	Individual-level barriers					Provider-/system-level barriers	Combined individual-/provider-/system-level barrier
								Financial	Physical	Stigma	Health literacy	Sociodemographic		
Date<br>2005<br>Yemen<br>Rural	Cross-sectional<br>74<br>44.6%<br>-:	SSM+ PTB cases	-	-	-	-	-	-	-	-	27% of men and 73% of women were illiterate (p=0.12) Illiteracy was a predictor of longer diagnostic delay (OR = 2.9, p = 0.046). Literate females were least likely to have diagnostic delays AND treatment not completed (p < 0.001), and illiterate males were most likely to have diagnostic delay AND treatment not completed (OR 6.4, p = 0.008).	-	-	-
Díez<br>2005<br>Spain<br>Mixed	Cross-sectional<br>5184<br>29.7%<br>-	TB cases	-	Women were significantly associated with extreme health system delay (OR 1.26, 95% CI 1.04-1.49), but not with delay longer than 50th percentile (OR 1.12, 95% CI 0.97-1.29)	#	-	-	-	-	-	-	-	-	-
dos Santos<br>2005<br>Brazil<br>Urban	Cross-sectional<br>1105<br>33.0%<br>-	TB cases	-	-	-	Sex was not associated with total delay in TB treatment (OR of delay for women vs men: 1.18, 95% CI 0.91-1.53, p=0.1884)	^	-	-	-	-	-	-	-
Katamba<br>2005<br>Uganda<br>Rural	Cross-sectional<br>307<br>31.6%<br>Mean (SD): DOTS: 35.1(13.4); SAT: 35.2(12.0)	TB patients	-	-	-	-	-	-	-	-	No significant difference in the belief that TB is associated with HIV/AIDS (p=0.694)	-	-	-

TableS1. Summary of included studies

First Author<br>Year<br>Country<br>Setting	Type of study<br>Sample size (N)<br>(% female)<br>Age(years)	Study population	Individual-level delay	Provider-/system-level delay	Type of provider-/system-delay	Combined individual-/provider-/system-level delay	Type of combined delay	Individual-level barriers					Provider-/system-level barriers	Combined individual-/provider-/system-level barrier
								Financial	Physical	Stigma	Health literacy	Sociodemographic		
Kiwuwa<br>2005<br>Uganda<br>Unknown	Cross-sectional<br>231<br>42.9%<br>Mean (SD)=30.7 (9.4)	SSM+ TB cases	No significant difference in patient delay (univariateM/F OR 1.83 95% CI 1.02-3.29)	No significant difference in health service delay (univariateM/F OR 0.95 95% CI 0.49-1.86)	#	-	-	Significantly higher proportion of females had no income (36.4% vs 11.4%, p<0.001)	-	-	-	-	-	-
Lambert<br>2005<br>Bolivia<br>Urban	Cross-sectional<br>144<br>42.0%<br>Median=27 for men; 29 for women	TB cases	-	-	-	Significantly longer total delay to treatment in females (p=0.024)	^	No significant difference by gender in median out-of-pocket expenditure before NTP(USD 17 for men vs USD 11 for women, p=0.11)	-	-	-	-	-	-
Promtussananon<br>2005<br>South Africa<br>Semi-urban	Cross-sectional<br>80<br>50%<br>Equally distributed to four age groups (11-19, 20-34, 35-49, >=50)	General population	-	-	-	-	-	-	-	-	11.3% of women vs 2.5% of men reported a biomedical causes local cultural explanation for tuberculosis (p=0.02). There was no difference between men and women with respect to belief in biomedical vs local cultural explanations for preventing (p=0.05) or treating TB (p=0.34).	-	-	-
Rozovsky-Weinberger<br>2005<br>USA<br>Urban	Cross-sectional<br>937<br>30.5%<br>Mean(SD)=43(14)	TB cases	-	There was higher proportion of delay for AFB for women (F/M aOR 1.66 95 % CI 1.06-2.60, p=0.03); no significant difference in TB isolation (F/M aOR 1.40 95% CI 0.98-1.99, p=0.06)	*	-	-	-	-	-	-	-	-	-

TableS1. Summary of included studies

First Author<br>Year<br>Country<br>Setting	Type of study<br>Sample size (N)<br>(% female)<br>Age(years)	Study population	Individual-level delay	Provider-/system-level delay	Type of provider-/system-delay	Combined individual-/provider-/system-level delay	Type of combined delay	Individual-level barriers					Provider-/system-level barriers	Combined individual-/provider-/system-level barrier
								Financial	Physical	Stigma	Health literacy	Sociodemographic		
Xu<br>2005<br>China<br>Rural	Cross-sectional<br>493<br>27.8%<br>Median: 40-59 years	TB cases	No significant difference in patient delay in two counties between male and female TB cases.	No significant difference in doctor's delay in two counties between male and female TB cases.	*	-	-	-	-	-	-	-	-	-
Yimer<br>2005<br>Ethiopia<br>Rural	Cross-sectional<br>384<br>47.4%<br>Median(range)=28(16-70)	New SSM+ PTB patients	No significant gender differences in patient delay	Provider delay did not vary by gender	#	-	-	-	-	-	-	-	-	-
Ahsan<br>2004<br>Bangladesh<br>Rural	Cross-sectional<br>307<br>52.8%<br>Mean= 36 (range 15-90)	TB cases	A larger proportion of those who delayed for 14-60 days were men. Of those who had > 60 days delay, 50% were women whereas only 29.8% were males.	-	-	-	-	More women than men were unemployed/housewives, which predicted health seeking.<br>More females than males reported difficulties paying for travel to access care (83.3% vs 66.2%, p<0.001).	-	More females than males wanted to be diagnosed secretly (82.7% vs 23.4%, p<0.001).<br>Women were more likely than men to report problems in the relationship with their spouse (72.1% vs 49.2%, p<0.001); to report problems in their relationship with other family members (77.2% vs 36.6%, p<0.001). Men were more likely than women to report difficulty in finding a marriage partner (64.1% vs 45.7%, p=0.001).	-	More women than men were unmarried, which predicted against health seeking.	More women than men were uncomfortable with clinic hours or dissatisfied with provider's behavior/TB services, both of which were predictors of health seeking. Fewer females than males reported satisfaction with clinic hours (69.8% vs 78.6%, p=0.077); satisfaction with providers' behaviors (61.1% vs 75.2%, p=0.009); and satisfaction with TB services (65.4% vs 75.9%, p=0.046).	-

TableS1. Summary of included studies

First Author<br>Year<br>Country<br>Setting	Type of study<br>Sample size (N)<br>(% female)<br>Age(years)	Study population	Individual-level delay	Provider-/system-level delay	Type of provider-/system-delay	Combined individual-/provider-/system-level delay	Type of combined delay	Individual-level barriers					Provider-/system-level barriers	Combined individual-/provider-/system-level barrier
								Financial	Physical	Stigma	Health literacy	Sociodemographic		
Atre[2]<br>2004<br>India<br>Rural	Cross-sectional<br>160<br>50%<br>Mean 39.7	Community members who did not have TB and whose immediate family members did not have TB	-	-	-	-	-	Financial problems, including job loss (P < 0.1) and reduced family income (P = 0.07), were reported more frequently for the male than for the female vignette.	-	There were no statistically significant gender differences in respondents responses to stigma indicators (tested using male and female vignettes)	There were no gender differences in the ability to recognize TB. Significantly more men (74%) than women (15%) identified the mass media as sources of TB information. (P<0.01). More men (31%) than women (14%) identified health services as a source of TB information (p<0.05).	-	-	-
Balasubramanian<br>2004<br>India<br>Rural	Cross-sectional<br>Community:76011<br>TB suspects:8646<br>TB patients:1967	General population, TB suspects, TB cases	No gender difference in delay was found. Median delay was 14 days for both gender (P=0.2)	Men and women experienced similar health system delays regardless of whether they consulted government provider first (median 15 vs 17 days, p=0.6) or private provider first (median 42 vs 45 days, p=0.6).	*	-	-	Women spent significantly more money for care seeking than men (500 vs 300 rupees; P< 0.001). For both men and women, it cost 5 times more to consult a private provider than a government provider (500 vs 100 rupees; P< 0.001)	-	Women faced significantly greater stigma than men in terms of discussing their illness with family and friends (21% vs 14%, p<0.05), feeling unwelcome to participate in social events (18% vs 12%, p<0.05) or facing rejection due to their illness (4% vs 2%, p<0.05).	-		-	Among SSM+ TB patients, women were less likely to be notified (the probability of notification was significantly higher for women than men, 0.54 vs 0.33; p<0.01)

TableS1. Summary of included studies

First Author<br>Year<br>Country<br>Setting	Type of study<br>Sample size (N)<br>(% female)<br>Age(years)	Study population	Individual-level delay	Provider-/system-level delay	Type of provider-/system-delay	Combined individual-/provider-/system-level delay	Type of combined delay	Individual-level barriers					Provider-/system-level barriers	Combined individual-/provider-/system-level barrier
								Financial	Physical	Stigma	Health literacy	Sociodemographic		
Dandona<br>2004<br>India<br>Mixed	Cross-sectional<br>83099 for utilization; 4310 for barriers<br>35.2% for utilization; 40.7% for barriers<br>-	TB cases and suspects	No significant association between gender in not having completing TB diagnostic procedures (female/male aOR 1.17, 95% CI 0.88-1.56); no significant difference in proportions of individuals who did not start TB treatment among those who completed diagnostic process (4.2% men vs 3.4% women, p=0.157)	-	-	-	-	No significant difference in not completing TB diagnostic procedures because of economic reasons by gender (0.5% men vs 4.2% women, p=0.061)	-	-	Women were less likely to not complete TB diagnostic procedures because they thought symptoms improved (15.8% vs 26.3%, p=0.043)	-	No significant difference by gender in health provider-related factors as reasons not starting TB treatment after being diagnosed (37.9% men vs 44.4% women, p=0.892)	-
Díez<br>2004<br>Spain<br>Mixed	Cross-sectional<br>6993<br>32.0%<br>-	TB cases	Females were significantly associated with extreme patient delay (aOR 1.15, 95% CI 1.01-1.30), but not with patient delay longer than 50th percentile (aOR 1.10, 95% CI 0.98-1.22)	-	-	-	-	-	-	-	-	-	-	-

TableS1. Summary of included studies

First Author<br>Year<br>Country<br>Setting	Type of study<br>Sample size (N)<br>(% female)<br>Age(years)	Study population	Individual-level delay	Provider-/system-level delay	Type of provider-/system-delay	Combined individual-/provider-/system-level delay	Type of combined delay	Individual-level barriers					Provider-/system-level barriers	Combined individual-/provider-/system-level barrier
								Financial	Physical	Stigma	Health literacy	Sociodemographic		
Hamid Salim<br>2004<br>Bangladesh<br>Rural	Cross-sectional<br>TB suspects: 7001;<br>TB cases: 64<br>TB suspects: 34.4%;<br>TB cases: 25.0%<br>-	General population	-	-	-	-	-	Significantly more female suspects and cases than male are financially dependent (84% vs 21% among suspects; 88% vs 23% among cases, both p<0.00001.) Significantly more men than women perceived financial barriers as a cause for not seeking treatment (76% vs 68% among suspects who did not seek care, p<0.0001).	No significant difference (small sample size)	No significant difference (small sample size)	Significant fewer female TB suspects than male were aware of TB symptoms (63% vs 69%, p<0.00001); No significant difference in awareness among TB cases; significantly smaller proportion of male suspects cited lack of total awareness as a reason not to seek treatment. (16% vs 19%, p<0.05)	-	-	-
Hoa<br>2004<br>Vietnam<br>Mixed	Cross-sectional<br>364<br>32.7%<br>48.6% between 25-54	TB cases	-	-	-	-	-	-	-	No significant difference in negative attitude toward TB between men and women (49.4% men vs 58.8% women, p=0.09)	No significant difference in overall TB knowledge. (p=0.052), however women were less knowledgeable that TB is transmissible (91.6% vs 97.2%, p=0.02); no significant difference in knowledge of mode of transmission of TB or that TB is curable (p=0.13, 0.94)	-	No significant difference in health education received by gender of patient	
Koay<br>2004<br>Malaysia<br>Unknown	Cross-sectional<br>205<br>49.3%<br>Mean (SD)=36.1 (9.8)	General population	-	-	-	-	-	-	-	No significant gender-related differences regarding whether TB patients should mix socially with others (p=0.9)or are dirty (p=0.2); or embarrassment if infected with TB (p = 0.9).	No significant difference in mean score of knowledge of TB symptoms (p=0.6), mean general knowledge score (p=0.06), or duration of TB treatment (p=0.6)	-	-	-
Odusanya<br>2004<br>Nigeria<br>Unknown	Cross-sectional<br>141<br>37.0%<br>Mean(SD)=29.5(11.0)	New TB cases	No significant difference by gender (p=0.87).	-	-.	-	-	-	-	-	-	-	-	-

TableS1. Summary of included studies

First Author<br>Year<br>Country<br>Setting	Type of study<br>Sample size (N)<br>(% female)<br>Age(years)	Study population	Individual-level delay	Provider-/system-level delay	Type of provider-/system-delay	Combined individual-/provider-/system-level delay	Type of combined delay	Individual-level barriers					Provider-/system-level barriers	Combined individual-/provider-/system-level barrier
								Financial	Physical	Stigma	Health literacy	Sociodemographic		
Paynter<br>2004<br>UK<br>Urban	Cohort<br>71<br>32.4%<br>Mean=38.1	PTB cases	No significant difference in patient delay (median 26 days among men vs 50 days among women p=0.13).	No significant difference in health system delay (median 26.5 days among men vs 41.5 days among women, p=0.42).	#	No significant difference in total delay (median 77.5 days among men vs 93 days among women, p=0.53).	^	-	-	-	-	-	-	-
Shetty<br>2004<br>United Kingdom<br>Urban	Cross-sectional<br>75<br>45.3%<br>-	TB patients; household contact; community residents	-	-	-	-	-	-	-	No significant difference by gender in living and coping with TB and how others related to patients' disease	-	-	-	-
Thorson<br>2004<br>Vietnam<br>Mixed	Cross-sectional<br>35832<br>53.3%<br>-	General population	-	-	-	-	-	No significant difference in the proportion of TB cases in lowest quintile income level by gender (60% vs 67%, p=0.78)	-	-	-	-	-	Among self-referred patients (i.e. passive case-finding), the case detection rates were estimated to be 39% (20-76%) among men and 12%(6-26%) among women. Men had significantly higher case detection rate (RR 3.3 95% CI 1.9-5.8), suggesting women were under-represented by self-referral

TableS1. Summary of included studies

First Author<br>Year<br>Country<br>Setting	Type of study<br>Sample size (N)<br>(% female)<br>Age(years)	Study population	Individual-level delay	Provider-/system-level delay	Type of provider-/system-delay	Combined individual-/provider-/system-level delay	Type of combined delay	Individual-level barriers					Provider-/system-level barriers	Combined individual-/provider-/system-level barrier
								Financial	Physical	Stigma	Health literacy	Sociodemographic		
Agboatwalla<br>2003<br>Pakistan<br>Mixed	Cross-sectional<br>754<br>49.0%<br>-	General population	-	-	-	-	-	-	-	-	Significantly higher overall literacy of men than women both in urban and rural sites (p<0.05); more men than women knew TB is transmitted through droplet in both sites (p=0.02, 0.02); more urban women than men thought TB is transmitted through sexual contact (14.2% vs 3.1%, p<0.05); men had more knowledge TB symptoms (lesser difference in urban areas)	-	-	-
Ailinger<br>2003<br>USA<br>Mixed	Cross-sectional<br>14727<br>14.1%<br>-:	General population	-	-	-	-	-	-	-	-	Gender was slightly associated with knowledge ofperceived risk, 23.8% of men and 25.7% of women answered all questions correctly (p < 0.001).	-	-	-
Harries<br>2003<br>Malawi<br>Mixed	Cross-sectional<br>329<br>53.5%<br>Mean: 36	Hospitalized TB cases	-	-	-	-	-	-	-	-	-	-	No difference in the odds of “exceeding ideal maximal number” of health facility visits by gender for both smear positive (aOR 1.26, p = 0.4) and negative patients (aOR 0.94, p=0.9).	-

TableS1. Summary of included studies

First Author<br>Year<br>Country<br>Setting	Type of study<br>Sample size (N)<br>(% female)<br>Age(years)	Study population	Individual-level delay	Provider-/system-level delay	Type of provider-/system-delay	Combined individual-/provider-/system-level delay	Type of combined delay	Individual-level barriers					Provider-/system-level barriers	Combined individual-/provider-/system-level barrier
								Financial	Physical	Stigma	Health literacy	Sociodemographic		
Hashim<br>2003<br>Iraq<br>Mixed	Cross-sectional<br>500 patients<br>500 HCWs<br>Patient: 33.4%<br>Health care worker: 22.0%<br>Mean (SD) for patient=36.4 (16.4)<br>75%of HCWs were >30 years	Health care workers and SSM+ TB cases	-	-	-	-	-	-	-	-	Among patients, no significant difference in TB knowledge (65.8% vs 61.7%, chi-squared=0.81);	-	Among health care workers, lower proportion of women had good knowledge (96.4% vs 99.0%, non-significant)	-
Hoa<br>2003<br>Vietnam<br>Rural	Cross-sectional<br>559<br>53.7%<br>56% between 25-64	TB suspects	Women significantly had more healthcare action (p<0.001)	-	-	-	-	-	-	-	More women than men were illiterate (15% vs 4%, p<0.001); significantly higher mean knowledge score in men than women (3.04 vs 2.55, p<0.001); more men knew TB is transmitted through droplet (p=0.04); knowledge of TB symptoms was associated with increased healthcare action or seeking hospital care	-	-	-

TableS1. Summary of included studies

First Author<br>Year<br>Country<br>Setting	Type of study<br>Sample size (N)<br>(% female)<br>Age(years)	Study population	Individual-level delay	Provider-/system-level delay	Type of provider-/system-delay	Combined individual-/provider-/system-level delay	Type of combined delay	Individual-level barriers					Provider-/system-level barriers	Combined individual-/provider-/system-level barrier
								Financial	Physical	Stigma	Health literacy	Sociodemographic		
Kamel<br>2003<br>Egypt<br>Urban	Cross-sectional<br>334<br>30.8%<br>Mean (SD)=36.9 (14.6); range: 14-75.	TB cases	-	The delay in starting treatment was significantly longer for males (men 1.4 weeksvs women 1.2 weeks, p<0.001).	@	No significant difference in the delay before diagnosis. (men 2.2 month vs women 2.1 month, p=0.367)	$	-	No significant difference in 1) mean distance to health service (p=0.323) 2) mean commuting time to the chest clinic(p=0.868) 3)means of transport.	No difference in overall perception about social isolation or family relationship (p=0.982, 0.471), however more women did not agree that TB affects marital status (p=0.044) and more men did not agree TB decreases the chance of marriage of young patients (p=0.009)	Knowledge about TB significantly lower in females (p= 0.042); significantly more women missed one or more health education sessions (11.7% vs 5.6%, p= 0.050); no significant difference in knowledge that TB is an infectious disease (p=0.46); more men thought TB leads to complication during pregnancy, TB affects pregnancy, and breastfeeding (p=0.015, 0.026, 0.008); no significant difference by gender in opinions about the relationship between TB and female infertility (p=0.629)	-	No significant difference in satisfaction with overall quality of health care; but men were significantly more satisfied with drug availability (p=0.048).	-
Santha<br>2003<br>India<br>Mixed	Cross-sectional<br>719<br>21.3%<br>-	PTB cases	-	-	-	-	-	-	-	-	-	-	-	Patients detected by community-based active case-finding vs by health facilities were more likely to be male than female (M/F aOR 2.7 95% CI 1.6-4.5, P<0.001)
Sudha<br>2003<br>India<br>Mixed	Cross-sectional<br>455<br>54.1%<br>52.3%>45 years	TB suspects who consulted a healthcare provider	No significant difference in patient delay (p=0.97)	-	-	-	-	-	-	-	-	-	-	-

TableS1. Summary of included studies

First Author<br>Year<br>Country<br>Setting	Type of study<br>Sample size (N)<br>(% female)<br>Age(years)	Study population	Individual-level delay	Provider-/system-level delay	Type of provider-/system-delay	Combined individual-/provider-/system-level delay	Type of combined delay	Individual-level barriers					Provider-/system-level barriers	Combined individual-/provider-/system-level barrier
								Financial	Physical	Stigma	Health literacy	Sociodemographic		
Demissie<br>2002<br>Ethiopia<br>Urban	Cross-sectional<br>700<br>46.9%<br>Mean= 31	PTB cases	SSM+: Females had 0.98 the odds of longer delays than males, but this was not statistically significant (CI 0.61-1.57)<br>SSM-: Females had 1.1 times the odds of longer delays than males but this was not statistically significant(CI= 0.72-1.69).	-	-	-	-	-	-	-	-	-	-	-
Dhingra<br>2002<br>India<br>Urban	Cross-sectional<br>301<br>46.8%<br>-:	PTB cases	No gender difference in delays was found	-	-	-	-	-	-	-	-	-	-	-
Godfrey-Faussett<br>2002<br>Zambia<br>Urban	Cross-sectional<br>427<br>59.5%<br>68% between 20-39	TB suspects	No significant difference in patient delay. (38.2% for men vs 32.7% for women, p=0.29)	-	-	-	-	Sex was not associated with socio-economic factors (p=0.6)	Sex was not associated with distance to clinic (p=0.1).	Sex was significantly associated with stigma (p=0.019, direction was not reported)	Sex was not associated with knowledge of TB (p=0.93), but was associated with education (p=0.00007, direction not reported); poor TB knowledge was associated with increased stigma (p=0.004)	-	-	-
Malhotra<br>2002<br>India<br>Rural	Cross-Sectional<br>213<br>52.1%<br>Mean=43.02; range: 20-90	General population	-	-	-	-	-	-	-	-	Males significantly more than women were aware about symptoms, mode of spread, and etiology of TB	-	-	-
Masjedi<br>2002<br>Iran<br>Urban	Cross-sectional<br>50<br>60%<br>Mean(SD)=42(19)	New SSM+ TB cases	Men had longer patient delays (mean 15.5 vs 10.5 days, p=0.12)	Women had longer physician delays (108 vs 70 days, p<0.05)	*, @	-	-	13% of women delayed seeking medical advice due to dependence on their husbands.	-	-	-		-	-

TableS1. Summary of included studies

First Author<br>Year<br>Country<br>Setting	Type of study<br>Sample size (N)<br>(% female)<br>Age(years)	Study population	Individual-level delay	Provider-/system-level delay	Type of provider-/system-delay	Combined individual-/provider-/system-level delay	Type of combined delay	Individual-level barriers					Provider-/system-level barriers	Combined individual-/provider-/system-level barrier
								Financial	Physical	Stigma	Health literacy	Sociodemographic		
Rajeswari<br>2002<br>India<br>Mixed	Cross-sectional<br>531<br>28.1%<br>Mean(range)=38(15-80)	SSM+ new PTB cases	Men waited somewhat longer than women to seek care (20 vs 15 days. P= 0.07) A significantly higher proportion of women (77%) than men (68%) sought care within 30 days of onset of symptoms.(X2= 4.3, P= 0.0382)	No significant difference in median health system delay (M 23 days vs F 30 days).No significant difference in the proportion of diagnosed > 7 days (M 68% vs F 73% p=0.18)	*	-	-		-	-	-	-	-	-

TableS1. Summary of included studies

First Author<br>Year<br>Country<br>Setting	Type of study<br>Sample size (N)<br>(% female)<br>Age(years)	Study population	Individual-level delay	Provider-/system-level delay	Type of provider-/system-delay	Combined individual-/provider-/system-level delay	Type of combined delay	Individual-level barriers					Provider-/system-level barriers	Combined individual-/provider-/system-level barrier
								Financial	Physical	Stigma	Health literacy	Sociodemographic		
Getahun<br>2001<br>Ethiopia<br>Rural	Cross-sectional<br>1000<br>25.6%<br>Mean= 40.1(SD 10.8)	General population	-	-	-	-	-	-	-	Significantly higher proportion of males than females would allow home visits from TB patients on treatment. (36.4% vs 26.2%; X^2= 8.9; P= 0.002); to continue marriage if spouse develops TB. (70.2% vs 66%; X2= 53.1; P= 0.0001); to shake hands with a TB patient (24.9% vs 13.3%, x^2=14.9 p=0.001); to attend social gathering with TB patients on treatment on the open field (25% vs 19.5% x^2=3.2 p=0.075) Higher but not statistically significant proportion of males than females would allow siblings to play with children from a TB family (9.1% vs 6.2% x^2=2.1 p=0.15); to attend social gathering with TB patient on treatment on the same room (14.6% vs 12.1% x^2=1.0 p=0.31)	A lower proportion of females than males believed TB is curable. (57% vs 70%; X^2= 14.5; P= 0.001)	-	-	-
Lienhardt<br>2001<br>The Gambia<br>Mixed	Cross-sectional<br>152<br>27.6%<br>Mean=36 for men and 30 for women	TB cases	-	-	-	No significant difference by gender in total delay (aHR of delay among men vs women 0.74, 95% CI 0.34-1.62, p=0.46)	^	-	-	-	-	-	Women had seen a significantly higher number of healthcare providers (p=0.01)	-

TableS1. Summary of included studies

First Author<br>Year<br>Country<br>Setting	Type of study<br>Sample size (N)<br>(% female)<br>Age(years)	Study population	Individual-level delay	Provider-/system-level delay	Type of provider-/system-delay	Combined individual-/provider-/system-level delay	Type of combined delay	Individual-level barriers					Provider-/system-level barriers	Combined individual-/provider-/system-level barrier
								Financial	Physical	Stigma	Health literacy	Sociodemographic		
Needham<br>2001<br>Zambia<br>Urban	Cross-sectional<br>202<br>44.0%<br>53% <32	TB cases	-	-	-	Delay from symptom onset to initiation of treatment was significantly longer in women than men (72 days vs 58 days, p=0.02)	^	-	-	-	-	-	-	-
Ngamvithayapong<br>2001<br>Thailand<br>Unknown	Cross-sectional<br>557<br>33.7%<br>Median=37	New SSM+ TB cases	Women had longer patient delay (aOR=1.04, 95%CI 0.69-1.58)	Women had longer provider delay (univariate OR=1.43, 95%CI 1.01-2.04)	*	-	-	Significantly higher proportion of women than men were unemployed/had no income (37.2% vs 26.5% p=0.03)	-	-	No significant difference by gender in proportion of being illiterate(M 21.7% vs F 28.7% , p=0.10)	-	-	-
Pronyk<br>2001<br>South Africa<br>Rural	Cross-sectional<br>298<br>40.6%<br>Mean(SD)=40.4(15.5)	Hospitalized PTB cases	No significant difference in patient delay (F/M HR 1.00 95% CI 0.78-1.26)	Women had longer service provider delay (F/M HR 0.64 95% CI 0.49-0.83, p<0.05)	*	-	-	-	-	-	-	-	-	-
Sadiq<br>2001<br>Pakistan<br>Mixed	Cross-sectional<br>160<br>47.0%<br>Mean(SD)=33(16.6)	New registered TB cases	No significant difference in patient delay (p=0.19)	-	-	-	-	No significant difference in mean travel cost (p=0.45)	No significant difference in travel time and distance from residence to TB center (p=0.44, 0.58, respectively)	-	-	-	-	-
Ward<br>2001<br>Australia<br>Mixed	Cross-sectional<br>781<br>42.4%-42.6%<br>Mean= 54.8 years among men; 46.1 among women	TB cases	All TB: No significant difference in patient delay (M/F aRR 0.93 95% CI 0.80-1.08).<br>SSM+ TB: OR 1.02 (0.81-1.27)	All TB: Men had shorter system delay (M/F aRR 0.78 95% CI 0.68-0.91)<br><br>SSM+ TB: OR 0.78 (0.63-0.97)	#	-	-	-	-	-	-	Stratification of sex by age dichotomized at 45 years and risk group showed that That the principal source of age-corrected difference between females and males for health care system delays was in the non-indigenous Australian-born group. Females aged 45 and over in this group had significantly longer times to diagnosis (P<0.01).	-	-

TableS1. Summary of included studies

First Author<br>Year<br>Country<br>Setting	Type of study<br>Sample size (N)<br>(% female)<br>Age(years)	Study population	Individual-level delay	Provider-/system-level delay	Type of provider-/system-delay	Combined individual-/provider-/system-level delay	Type of combined delay	Individual-level barriers					Provider-/system-level barriers	Combined individual-/provider-/system-level barrier
								Financial	Physical	Stigma	Health literacy	Sociodemographic		
Yamasaki-Nakagawa<br>2001<br>Nepal<br>Rural	Cross-sectional<br>336<br>29.0%<br>Median=37 for men and 30 for women	TB cases: SSM+, SSM-, EPTB	No significant difference in patient delay by gender (0.8 month for men vs 0.6 month for women, p=0.7);	Women had slightly more health provider delay than men (1.3 month vs 0.8 month, p=0.054)	*	Women had longer total delay than men (median 3.3 months vs. 2.3 months p=0.034)	$	No significant difference by gender in fee paid to health care providers before TB diagnosis (p=0.36)	No significant difference by gender in travelling time to governmental medical establishment where patients got treated (p=0.35)	-	-	-	-	-
Thorson<br>2000<br>Vietnam<br>Unknown	Cross-sectional<br>492<br>56.7%<br>-	TB suspects from population survey	Being a woman was associated with longer delay to first hospital visit	-	-	-	-	Male is significantly associated with a higher total cost per health care action (p=0.01)	-	-	-	-	Significantly more men than women reported giving a sputum sample at hospital (36% vs 14%, p=0.0006); women were associated with increased number of care-seeking actions	-
Wandwalo[5]<br>2000<br>Tanzania<br>Mixed	Cross-sectional<br>296<br>33.4%<br>Mean (SD)= 36.2 (12)	SSM+ TB cases	No significant difference in patient delay (p=0.854); no significant different in patient delay longer than 30 days (p=0.469)	-	-	There were no significant gender differences in delays.	^	-	-	-	-	-	-	-
Wandwalo[5]<br>2000<br>Tanzania<br>Mixed	Cross-sectional<br>296<br>33.4%<br>Mean (SD)=36.2 (12)	New SSM+ PTB cases	-	-	-	-	-	-	-	-	No significant difference about knowledge of TB symptom/sign (p=0.398),knowledge of cause and transmission (p=0.889), and overall satisfactory knowledge of TB (F/M aOR 1.64 95% CI 0.89-3.01, p=0.114).	-	-	-

TableS1. Summary of included studies

First Author<br>Year<br>Country<br>Setting	Type of study<br>Sample size (N)<br>(% female)<br>Age(years)	Study population	Individual-level delay	Provider-/system-level delay	Type of provider-/system-delay	Combined individual-/provider-/system-level delay	Type of combined delay	Individual-level barriers					Provider-/system-level barriers	Combined individual-/provider-/system-level barrier
								Financial	Physical	Stigma	Health literacy	Sociodemographic		
Long<br>1999<br>Vietnam<br>Mixed	Cross-sectional<br>1027<br>26.0%<br>Mean(SD)=34.2(7.8) for men and 32.7(8) for women; range =15-49	TB cases	No significant difference by gender in patient delay (7.6 weeks among men vs 7.9 weeks among women, p=0.71); no significant difference by gender in delay to first health care provider (5.9 weeks for men vs 5.6 weeks for women, p=0.65);	Women had significantly longer qualified doctor's delay (5.4 weeks vs 3.8 weeks, p=0.003), and health care provider delay (7.7 weeks vs 5.5 weeks, p=0.003)	*	Total delay was significantly longer in females (13.3 weeks vs 11.4 weeks, p=0.02).	-	-	Area of residence did not show differential impact on delay in TB diagnosis	-	Men had higher mean level of education (p=0.011); women with higher education attended the hospital significantly more often than women with less education.	Family size did not show differential impact on delay in TB diagnosis	-	-
Rajeswari<br>1999<br>India<br>Mixed	Cross sectional<br>304<br>39.5%<br>Mean (SD)= 37.8(14.9)	New SSM+ TB patients	-	-	-	-	-	The average number of work days lost was 83 days for females and 85 days for males.<br>The direct cost incurred by female patients was Rs 2287 compared to Rs 1899 for males	-	More than 2/3rds of females avoided discussing their illness with neighbors	No significant difference by gender in being illiterate (M: 25.0% vs W 34.2% p=0.14)	-	-	-
Sherman<br>1999<br>USA<br>Urban	Cross-sectional<br>184<br>37.0%<br>Median (range)=40 (15-95)	TB cases without prior history of TB treatment	-	No significant difference in system delay	#	-	-	-	-	-	-	-	-	-
Tulsky<br>1999<br>USA<br>Urban	Cross-sectional<br>292<br>29.5%<br>Mean=40	Homeless shelter residents	-	-	-	-	-	-	-	-	Homeless men and women showed no difference in perceived TB susceptibility.	-	-	-
Asch<br>1998<br>USA<br>Unknown	Cross-sectional<br>248<br>36%<br>Age <65y (82%)	TB cases	No significant gender differences was found	-	-	-	-	-	-	-	-	-	-	-

TableS1. Summary of included studies

First Author<br>Year<br>Country<br>Setting	Type of study<br>Sample size (N)<br>(% female)<br>Age(years)	Study population	Individual-level delay	Provider-/system-level delay	Type of provider-/system-delay	Combined individual-/provider-/ system-level delay	Type of combined delay	Individual-level barriers					Provider-/system-level barriers	Combined individual-/provider-/system-level barrier
								Financial	Physical	Stigma	Health literacy	Sociodemographic		
Lawn<br>1998<br>Ghana<br>Mixed	Cross-sectional<br>100<br>40.0%<br>Mean(SD)=36.8(14.7)	TB cases	No significant difference by gender in patient delay	Doctor delay was significantly longer for female patients (p<0.001)	*	No significant difference by gender in total delay, although women seemed to have longer total delay.	-	-	-	-	-	-	-	-
Marinac<br>1998<br>US<br>Urban	Cross-sectional<br>505<br>67.7%<br>Median: 31-40	Non-Caucasians	-	-	-	-	-	-	-	-	Self- assessed knowledge of TB was greater in women than in men (44% vs 33%); men had higher overall correct responses to objective TB knowledge questions. (63% vs 60%, p=0.00311); more men knew children were at higher risk for serious TB (55% vs 40%, p=0.00235); more men knew 10-15 million people in US are infected with TB (69% vs 53, p=0.011)	-	-	-
Enkhbat<br>1997<br>Mongolia<br>Unknown	Cross-sectional<br>107<br>45.8%<br>Mean= 33	PTB cases	Compared to females, males had longer delays. This difference was not statistically significant. (OR= 1.07, C!= 0.71-1.61)	No gender difference in delays was found	*	Compared to females males had shorter total delays. This difference was not statistically significant. (OR= 0.92, CI=0.60-1.40)	^	-	-	-	-	-	-	-
Hooi<br>1994<br>Malaysia<br>Unknown	Cross-sectional<br>100<br>26%<br>Median: 45 for men; 56 for women	New TB cases	Fewer men than women presented to a medical facility within 1 month (56.8% vs 92.3%, p<0.01)	No significant difference in doctor's delay	#	-	-	-	-	-	-	-	-	-

TableS1. Summary of included studies

First Author<br>Year<br>Country<br>Setting	Type of study<br>Sample size (N)<br>(% female)<br>Age(years)	Study population	Individual-level delay	Provider-/system-level delay	Type of provider-/system-delay	Combined individual-/provider-/system-level delay	Type of combined delay	Individual-level barriers					Provider-/system-level barriers	Combined individual-/provider-/system-level barrier
								Financial	Physical	Stigma	Health literacy	Sociodemographic		
Mori<br>1992<br>South Korea<br>Mixed	Cross-sectional<br>630<br>30.0%<br>Mean=40.8	Newly registered TB cases	No gender-related differences (assessed by cumulative proportion)	No difference in doctor's delay	*	No difference in total delay	$	-	-	-	-	-	-	-
Westaway<br>1989<br>South Africa<br>Urban	Cross-sectional<br>211<br>52.6%<br>Mean (SD)= 31.4(12.0)	Primary Health Care clinic or STD clinic patients who were not TB cases or suspects	-	-	-	-	-	-	-	Men tended to feel more threatened by TB than women (p < 0.075).	No significant difference in knowledge scores for TB signs and symptoms; more women thought TB patients should be isolated (58% vs 35%, p<0.01)	-	-	-
Jenkins<br>1977<br>Canada<br>Mixed	Cross-sectional<br>190<br>64.7%<br>Median: 31-40 years	Indian general population	-	-	-	-	-	-	-	-	No significant difference in mean TB knowledge (p=0.34)	-	-	-
CASE-CONTROL STUDY														
Crampin<br>2004<br>Malawi<br>Unknown	Case-control<br>1590<br>47.0%<br>-	TB cases and its control	-	-	-	No significant difference by gender in delay from symptom onset to initiation of treatment	^	-	-	-	In half of patients, a health professional was the first to suspect a diagnosis of tuberculosis, and the pattern was the same for both men and women	-	-	-
COHORT STUDY														

TableS1. Summary of included studies

First Author<br>Year<br>Country<br>Setting	Type of study<br>Sample size (N)<br>(% female)<br>Age(years)	Study population	Individual-level delay	Provider-/system-level delay	Type of provider-/system-delay	Combined individual-/provider-/system-level delay	Type of combined delay	Individual-level barriers					Provider-/system-level barriers	Combined individual-/provider-/system-level barrier
								Financial	Physical	Stigma	Health literacy	Sociodemographic		
Jiménez-Corona<br>2006<br>Mexico<br>Mixed	Cohort<br>623<br>41.1%<br>Mean(SD)=<br>44.3(17.8)	TB cases	-	No significant difference in the interval between diagnosis and initiation of treatment (men: 0.20vs women: 0.02, p=0.22)	@	No significant difference between initiation of symptoms and diagnosis in months (men: 2.90 vs women 2.78, p=0.46), and between initiation of symptoms and treatment (men 3.5o vs women 3.25, p=0.18)	^, $	-	No significant difference in distance to nearest health centre (687 meters for men vs 705 meters for women, p=0.24).	-	-	-	-	-
Rajeswari<br>2005<br>India<br>Unknown	Cohort<br>610<br>33.7%<br>54% males and 57% females<=45	TB cases	-	-	-	-	-	-	-	-	Women had lower literacy (52% vs 70%, p=0.0001); no overall significant difference in knowledge of TB etiology and mode of transmission (p=0.106, 0.194); no significant difference in correct recognition of infection as TB etiology (12% of men vs 16% of women, p>0.05)	-	-	-

TableS1. Summary of included studies

First Author<br>Year<br>Country<br>Setting	Type of study<br>Sample size (N)<br>(% female)<br>Age(years)	Study population	Individual-level delay	Provider-/system-level delay	Type of provider-/system-delay	Combined individual-/provider-/system-level delay	Type of combined delay	Individual-level barriers					Provider-/system-level barriers	Combined individual-/provider-/system-level barrier
								Financial	Physical	Stigma	Health literacy	Sociodemographic		
Bashour<br>2003<br>Syria<br>Mixed	Cohort<br>552<br>33.7%<br>14.9% <20; 44.9% between 20-29	TB cases	Men had significantly longer delay in seeking care than women (mean: men 63.6 days vs women 40.4 days)	-	-	-	-	-	-	Women were significantly more stigmatized (mean perception score: women 5.0/6.0 vs men 3.7/6.0, p<0.001)	No significant difference in each item of TB-related knowledge between both genders (mean knowledge score: men 2.8/4.0 vs women 2.7/4.0, p=0.35)	-	Mean satisfaction score was higher among men (2.8+/-0.4) than women (2.7+/-0.5), p=0.02; more men thought visiting TB clinic was convenient (93.6% vs 87.4%, p>0.05); more women had longer waiting time (12.4% vs 10.9%, p>0.05)	-
Box<br>2003<br>USA<br>Urban	Cohort<br>214<br>38.8%<br>Mean(SD)=36.3(8.9) for men; 32.0(7.3) for women	HIV infected population	-	-	-	-	-	-	-	-	-	-	HIV-infected women and men were screened for TB similarly (81% women vs 77% men, 95% CI -0.07-0.15)	-
Long<br>2002<br>Vietnam<br>Mixed	Cohort<br>1027<br>26.0%<br>Mean=32.7 for women and 34.2 for men; range15-49	TB cases	-	Women had longer doctor's delay than men (1.19 weeks, 95% CI 0.12-2.27); women presented with significantly less cough and expectoration, thus might significantly increase doctor's delay	*	-	-	-	-	-	-	-	-	-

TableS1. Summary of included studies

First Author<br>Year<br>Country<br>Setting	Type of study<br>Sample size (N)<br>(% female)<br>Age(years)	Study population	Individual-level delay	Provider-/system-level delay	Type of provider-/system-delay	Combined individual-/provider-/system-level delay	Type of combined delay	Individual-level barriers					Provider-/system-level barriers	Combined individual-/provider-/system-level barrier
								Financial	Physical	Stigma	Health literacy	Sociodemographic		
Creek<br>2000<br>Botswana<br>Urban	Cohort<br>127<br>40.2%<br>-:	SSM+ PTB TB cases	-	Males had a non-statistically significantly greater odds of delay or failure to start TB treatment after diagnosis (OR = 1.1, p = 0.8).	@	-	-	-	-	-	-	-	-	-
Liam<br>1999<br>Malaysia<br>Urban	Cohort<br>135<br>41.5%<br>Mean(SD)=41.9 (17.4); range 15–84	TB cases	-	-	-	-	-	-	-	-	No significant difference in knowledge score (men 3.19/6 vs women 3.32/6, p=0.676)	-	-	-
Cassels<br>1982<br>Nepal<br>Rural	Cohort<br>270<br>35.2%<br>Mean(SD)= 46.6(13.7) by active finding; 37.9(13.0) passive finding	General population and self-referred new SSM+ TB patients	-	-	-	-	-	-	-	-	-	-	-	Women aged 45 or more were found significantly more by ACF than PCF (27.9% vs 5.0%, p<0.001)
RANDOMIZED TRIAL														

TableS1. Summary of included studies

First Author<br>Year<br>Country<br>Setting	Type of study<br>Sample size (N)<br>(% female)<br>Age(years)	Study population	Individual-level delay	Provider-/system-level delay	Type of provider-/system-delay	Combined individual-/provider-/system-level delay	Type of combined delay	Individual-level barriers					Provider-/system-level barriers	Combined individual-/provider-/system-level barrier
								Financial	Physical	Stigma	Health literacy	Sociodemographic		
Datiko<br>2009<br>Ethiopia<br>Rural	Cluster randomized trial<br>296811<br>47.9%<br>-	General population and self-referred TB suspects	-	-	-	-	-	-	-	-	-	-	-	Mean case detection rate was higher in intervention kebeles (122.2% vs 69.4%, p<0.001) and for female patients (149% vs 91.6%, p<0.001); the improvement of case detection rate was significantly more for women than for men
Khan<br>2007<br>Pakistan<br>Urban	Pragmatic randomized clinical trial<br>3055<br>48.9%	TB suspects referred by physicians or by the NTP	-	-	-	-	-	-	-	-	Brief instruction significantly increased sputum smear positive rate among women, increased a second early morning sputum specimen submission and decreased rate of saliva seen in submitted samples. The significant differences were not seen among men	-	-	-

Legend& Footnote:

-: Not reported; **M**: male; **F**: female; **SD**: standard deviation; **CI**: confidence interval; **TB**: tuberculosis; **PTB**: pulmonary tuberculosis; **EPTB**: extrapulmonary tuberculosis; **SSM+**: sputum smear positive; **SSM-**: sputum smear negative; **HIV**: human immunodeficiency virus; **AIDS**: acquired immunodeficiency syndrome; **OR**: odds ratio; **aOR**: adjusted odds ratio; **HR**: hazard ratio; **aHR**: adjusted hazard ratio; **RR**: risk ratio; **aRR**: adjusted risk ratio; **ACF**: active case-finding; **PCF**: passive case-finding; **DOTS**: directly observed treatment short; **SAT**: self-administered treatment; **STD**: sexually transmitted diseases; **NTP**: national tuberculosis program; **Patient delay**: delay between symptom onset and presentation to health care; **Three types of provider/system-level delay:***: delay between presentation to ANY health care provider and diagnosis (health care provider includes but is not restricted to qualified specialist, primary physician, private practitioner, and traditional healer); **#**: delay between presentation to ANY health care provider and treatment (health care provider includes but is not restricted to qualified specialist, primary physician, private practitioner, and traditional healer); **@**: delay between diagnosis and treatment; **Two types of combined delay:^**: delay between symptom onset and treatment initiation; **$**: delay between symptom onset and diagnosis

[1]Mesfin 2010 and Mesfin 2009 used same population;

[2]Atre 2009 and Atre 2004 used same population;

[3] Both studies by Yimer in 2009 used same population;

[4]Gosoniu 2008, Somma 2008, and Weiss 2008 used same population in Bangladesh, India, and Malawi

TableS1. Summary of included studies

[5]Both studies by Wandwalo in 2000 used same population
