# Supplementary material for: Barriers and Delays in Tuberculosis Diagnosis and Treatment Services: Does Gender Matter?
Source: Tuberc Res Treat. 2014 Apr 28;2014:461935. doi: 10.1155/2014/461935 (PMC4020203; doi:10.1155/2014/461935)
Supplement: Supplementary file 1 — Supplementary Material contains six data tables, which provide detailed summaries of various aspects of the included studies. Tables S1, S2 and S3 present individual study characteristics, the results of the quality assessment and individual study methodologies, respectively. Tables S4, S5, and S6 summarize WHO regional distribution, study population and degree of urbanization, respectively, by outcome type. [file 461935.f1.zip › Table S2.pdf]

TableS2.Quality assessment of included studies

Author	Year	STROBE check list item																									
		4	5	6a	6b	7	8	9	10	11	12a	12b	12c	12d	12e	13a	13b	13c	14a	14b	14c	15	16a	16b	16c	17	22
**Cross-sectional study**																											
Cramm	2010																										
Deribew	2010																										
Dhingra	2010																										
Mesfin	2010																										
Mushtaq	2010																										
Pungrassami	2010																										
Vassall	2010																										
Atre	2009																										
Basnet	2009																										
Berisha	2009																										
Ford	2009																										
French	2009																										
Gele	2009																										
Hoa	2009																										
Lu	2009																										
Mesfin	2009																										
Ngadaya	2009																										
Yimer	2009																										
Yimer	2009																										
Al-Maniri	2008																										
Armijos	2008																										
Aspler	2008																										
Ayuo	2008																										
Bassili	2008																										
Brassard	2008																										
Chimbanrai	2008																										
Gosoniu	2008																										
Kilale	2008																										

TableS2.Quality assessment of included studies

Author	Year	STROBE check list item																									
		4	5	6a	6b	7	8	9	10	11	12a	12b	12c	12d	12e	13a	13b	13c	14a	14b	14c	15	16a	16b	16c	17	22
Lin	2008																										
Lorent	2008																										
Maamari	2008																										
Mahendradhata	2008																										
Marks	2008																										
Meintjes	2008																										
Mfinanga	2008																										
Qureshi	2008																										
Rumman	2008																										
Somma	2008																										
Thierfelder	2008																										
Thongraung	2008																										
Wang, J.M.	2008																										
Wang, Y.	2008																										
Weiss	2008																										
Chang	2007																										
Huong	2007																										
Karim	2007																										
Karim	2007																										
Kaulagekar	2007																										
Kemp	2007																										
Pehme	2007																										
Selvam	2007																										
Sharma	2007																										
Tiwari	2007																										
Wondimu	2007																										
Xu	2007																										
Yan	2007																										
Zhang	2007																										

TableS2.Quality assessment of included studies

Author	Year	STROBE check list item																									
		4	5	6a	6b	7	8	9	10	11	12a	12b	12c	12d	12e	13a	13b	13c	14a	14b	14c	15	16a	16b	16c	17	22
Farah	2006																										
Fochsen	2006																										
Pehme	2006																										
Rojpibulstit	2006																										
Saly	2006																										
Sarmiento	2006																										
van der Werf	2006																										
Wandwalo	2006																										
Becerra	2005																										
Cheng	2005																										
Date	2005																										
Diez	2005																										
dos Santos	2005																										
Katamba	2005																										
Kiwuwa	2005																										
Lambert	2005																										
Promtussananon	2005																										
Rozovsky-Weinberger	2005																										
Xu	2005																										
Yimer	2005																										
Ahsan	2004																										
Atre	2004																										
Balasubramanian	2004																										
Dandona	2004																										
Diez	2004																										
Hamid Salim	2004																										
Hoa	2004																										
Koay	2004																										
Odusanya	2004																										

TableS2.Quality assessment of included studies

Author	Year	STROBE check list item																									
		4	5	6a	6b	7	8	9	10	11	12a	12b	12c	12d	12e	13a	13b	13c	14a	14b	14c	15	16a	16b	16c	17	22
Paynter	2004																										
Shetty	2004																										
Thorson	2004																										
Agboatwalla	2003																										
Ailinger	2003																										
Harries	2003																										
Hashim	2003																										
Hoa	2003																										
Kamel	2003																										
Santha	2003																										
Sudha	2003																										
Demissie	2002																										
Dhingra	2002																										
Godfrey-Faussett	2002																										
Malhotra	2002																										
Masjedi	2002																										
Rajeswari	2002																										
Getahun	2001																										
Lienhardt	2001																										
Needham	2001																										
Ngamvithayapong	2001																										
Pronyk	2001																										
Sadiq	2001																										
Ward	2001																										
Yamasaki-Nakagawa	2001																										
Thorson	2000																										
Wandwalo	2000																										
Wandwalo	2000																										
Long	1999																										

TableS2.Quality assessment of included studies

Author	Year	STROBE check list item																									
		4	5	6a	6b	7	8	9	10	11	12a	12b	12c	12d	12e	13a	13b	13c	14a	14b	14c	15	16a	16b	16c	17	22
Rajeswari	1999																										
Sherman	1999																										
Tulsky	1999																										
Asch	1998																										
Lawn	1998																										
Marinac	1998																										
Enkhbat	1997																										
Hooi	1994																										
Mori	1992																										
Westaway	1989																										
Jenkins	1977																										
**Case-control study**																											
Crampin	2004																										
**Cohort study**																											
Jimenez-Corona	2006																										
Rajeswari	2005																										
Bashour	2003																										
Box	2003																										
Long	2002																										
Creek	2000																										
Liam	1999																										
Cassels	1982																										

**Cluster randomized trial**																		
Author	Year	CONSORT checklist item (for clustered randomized trial)																
		3	4	5	6	7	8	9	10	11	12	13	14	15	16	17	18	19
Datiko	2009																	
**Pragmatic randomized clinical trial**		CONSORT checklist item (for pragmatic trial)																
		3	4	5	6	7	8	9	10	11	12	13	14	15	16	17	18	19
Khan	2007																	

TableS2.Quality assessment of included studies

Legend:

■	criteria met;
(grey)	criteria not met/unclear/not reported;
□	criteria not applicable

STROBE checklist: Vandenbroucke JP, von Elm E, Altman DG, Gotzsche PC, Mulrow CD, et al. (2007) Strengthening the Reporting of Observational Studies in Epidemiology (STROBE): explanation and elaboration. PLoS Med 4: e297.
CONSORT checklist for clustered randomized trial: Campbell MK, Elbourne DR, Altman DG (2004) CONSORT statement: extension to cluster randomised trials. BMJ 328: 702-708.
CONSORT checklist for pragmatic randomized clinical trial: Zwarenstein M, Treweek S, Gagnier JJ, Altman DG, Tunis S et al. (2008) Improving the reporting of pragmatic trials: an extension of the CONSORT statement. BMJ 337:a2390. doi: 10.1136/bmj.a2390
