# Supplementary material for: Barriers and Delays in Tuberculosis Diagnosis and Treatment Services: Does Gender Matter?
Source: Tuberc Res Treat. 2014 Apr 28;2014:461935. doi: 10.1155/2014/461935 (PMC4020203; doi:10.1155/2014/461935)
Supplement: Supplementary file 1 — Supplementary Material contains six data tables, which provide detailed summaries of various aspects of the included studies. Tables S1, S2 and S3 present individual study characteristics, the results of the quality assessment and individual study methodologies, respectively. Tables S4, S5, and S6 summarize WHO regional distribution, study population and degree of urbanization, respectively, by outcome type. [file 461935.f1.zip › Table S3.pdf]

Table S3.Methodological summary of included studies

First Author Year	Inclusion criteria	Selection of study population	Data collection	Measurement of outcome	Assessment of disease
CROSS-SECTIONAL STUDY					
Cramm 2010	Adults over the age of 18 years with 6 months or more of residence in Rhini in the past year	Selection of household was not random; and significantly higher percentage of women were sampled from the selected households (73% among sample vs 56% among households, p<0.0001)	Questionnaire was administered by an experience organization	Knowledge score and self-report of perception of TB and health-seeking behavior	NA
Deribew 2010	Adults older than 14 years of age who lived in the 10 kebeles of the GGFRA	Households were randomly selected in proportion to Kebele size; one adult was randomly sampled from each household; 13% originally selected individuals declined interview, 70% of whom were males	Trained staff with pre-tested questionnaire	Knowledge and prejudice scores toward HIV and TB; individuals were characterized to high prejudice/knowledge vs low based on mean cumulative values of each score	NA
Dhingra 2010	Patients diagnosed and registered under RNTCP for treatment between March and May 2009 in the study chest clinics	Only studied those who were already registered; the representation is basically the slum population	Trained staff with pre-tested questionnaire during intensive phase of treatment	Self-report to questions of TB stigma and TB-related experiences	NA
Mesfin 2010	Newly diagnosed pulmonary TB patients, ≥15 years of age, from the study districts at the time of diagnosis, and suspected cases referred to the diagnostic centers for diagnosis, from January 12, 2005 to January 12, 2006, in Tigray Region, northern Ethiopia.	To improve the validity of our findings, we increased sample size by a factor of 2.4 to allow for drop-out and to permit reliable estimates for gender and other potential risk factors. 10/36 districts were randomly selected. All TB patients and suspected cases were eligible.	Interviewed using a pre-tested questionnaire translated into local language. Direct medical costs were quantified from medical records. Costs of health services were determinedfrom the standard fees instituted by the Tigray Regional Health Bureau and Social Affairs Bureau.	Self-report, medical records, Tigray Regional Health Bureau and Social Affairs Bureau	Sputum microscopy, X-ray
Mushtaq 2010	Respondents aged >=20 years in two districts of Punjab province from January 2008 to January 2009. Health care providers, respondents with a previous history of TB or receiving TB treatment were excluded.	2/35 districts were randomly selected. 3 sub-districts were randomly selected by multistage cluster sampling. 5 union councils (2 urban, 3 rural) were randomly selected. 2 villages or electoral wards were selected from each union council, and 18 individuals were interviewed from each village. Households were selected as follows: at the center of the village, interviewers span a bottle and continued in the direction indicated.	A semi-structured questionnaire designed according to WHO guidelines and translated into the national language, Urdu. The questionnaire was pre-tested and modified accordingly. Each questionnaire was filled in during the household visits by a medical officer trained in interviewing techniques.	Self-report	Knowledge score was obtained by rating nine questions
Pungrassami 2010	Adults (>17 yrs) with newly diagnosed TB between August 2005 and July 2006; excluding those had been receiving TB treatment for more than one month	Only selected those who sought care; asymptomatic patients (3%) were excluded and 7% patients were excluded because of improbable delay time. Excluded patients were similar to those included, except for mode of transportation.	Trained interviewers using a standardized questionnaire	Self-report	Assessment of TB was not reported
Vassall 2010	All patients aged ⩾15 years, using different TB-HIV services at different points in their treatment that consented were eligible for the study. Patients who had completed treatment or who were critically ill were excluded from the study.	Stratified sampling was used to ensure a mix of patients using different TB-HIV services at different points in their treatment. Patients within each quota were interviewed at the facility after receiving treatment.	Structured questionnaire conducted by medical trained interviewers, not directly involved in treating the patient.	Self-report, medical records (registries and patient charts). Indirect costs were estimated using questions based on the living standards measurement surveys	Smear sputum microscopy
Atre 2009	Individuals whose immediate family members (including self) don't have current or previous TB infection.	Equal number by gender and age distribution.	Trained staff with pre-tested questionnaire	Self-report; at risk of misclassification on coding responses (not sure as no)	NA
Basnet 2009	New TB patients at 55 facilities providing DOTS services in Banke district, during June to July 2007.	Selected consecutively during their treatment period from the 55 DOTS centers/sub centers in the district.Retreatment cases were not studied.	Closed-ended questionnaire to tuberculosis patients coming to collect their drugs at any stage of their treatment under National TB Program, by a trained staff member of DOTS treatment centers.	Self-report	Sputum smear, clinical diagnosis, radiographic anomalities
Berisha 2009	TB patients under treatment at the Pulmonary Clinic in the University Clinical Center and anti-TB services in Kosovo.	All eligible patients. 33 hospitalized patients in the Pulmonary Clinic in the University Clinical Center, and 122 patients being treated in the anti-TB services in Kosovo.	Opinion poll carried out by the National Public Health Institute staff and the employees of other regional anti-TB outpatient services directly with the patient and their companions.	Self-report	NA

Table S3.Methodological summary of included studies

First Author Year	Inclusion criteria	Selection of study population	Data collection	Measurement of outcome	Assessment of disease
Ford 2009	Patients 14 years of age or older diagnosed with pulmonary tuberculosis and began treatment with the national tuberculosis control program in the recruitment area over a 4-month period.	Recruitment area included treatment clinics at the Hospital de Apoyo in the center of Iquitos, health posts in the urban shantytown of Belen, and the peri-urban city districts of San Antonio and San Juan. (How?)	Quantitative survey, piloted with 25 patients and then modified based on participants' responses and feedback. Final survey instruments were carried out as face-to-face interviews (by Carolyn Ford). Patients interviewed at the health facility or in their homes, according to their preference.	Self-report	NA
French 2009	Culture-confirmed disease due to Mycobacterium TB complex or other cases: (a) a clinician's judgment that the patient's clinical or radiological signs are compatible with TB and (b) the clinician's decision to treat the patient with a full course of anti-TB treatment.	All those reported to the Enhanced Tuberculosis Surveillance (ETS) system for England during the period 2000–2005.	Details of the ETS system have been published elsewhere.	ETS system	Culture, clinical or radiological signs
Gele 2009	Patients over 15 yrs of age with pastoralist identity in the intensive phase of TB treatment from June to September 2007.	Selected from the TB management units in Jigjiga and Shinile zones of the region. Treatment failures, relapse case and non-pastoralists excluded.	Pre-tested structured questionnaires. Subjects' out-patient cards, patients' registration cards, laboratory registrations and TB registration books	Self-report, out-patient cards, lab registrations and TB registration books	Sputum smear, clinical diagnosis
Hoa 2009	Adult (>=15 years) in the study area	One adult randomly selected from each household	Trained staff with pre-tested questionnaire	Knowledge score and self-report of stigma and source of TB information	NA
Lu 2009	Residents aged between 12 and 65 years, who had lived in their residences for more than 6 months before the 'Third National Health Service Survey (NHSS) in China. Sample size calculation was based on the primary objective of estimating the TB awareness rate (50%). Sample of 70 000 was considered appropriate.	Five-stage sampling scheme was adopted. 60 counties were selected from 19 provinces using stratified random sampling (< 3rd NHSS). 2 towns with gross domestic income levels at approx. the upper and lower quartiles; 3 village communities; 120 households. Finally, two residents from each household by selecting dates of birth closest to the interview date.	Structured questionnaire was developed based on the literature review. Validity of the questionnaire was established through content and expert validity. Participants were interviewed by investigators from local CDCs and TB dispensaries who were trained at the provincial level.	Self-report	NA
Mesfin 2009	Newly diagnosed pulmonary TB patients, ≥15 years of age, from the study districts at the time of diagnosis, and suspected cases referred to the diagnostic centers for diagnosis, from January 12, 2005 to January 12, 2006, in Tigray Region, northern Ethiopia	To improve the validity of our findings, we increased sample size by a factor of 2.4 to allow for drop-out and to permit reliable estimates for gender and other potential risk factors. 10/36 districts were randomly selected. All TB patients and suspected cases were eligible.	Interviewed using a pre-tested questionnaire translated into the local language.	Self-report	Sputum microscopy, X-ray
Ngadaya 2009	Smear positive TB patients aged 15 years and above who were diagnosed within three months prior to the day of interview, between April and October 2007, in four district hospitals plus a random sample of 10% of all health facilities which offer TB services in the Pwani region which is located in the eastern part of Tanzania Mainland	4 districts were randomly selected out of 6 districts in Pwani region. Identified all smear positive patients who had been diagnosed 3 months prior to the day of interview using registers. We also enrolled smear positive patients who have just been diagnosed when the interview was going on.	Structured questionnaire which included open and close-ended questions. A maximum of two weeks was used to collect information in one facility depending on the number of smear positive patients available in the facility as well as patients drugs collecting schedule.	Self-report	Three sputum samples in the form of "spot-morning-spot"
Yimer 2009	Adult (>=15 years) with cough more than 2 weeks, chest pain, and shortness of breath	Cluster sampling of kebeles	Trained staff with pre-tested semi-structured questionnaire	Only heads of the households interviewed to identify cases; self-report of suspects about health-seeking and delay	Sputum smear
Yimer 2009	Adult (>=15 years) with cough more than 2 weeks, chest pain, and shortness of breath	Cluster sampling of kebeles	Trained staff with pre-tested semi-structured questionnaire	Sputum smear, independent reviewer read the smear slides after they were prepared by experienced technicians	Sputum smear
Al-Maniri 2008	All GPs working in both private and public sector in Muscat were targeted in the study. (n = 326; 54.3% private and 45.7% public)	We met 170 (96%) of the private GPs and 117 (78.5%) of the public GPs. 7 of the private GPs were on leave and 32 public GPs were also on leave or away for training courses. 13 (7.6%) refused and 2 returned unanswered questionnaire (1.2%). Among 117 public GPs, 9 (7.8%) refused and 5 (4.3%) did not return the questionnaire.	The questionnaire was reviewed and finalized by specialist physicians. Nurses and health inspectors were trained.	Self-report	Vignettes or written case simulations, TB suspicion and knowledge score

Table S3.Methodological summary of included studies

First Author Year	Inclusion criteria	Selection of study population	Data collection	Measurement of outcome	Assessment of disease
Armijos 2008	Those at least 18 years old, referred to the INHLIP for suggestive symptoms (n = 129) or because of their household contact status (n = 83) were recruited, during a 6-month period (August 1999 to February 2000).	Subjects were enrolled in consecutive order in which they were referred to the INHLIP. No developmental or other conditions that would impede their ability to adequately understand and respond to interview questions. No other household members participating.	Collected during face-to-face interviews with subjects using a structured questionnaire.	Self-report	Sputum smear
Aspler 2008	Patients with confirmed active pulmonary or extra-pulmonary TB who had been on treatment for6–10 weeks and were aged >=18 years	Risk of biased selection on those who could relatively afford treatment	Trained staff and interviewed in local languages	Self-report on direct cost; indirect costs were estimated	TB assessment based on WHO standard
Ayuo 2008	Newly diagnosed smear-positive TB patients who gave consent between January 2002 and December 2004 at study site	Only selected those who came to diagnosis; imbalanced gender distribution (64.3% men vs 35.7% women)	Trained assistant did interview to patients	Self-report of symptom onset	Sputum smear, chest X ray, biopsy of tissues
Bassili 2008	Newly diagnosed, smear-positive,pulmonary tuberculosis cases, 15 years of age and olderin the study settings of the 7 countries	Only selected those who came to diagnosis; samples more likely to represent general condition of countries where nationwide TB survey data is present, less representative in countries lack nationwide survey; Iraq and Yemen could not achieve originally desired sample size because of war	Trained staff with pre-tested structured questionnaire; patients were interviewed in local language and within 2 weeks of treatment initiation	Summation of scores of socioeconomic status, stigma, satisfaction, and knowledge; delay was dichotomized based on median length of different type of delay in each country	Sputum smear
Brassard 2008	Those 18 years of age or older, living primarily in Montreal or another urban area during the previous year, of self-reported First Nation, Me´tis or Inuit descent, and reported no prior history of either a positive TST with appropriate treatment, or treatment for active TB disease.	Recruitment took place during regular activities held at the Native Friendship Centre of Montreal.Outreach workers offering services outside of the NFCM also did recruitment.	Standardized, face-to-face interviews with study participants using a structured interview guide.	Self-report	NA
Chimbanrai 2008	Patients who attended one of the five government hospitals having TB clinics in SamutPrakan Province, during the period November 2005 – May 2006.	Purposive sampling technique was used to recruit 300 TB patients. Patients were followed throughout TB treatment (for 6 months).	Unstructured and structured questionnaires were used as well as medical records and TB cards. Interviews by the researcher or trained researcher assistant.	Knowledge score	Sputum smear
Gosoniu 2008	Around 100 TB patients from each study site were selected.	Only selected those who sought care. Nearly equal balance of men and women.	Trained staff and interviewed with semi-structured questionnaires in local languages	Self report; delay was categorized to problem delay (>90 days), timely diagnosis (<=30 days), or moderate delay (31-90 days)	Assessment of TB was not reported
Kilale 2008	All patients received TB treatment in the selected facilities	Randomly selected health facilities; all patients received TB treatment there were selected	Exit interview	Self report; did not mention how to assess knowledge, except mode of transmission	Assessment of TB was not reported
Lin 2008	New smear-positive TB cases registered in all 129 county TB centers of Yunnan in 2005, China	Of the 11,232 cases of smear-positive pulmonary TB in this study population, 876 (7.8%) were excluded because of the lack of clear information about address, time of onset of symptoms or time of health-seeking behavior.	Web-based Tuberculosis Management Information System. County TB doctors register and update TB patient information every day based on standard operational guidelines. Completeness of the data set was 95% and accuracy was 97.6% (2005). We selected one experienced health officer from each county to collect geographical information.	Computerized medical records, local maps	Sputum smear
Lorent 2008	Newly diagnosed patients with PTB or EPTB, at the Dept of Internal Medicine, Centre HospitalierUniversitaire de Kigali (CHUK), between 1 July and 1 September 2006. Age ⩾15 years, diagnosis of TB and/or start of TB treatment at CHUK	All new eligible patients. 82 consecutive in-patients and 22 outpatients were studied. Six were unable to provide information about the length of their delay prior to transfer and three died before the interview took place.	A questionnaire survey was conducted by interviewing, in the local language by the same medical officer shortly after diagnosis (up to 2 weeks). Case files and referral letters were reviewed to confirm details.	Self-report, case files, referral letters	Recommendations of the national TB guidelines. Majority of the diagnoses of smear - negative PTB and EPTB were based on clinical and radiological criteria.

Table S3.Methodological summary of included studies

First Author Year	Inclusion criteria	Selection of study population	Data collection	Measurement of outcome	Assessment of disease
Maamari 2008	New smear-positive pulmonary TB patients aged more than 15 years old, seen during the period 1 February 2003 to 30 September 2003, in all 13 national TB control program centers implementing DOTS in Syria.	The number enrolled from each center was proportional to the number recorded in that center in 2001. The sample size was based on the estimated incidence of TB.	Interview according to a structured and pre-tested questionnaire. Health workers, including doctors and paramedical staff, were given intensive training on interview and probing techniques. They interviewed the patients during the first 2 weeks of their treatment.	Self-report	Knowledge score, stigma score, satisfaction with care score
Mahendradhata 2008	Registered as smear positive cases in Yogyakarta province, Indonesia, during May 2004 – February 2005, with data on the time between onset of TB symptoms and treatment initiation.	1116 patients were registered as smear positive cases in Yogyakarta province; we give results for 421 (37.7%) about whom data on the time between onset of TB symptoms and treatment initiation had been recorded. (Sex characteristics of study population P = 0.982.)	Limited Access forms, developed by FIDELIS as part of the project monitoring and evaluation system, were introduced into all DOTS services. Health workers of the DOTS services were trained to administer the forms and complete them as soon as smear-positive TB diagnosis was made. Completed forms were collected on a monthly basis by field supervisors and checked for completeness. Second interviews if needed. Brief structured interviews were performed on patients at health facility by health staff.	Self-report, Limited Access forms developed by FIDELIS, electronic database	Sputum smear
Marks 2008	Civilian, non-institutionalized household residents aged ⩾18 years in the USA, taken up in the NHIS data (aggregated 6 years, 2000–2005)	Multistage stratified cluster probability sampling designed to be representative of the US population. Each person in the NHIS sample was selected with a known non-zero probability. Data analysis was conducted on responses weighted for the sampling design, ratio, non-response and post stratification adjustments to census totals for sex.	US National Health Interview Survey 2000-2005, annual face-to-face interviews.	Self-report	NA
Meintjes 2008	TB suspects ≥ 18 years of age admitted to the medical wards at GF Jooste Hospital between February and September 2003. Main inclusion criterion was a TB suspect defined as referral from a primary care facility for diagnostic work-up for TB or in whom the admitting doctor suspected TB.	All eligible TB suspects. Exclusion criteria were treatment for TB within the preceding 6 months, confusion, and if solely on the basis of suspected TB meningitis. Not recruited if admitted over weekends or during the two periods of leave taken by the interviewer. No patients in the study were on ART.	Interviewer-administered semi-structured questionnaire, conducted by a trained interviewer in the patient's home language.	Self-report	Clinical picture, radiological findings, histology/cytology
Mfinanga 2008	Confirmed smear positive acid fast bacilli TB patients who came for treatment and were diagnosed within the past two months, before commencement of the study, in three districts of Dar es Salaam region namely Ilala, Temeke and Mwananyamala, in May 2006	Random selection of hospital, health centers and dispensaries was done. Representativeness of private facilities was ensured. Total of 22 health facilities. 1 private hospital declined to participate and therefore was replaced by a dispensary.	An interviewer administered questionnaire. The 1st part included patient's particulars, knowledge on TB symptoms, transmission, cure, perception on co-infection with HIV and questions on health seeking behavior. 2nd part included record reviews on diagnosis and treatment information extracted from investigation forms, lab, and treatment registers.	Self-report, investigation forms, lab and treatment registers	Smear positive acid fast bacilli patients only, according to the NTLP criteria
Qureshi 2008	Newly diagnosed PTB out-patients (both smear positive as well as smear negative) diagnosed in the last 2 weeks were included coming to the hospitals from May to July 2006, in the TB Outdoor Unit of Chest Department of Nishtar Hospital and Bethania Hospital, Pakistan	Selection? Relapses, re-treatment as well as other forms of TB were excluded. All patients below 15 years were also excluded.	Pre-structured questionnaire	Self-report	Direct microscopy examination
Rumman 2008	Consented household members older than 15 years of age who were not identified as TB cases	Multi-stage stratified sampling to select districts; two different sampling frames for rural and urban regions	Trained staff with pre-tested structured questionnaire	Self-report; stigma score	Sputum smear; clinical examination
Somma 2008	Around 100 TB patients from each study site were selected.	Only selected those who sought care. Nearly equal balance of men and women	Trained staff and interviewed with semi-structured questionnaires in local languages	Patients' self-reported perceived stigma, either voluntary response or response to probing. Stigma was measured by different elements and also as an overall index	N/A

Table S3.Methodological summary of included studies

First Author Year	Inclusion criteria	Selection of study population	Data collection	Measurement of outcome	Assessment of disease
Thierfelder 2008	All adults aged >= 18 years registered with pulmonary TB during 2005–2006.	Stratified sampling was chosen including three regions of Tajikistan, covering 70% of the country's TB centers. A single-stage cluster sampling scheme was used, with TB centers as clusters	Data on hospitalizations were collected from the TB registry books of the National TB control program, available in the TB centers. Data about the centers were collected separately using a questionnaire completed by each center head.	TB registry books, self-report	Sputum, hospitalization defined as referral to the hospital which is documented for each patient in the 2003 version of the WHO TB registry form
Thongraung 2008	New TB patients aged 15 years orolder who had just finished treatment with no history of an allergy to anti-TB drugs	Only selected those who sought care; stratified sampling; referral and MDRTB were excluded	Medical record review to identify responsible staff at each stage of care of cases; interview with care providers	Pre-tested checklist to evaluate adherence; missing information was coded as non-adherence	Based on national TB program policy
Wang, J. M. 2008	Individuals in selected households aged 12-65 whose birthdays were closest to investigation dates (study1); all TB suspects indentified (study2)	Systemic sampling from general population; 90.3% eligible individuals completed interview (study1); stratified cluster sampling to arrive the study population for TB screening (study2)	Trained staff with structured questionnaire; 10% randomly selected to be telephone re-interviewed	Self-report	Chest X ray and sputum smear
Wang, Y. 2008	Adult (>=15 years) patients with a history of a cough, hemoptysis, or sputum with blood for more than 3 weeks in the selected districts	Only selected those who sought care; one quarter of rural-to-urban migrants and the rest permanent residents to reflect the proportion of rural-to-urban migrants;	Trained staff with standard interview; 10% participants were re-interviewed as quality assurance	Self-report	Assessment of TB was not reported
Weiss 2008	Around 100 TB patients from each study site were selected.	Only selected those who sought care. Nearly equal balance of men and women, and representation of newly treated patients and those who had been treated for longer time.	Trained staff and interviewed in local languages	Self-report	Assessment of TB was not reported
Chang 2007	Smear positive pulmonary TB patients aged 15 or older	Only selected those who sought care; one third of districts in the study site were selected; all newly-diagnosed TB patients were included; imbalanced gender distribution	Semi-structured pre-tested questionnaires, trained staff interviewed patients after diagnosis of TB or while admission	Self-report of patient delay; diagnostic delay can be cross-verified by treatment cards; median delays were used to dichotomize outcomes	Sputum smear, with quality assurance procedures
Huong 2007	Newly diagnosed smear-positive pulmonary TB patients in the third quarter of 2002 in 70 districts of Vietnam	Only selected those who sought care; stratified cluster sampling for 70 districts	Trained staff with pre-coded structured interview	Self-report	Sputum smear
Karim 2007	Newly diagnosed smear-positive TB patients who gave consent between January 2002 and December 2004 at study site	Only selected those who sought care; convenient sampling from each of the ten study sites; balanced gender selection and within one month of treatment initiation to prevent selection bias	Trained staff to interview; cross-validated on dates with patients' spouses and local events	Self-report delays in diagnosis and/or treatment initiation of TB	Sputum smear
Karim 2007	TB patients who were receiving treatments (half intensive phase, half continuation phase)	Purposive selection to have balanced gender distribution, however only studied those who came to medical help	Semi-structured EMIC questionnaire	Self-reported stigma, either as individual items or as an overall stigma index	NA
Kaulagekar 2007	Individuals claimed to have TB at a national survey	Weighted stratified random sample from nationwide survey	Questionnaire answered by a responsible adult from each household	Self-report	Self-report
Kemp 2007	New patients in intensive phase of treatment >=16 years	Only selected those who were on intensive treatment then	Trained staff and interviewed in local languages	Self-report; indirect costs for women and household costs were at risk of overestimation	Diagnosis based on WHO guideline
Pehme 2007	All newly diagnosed symptomatic culture positive patients with PTB aged >=16, culture positivity for Mycobacterium tuberculosis and notification in the Estonian Tuberculosis Registry from January 2002 to December 2003.	Tartu, Valga, Võru, Viljandi, Põlva and Jõgeva counties in Southern Estonia (26% of the total population of Estonia). Of the 187 eligible patients, two were lost to follow-up after diagnosis	Patients were interviewed face-to-face within 30 days of diagnosis using an interviewer administered questionnaire, in the language of the patient's preference (Estonian or Russian).	Self-report, medical records	Diagnosis of PTB in Estonia requires both smear/culture Results and CXR
Selvam 2007	New smear-positive patients diagnosed and treated between January and March 2003 in government facilities of randomly selected blocks in Tamil Nadu, India	One TB Unit from each of the 29 districts was randomly selected. Patients were interviewed at health facilities, their homes or their work places, within 1 week of starting treatment. Three attempts were made to contact and interview each patient.	Pre-tested, semi-structured, coded interview schedule after obtaining informed consent, by RNTCP staff specifically trained for this purpose at the State headquarters	Self-report	Sputum smear

Table S3.Methodological summary of included studies

First Author<br>Year	Inclusion criteria	Selection of study population	Data collection	Measurement of outcome	Assessment of disease
Sharma<br>2007	Representative sample of the general population during a period of 8 months from 2 and January to 31 August 2003, in Delhi. Final sample consisted of 1,008 persons.	Multistage stratified systematic sampling was applied. In case there was more than one unrelated adult member residing in the same premises, all were included.	Pre-designed, semi-structured proforma. Six interviewers freshly recruited by the government for the RNTCP program but were not yet involved in its implementation, conducted the interviews. They were given two-days training	Self-report	NA
Tiwari<br>2007	TB patients using DOTS services at Health facilities in Dang District, Nepal, in 2002-2003	Study areas were selected by literature review, review of national and local newspapers, government documents and discussion with local researchers. 200 patients were randomly selected from patient list	Structured face to face interview. Data collected was verified by local level research assistants	Self-report	Sputum smear
Wondimu<br>2007	All pulmonary patients(smear-positive and smear-negative) in intensive phase of treatment >=15 years	Only selected those who sought care, and only selected health facilities with higher TB case detection rates (>5 cases/month)	Trained staff with pre-tested semi-structured questionnaire; local event calendar was used to help recall onset of symptoms	Self report; knowledge level of TB was categorized based on the distribution of patients' performance	Sputum smear; function grades of patients
Xu<br>2007	Patients with more than two weeks of cough who visited the outpatient department of the sampled<br>township and county hospitals in a DOTS county (Jianhu) and a non-DOTS county (Funing) in Jiangsu Province, during 2002-03	One-third of the townshiphospitals were purposively sampled according to the socioeconomic status andfeasibility of data collection. The county hospitals werealso included as sample clusters. No gender difference (P=0.206)	Subjects recruited in the township hospitals were interviewed by the treating physicians. Subjects recruited in the county hospitals were interviewed by the researchers and postgraduate students. A semi-structured questionnaire was designed	Self-report	Sputum smear
Yan<br>2007	TB patients: sputum smear confirmed new or retreatment TB patients. TB suspects: persistent cough for three weeks, or had coughed blood or sputum tinged with blood within the last year	Only selected those who sought care in quantitative study; patients included those newly treated and those had been treated for a longer period. Balanced selection from the rich and poor, and from those with and without accessibility to transportation; participants in qualitative study were selected to represent different stakeholders.	Data collected by local-level staff for quantitative study, whereas national- or provincial-level researcher collected data for qualitative study. All of whom received training.	Self-report	Sputum smear
Zhang<br>2007	Individuals aged 18 to 60 in the study sites	Multi-stage random sampling with focus only on rural areas and likelihood to over-represent Mangolians who settled in villages; did not mention how FDG participants were selected	Trained staff with pre-tested structured questionnaire; FDGs	Self-report	NA
Farah<br>2006	People aged 15 and above were reported to the NTR from the mainOslo and Akershus region hospitals from July 1, 2003 to February 6, 2004	Only selected cases from the main Oslo and Akershus region hospitals from national registration	Review of clinical case notes,referral cases notes, and notification forms of NTR, no interview of cases	Symptom onsets were estimated through data review; only 73.4% participants had fullinformation about delays at different levels	Clinical notes, microscopic results, and registration forms in NTR
Fochsen<br>2006	All persons aged 15 years in the demographic surveillance site with a cough more than 3 weeks	Suspects were identified only by house representatives, of whom 70% were men; 31.6% suspects did not return to receive interviews, and the drop-outs were younger (p=0.01) and more likely to be women (p=0.001)	Trained staff with pre-tested structured questionnaire	Self-report	Sputum smear; chest X rays
Pehme<br>2006	Newly diagnosed symptomatic culture positive patients with PTB aged >=16, culture positivity for Mycobacterium tuberculosis and notification in the Estonian Tuberculosis Registry from January 2002 to December 2003.	Tartu, Valga, Võru, Viljandi, Põlva and Jõgeva counties in Southern Estonia (26% of the total population of Estonia). Of the 187 eligible patients, two were lost to follow-up after diagnosis	Patients were interviewed face-to-face within 30 days of diagnosis using an interviewer administered questionnaire, in the language of the patient's preference (Estonian or Russian). Modified with the authors' permission from a formerly implemented one.	Self-report, medical records	Diagnosis of PTB in Estonia requires both smear/culture<br>Results and CXR
Rojpibulstit<br>2006	All newly diagnosed pulmonary TB (PTB) patients aged at least 15 years who were either sputum smear-positive or smear negative, from June 2003 to April 2004, in Songkhla province, Thailand. Smear negative and finally diagnosed as non-TB, those diagnosed without sputumexamination, and prisoners were excluded.	Five community hospitals and two tertiary hospitals were selected (how?); each had at least 10 new cases per 4 month cohort.	Structured questionnaire, pre-tested with 30 new TB patients and thoroughly revised was used by a well-trained researcher. Interview data were also checked against the data in medical records	Self-report, medical records	Sputum smear

Table S3.Methodological summary of included studies

First Author Year	Inclusion criteria	Selection of study population	Data collection	Measurement of outcome	Assessment of disease
Saly 2006	All new smear positive pulmonary TB patients, 15 years or older, registered in the study sites from May 1st to July 31st 2002, in six rural districts of six different provinces in Cambodia. Those who rejected the interviews or came from outside the district coverage area were excluded.	Two pilot districts with decentralized DOTS and four control districts without decentralized DOTS were selected, based on services provided, TB diagnostic services, and TB prevalence.	Structured questionnaire	Self-report	Sputum smear
Sarmiento 2006	TB patients over 18 years of age within 2 months of their enrollment into DOT from May 2001 to December 2004 in Harlem Hospital Center, NYC	All new TB cases were approached. Over the 3-year period, 39 completed the interview, one refused to participate.	Semi-structured questionnaire, pilot-tested prior to the study to ensure the clarity and precision. Research assistants, who received training, conducted the interviews in English, French & Spanish.	Self-report	NA
van der Werf 2006	All patients >15 years old who were diagnosed with PTB <4 months before the interview (to reduce recall bias), within the Kiev City TB services, between March and November 2004. Patients who did not speak Ukrainian or Russian, those with a chronic underlying pulmonary condition and those who were seriously ill were excluded.	Newly diagnosed PTB patients detected by passive case finding from the four facilities that admit TB patients for treatment. The number of patients selected per facility was proportional to the number of new patients admitted to the facility in 2002	Four interviewers were trained to perform the data collection. They collected information from each patient from the medical record and by face-to-face interview using a structured quantitative questionnaire.	Self-report, medical records	Sputum smear microscopy, culture and chest X-ray
Wandwalo 2006	Study nested to a randomized-controlled study which was conducted in Temeke district, between 1 July and 30 September 2002.	Systematic sampling. For the qualitative study, focus group discussions (FGD) were conducted to TB patients, treatment supporters, health workers and community members. Selection was intended to give balance over sex.	Semi structured questionnaire administered by trained research assistants	Self-report	Sputum examination
Becerra 2005	TB index cases and their household and neighborhood contacts of pulmonary TB patients in El Progreso health center between January 1, 1996 and April 21, 1997	Contacts were selected from the index household and neighboring households in a systemic way; 4 (2%) index households refused to participate	Trained staff interviewed contacts and collected sociodemographic characteristics and duration of cough; information regarding cases were retrieved from records	TB cases by sputum smear	Sputum smear and culture exam
Cheng 2005	New pulmonary smear-positive TB patients, diagnosed after sputum examination using Ziehl-Neelson staining and microscopy, who were registered at TB dispensaries in four counties in Shandong Province, in 2001	Economic status was the main criterion for county selection, plus willingness of local institutions to collaborate and feasibility of transportation to the counties. Townships were randomly selected.	Data were obtained from a structured questionnaire and case records kept in TB dispensaries.	Self-report, case records	Sputum smear
Date 2005	New smear-positive PTB patients from 29 December 2001 to 20 March 2002 at the National Tuberculosis Institute.	Individual interviews and data collection were conducted for 74 smear-positive pulmonary TB patients visiting the National Tuberculosis Institute in Sana'a. All new eligible patients.	Every day during the study period, all patients who visited the consultation room were interviewed using a questionnaire modified from Ishikawa. The interviews were conducted in the Yemeni dialect of Arabic with the help of an Arabic-English interpreter.	Self-report	Sputum smear
Díez 2005	Individuals with positive sputum culture between May 1996 and April 1997	Culture-positive TB patients who were registered in databases in 13 of the 17 autonomous regions of Spain; 16.6% excluded because of missing data	Trained physician retrieved time of diagnosis and initiation of treatment from records in medical facilities only; quality assurance procedure was implemented	Estimate health system delay from times retrieved	Sputum smear, culture
dos Santos 2005	Pulmonary TB patients aged 18 or more who started TB treatment between May 2001 and May 2003 in study site	Only selected those who started treatment; 21 (1.9%) individuals were excluded because of incomplete information about the time of symptom onset	Trained staff and standard questionnaire; patients were not aware of if they had acceptable delay to avoid recall bias	Self report	Sputum smear, culture
Katamba 2005	TB patients on CB-DOT in Kiboga district and those on SAT in Mubende district, 18yrs of age or older, and started treatment between January 2000 and May 2000, Uganda	All eligible patients. Excluded If psychiatric illness, deafness, absence during study period, unable to speak Luganda or English, or previous treatment before Jan 2000 and after May 2000	Structured questionnaire and piloted in a small convenience sample (n=10) both in the English and Luganda version.	Self-report	NA

Table S3.Methodological summary of included studies

First Author Year	Inclusion criteria	Selection of study population	Data collection	Measurement of outcome	Assessment of disease
Kiwuwa 2005	Patients presenting to the center aged 18 years or older, with newly diagnosed sputum smear positive pulmonary tuberculosis, during a four month period from January to May 2002. 1,382 TB patients were registered at the National TB and Leprosy control program center of Mulago hospital, situated in the city Kampala center, Uganda.	TB register at the treatment center was used to identify eligible hospitalized patients. Retreatment patients and those with sputum negative disease were excluded.	Interviewed by one of three trained research assistants including the principal author within two days of the pulmonary tuberculosis diagnosis using a semi-structured questionnaire. Pre-tested among TB patients. Most interviews were conducted in the main local language of the area (Luganda), or in English.	Self-report, case notes, referral letters.	Chest radiographic Findings, sputum smear
Lambert 2005	Every individual who was aged>14 years and who was registered as a new, smear-positive patient in the NTP, in the city of Cochabamba, between March 2001 and March 2002	Only interviewed those come to diagnoses	Data were collected within two months of initiation of treatment to minimize recall bias; use of local event calendar to help recall; patients interviewed in Spanish or local languages	Self-report	Sputum smear
Promtussananon 2005	Community members from different age groups in a semi-urban community in Mankweng, with a population of 11,139 in Limpopo province, South Africa	Selected on the basis of quota sampling by age and sex. 10 males and 10 females were selected from four age bands: 11-19, 20-34, 35-49 and >=50. After randomly assigning the first house, participants were identified by approaching every fourth house from the previously approached one.	Information was gathered from a community population using the individual structured interview method. One trained research assistant conducted interviews in Northern Sotho, the first language in the Mankweng area.	Self-report	Sputum smear
Rozovsky-Weinberger 2005	Patients > 18 years of age with culture proven pulmonary TB treated at three public hospitals and seven not-for-profit private hospitals in Chicago, Los Angeles, and southern Florida that provided care for five or more patients with TB per year during during 1996 to 1999.	Randomly selected public, for-profit, or not-for-profit hospitals in Chicago, Los Angeles, and Miami.	Nurses reviewed inpatient medical records of each patient using a standardized data collection form. Standard risk factors, as defined by the ATS and the CDC, for tuberculous infection and for development of tuberculous disease after infection were sought from the medical record	Medical records	Sputum smear
Xu 2005	Newly diagnosed TB patients, by the County TB Dispensary of Jianhu and Funing County, China, between 1 January and 31 December 2002.	Subjects were interviewed at the time of TB diagnosis at the CTD in JH or in the department of internal medicine of the township or county hospitals and the CTD in FN. No difference in gender (P=0.314)	Structured questionnaire by physicians who underwent a 2-day training course for the interview	Self-report	Sputum smear, CRX films and medical charts based on WHO and IUATLD criteria
Yimer 2005	New smear positive PTB patients above 15 years of age. Smear negatives, relapsed or failed treatment were excluded	6 zones randomly out of the 11 zones of the study region followed by listing all TBMUs in the selected zones, then we took 20 TBMUs randomly as a study sites. We interviewed study subjects consecutively until the intended sample size was achieved	A pre-tested semi-structured questionnaire was administered to collect the intended data.	Self-report	Chest X-ray, sputum results
Ahsan 2004	New TB cases, age ≥15 years, at eleven rural health centers (UZHCs) and one community (Dattapara) from the Dhaka division of Bangladesh. Newly detected TB case was regarded as a sputum smear positive TB-patient who had never been diagnosed or treated for TB or had taken anti-tuberculosis drugs for less than four weeks.	From each health center not more than 14 male and 14 female new cases were selected for equal representations by sex. Since female case detection (TB) is very low in the study area (M: F=10:3), all eligible female cases were selected for the study but male cases were selected by a systematic sampling.	NA	NA	Sputum smear
Atre 2004	Community members, who personally and whose immediate family members did not currently have active TB, were studied with EMIC interviews.	A total of 20 villages, where patients have been registered and treated in the National TB Program were selected randomly for the study. A sample was drawn from these villages using a multistage sampling procedure.	Interviews were developed and locally adapted in pilot testing, and the research interviews were completed between November 1998 and June 1999. Female researchers interviewed women respondents and male researchers interviewed men.	Self-report	NA

Table S3.Methodological summary of included studies

First Author<br>Year	Inclusion criteria	Selection of study population	Data collection	Measurement of outcome	Assessment of disease
Balasubramanian<br>2004	Adults >14 years of age were surveyed from December 1998 to June 2001, in sub-district in Tiruvallur, south India, where the RNTCP was introduced in May 1999.	1) a sample of adults contacted by a house to house community survey; 2) self-referred out-patients seeking medical care at governmental PHIs; 3) TB suspects referred for sputum microscopy; and 4) TB patients treated under the RNTCP at the 17 PHIs.	Community survey, out-patient clinics/PHI's.	Self-report, lab register, patient treatment cards	Sputum smear, X-ray,<br>Standard international definitions were used for disease classification and treatment outcomes
Dandona<br>2004	Patients aged 16 or older and registered for diagnosis and treatment between January and December 2002	Only selected those who came to service; states, districts, and TB units selected to berepresentative; For utilization, register data was used. For barriers to TB service, only 46% of eligible individuals were available to interviews	Use of registered data; trained staff with structured questionnaire; questionnaire was translated into local language and back translated into English; responses were marked by field staff to the most correlated response on questionnaire (subject to misclassification)	Diagnostic and treatment registration; self-report	Assessment of TB was not reported
Díez<br>2004	All TB cases diagnosed between May 1996 and April 1997 in 13 autonomous regions of Spain	Only selected those who sought treatment; individuals with incomplete information were excluded; no statistical difference in demographic variables between eligible and study population	From clinical record	Two cut-off value to dichotomize patient delay based on distribution of patient delays (median and 75th percentile)	Sputum smear or culture
Hamid Salim<br>2004	People aged 12 or more	Large-scale randomized clustered sampling, 15.9% initially surveyed could not be interviewed due to absence; 70% of eligible patients were interviewed	Teams consisted of trained male and female worker did house-to-house interview	self-report cough symptoms for TB suspects; sputum smear for cases	Sputum smear
Hoa<br>2004	New pulmonary TB patients aged 15 or more who had been treated for 1 month or longer	Only selected those who were treated; 86.7% of eligible individuals were interviewed from every district in the study site	Questionnaire was pre-tested and used understandable questions to interviewees.	Cumulative knowledge score; self-report perceived stigma	Sputum smear
Koay<br>2004	Community members aged >= 19 years in Kudat district, Malaysia, from 9 December 1996 to 24 December 1996. Excluded if past history of TB.	Multistage random sampling. 2/5 operation areas were selected using simple random sampling methods. Banggi district was excluded. 2 selected areas were Sikuati and Tinangol. In Tinangol 9/24 villages where 105/435 houses were selected. In Sikuati, 10/30 villages where 105/430 houses were selected ie. every 4th	Questionnaire designed after literature review and preliminary discussions with supervisor. Pre-testing done. Training section organized by the author to brief two public health assistants. 2 public health assistants from the vector-borne disease unit carried out the fieldwork. If head of household was not available, wives, parents or children above 19 years were interviewed in that order.	Self-report	Number of correct responses to the question on TB symptoms was score
Odusanya<br>2004	New pulmonary tuberculosis patients seen between September 2000 and January 2001, at the chest clinic of General Hospital, Ikeja in Lagos, Nigeria	All new PTB patients, either sputum positive or sputum negative for acid-fast bacilli. Sputum positive case show acid-fast bacilli on at least two samples. Smear negative cases are diagnosed on clinical findings, Mantoux test and chest X-ray.	Staff at the chest clinic administered an interview schedule to patients recruited. Patients were interviewed at their first visit to the chest clinic	Self-report	Sputum positive or sputum negative for acid-fast bacilli, clinical findings, Mantoux Test and Chest X-ray
Paynter<br>2004	Patients notified with pulmonary tuberculosis at the North Middlesex University Hospital (UK) chest clinic between 1 April 2001 and 1 March 2002 found by passive case finding methods.	In 71 of the 72 patients eligible, the date of onset of symptoms was estimated. Data on the date of starting treatment were available for 70 of the 71 patients (one patient defaulted before starting treatment).	Hospital notes and notification forms. Data were also collected from GPs and from the NMUH Accident & Emergency Dept if the patient had attended since the onset of TB symptoms. Data from GPs were sought through a postal questionnaire.	Hospital notes, notification forms, postal questionnaire, NMUH electronic database	Sputum smear, CXR
Shetty<br>2004	TB patients of Somalian origin in a TB clinic in London between February and July 2000 and controls either from patients' households or a group of unconnected Somalians whose immediate family did not have experience with TB	Household controls were chosen to be the person who most influenced treatment seeking behavior and treatment decision; unconncected controls were chosen to represent the cross-sectional view of TB-unexperiencedSomalian in central London	A trained Somali doctor used piloted close-ended structured questionnaire to gain information.	Self-report of patients' knowledge of, attitude to, and belief/practice regarding TB. Affective and cognitive responses to TB were coded as scales.	NA

Table S3.Methodological summary of included studies

First Author Year	Inclusion criteria	Selection of study population	Data collection	Measurement of outcome	Assessment of disease
Thorson 2004	Individuals aged 15 or more in the Bavi District, Ha Tay province in north-west Vietnam, during April–June 2000	67/352 clusters were selected by randomized stratified cluster sampling. Potential TB cases were identified by interviewing (female interviewers) the household representative (the female senior head of family).	Structured questionnaire. Medically educated supervisors participated in quality checking of the data collection. One-fourth of all cases were randomly selected for a re-interview by a supervisor.	FilaBavi baseline data collection, records from the NTP, self-report	Sputum samples were read and classified according to WHO guidelines; CXR read by two physicians
Agboatwalla 2003	Any family members 20–45yearswho had stayed more than 6 months in the selected households	200 households (100 urban and 100 rural) were randomly selected from voter registration	Semi-structured questionnaires, interview in national and local languages	Self-report	NA
Ailinger 2003	U.S. residents who responded to the 1994 National Health Interview Survey (NHIS) (National Center for Health Statistics (NCHS), 1997).	Ongoing multistage national probability sample of the U.S. population to survey health. In 1994, 45,705 households containing 116,079 persons in all the states and the District of Columbia were interviewed. The interview response rate was 94.1%.	Interviewers from the U.S. Bureau of the Census. NCHSA core questionnaire was administered to an adult in each household, and the AIDS Knowledge and Attitudes questionnaire, which contained 5 questions on TB knowledge/risk, administered to 19,127 persons within these households. Respondents included one randomly selected adult.	Self-report	NA
Harries 2003	New PTB (smear-positive and smear-negative) in the wards at the time of the visit and identified through the TB register and treatment cards, between April and June 2002, in all 44 non-private hospitals (four central, 22 district and 18 mission) in Malawi. Patients had to be well enough to be interviewed, and had to have had a cough which prompted investigations for PTB.	44 non-private hospitals (four central, 22 district and 18 mission) that currently register and treat patients with TB. All the above hospitals were visited. To ensure standardization in sample selection, interviews were always started with the patient in the first bed on each ward until either all patients were interviewed or the maximum number of interviews was achieved.	Patients underwent a structured interview by one of the authors, usually in the local language, accompanied by the hospital TB officer and/or TB ward nurse.	TB register and treatment cards, self-report	Sputum smear
Hashim 2003	TB patients and health care workers at Primary Health Centers in Iraq, during a 1-year period (November 2001 to November 2002). Patients younger than 15 years old and those with mental disability or with speech or hearing problems were excluded.	Random sample. 2 sampling frames of urban and rural PHCs were developed, with the number of selected urban or rural PHCs proportionate to the total number of PHCs in that frame. PHCs selected using a systematic sampling technique. Total of 250 were finally selected out of 975 PHCs (404 rural 541 urban) distributed over all Iraqi governorates.	Questionnaire forms were designed, 1 for health care workers and 1 for TB patients, filled by face-to-face interview. Reliability was tested on 30 patients and 30 health care workers who were interviewed twice by the same interviewer (for inter-rater variation) and by another interviewer (for intra-rater variation).	Self-report	Every question was rated and a total score was obtained for patients' and health care workers' knowledge, attitudes or practices.
Hoa 2003	Individuals aged 15 or older had a cough more than 3 weeks at the time of interview in the study site	Random clustered sampling of surveillance site	Trained staff with structured interview; pre-tested questionnaire; a 15% random sample were selected and re-interviewed	Knowledge score	Self-report
Kamel 2003	Group1: 260 newly diagnosed TB cases who commenced anti-TB treatment through the DOTS program during December 2001 to November 2002, all were registered in 7 chest clinics in Alexandria. Group2: 74 patients who had been started treatment within 3 months prior to the study.	At risk of selection bias because of imbalanced gender distribution (69.2% men vs 30.8% women).	Interview by trained staff plus data retrieved from treatment record or control card	Self-report for health care utilization; WHO definitions for TB treatment outcomes	Sputum smear
Santha 2003	All TB patients detected at health facilities during the first year of DOTS implementation (May 1999–April 2000), and all patients identified by community survey during the same period.	For the community survey, planned to be completed over a 3-year period, 82 000 adults were selected from approximately 25 000 households using a multistage cluster sampling technique.	Community survey for active tuberculosis detection was undertaken in the study area as part of a larger epidemiological investigation	Treatment cards, self-report	Sputum smears for acid-fast bacilli, chest X-ray. A diagnosis of tuberculosis is made using RNTCP criteria.

Table S3.Methodological summary of included studies

First Author<br>Year	Inclusion criteria	Selection of study population	Data collection	Measurement of outcome	Assessment of disease
Sudha<br>2003	Chest symptomatics, defined as persons with symptoms of cough for 3 weeks or more, with or without chest pain, fever, loss of weight or hemoptysis during the 3 months preceding the interview,in two urban and two rural communities in Tamil Nadu State, South India, between May and October 1997.	Probability proportional to size method was used to select 30 divisions/30 wards in each urban and rural communities. Next, a street was selected randomly; finally, and house was chosen randomly. No difference in sex.	Trained and experienced medical social workers interviewed each participant with the aid of a structured, pre-coded and pre-tested questionnaire.	Self-report	NA
Demissie<br>2002	New pulmonary tuberculosis patients who were diagnosed, during August 1 to December 31, 1998.	All the 17 public health centers in Addis Ababa are involved in the diagnosis and treatment of TB patients. The health centers are the first health care contacts in Addis Ababa and majority of TB patients are diagnosed and treated in these centers.	Interviewed using a structured questionnaire. The questionnaire used was pre-tested before use and regular supervision of the interviewers was conducted throughout the study period.	Self-report	Sputum smear, X-ray
Dhingra<br>2002	Patients registered under RNTCP for DOTS treatment from March 2001 to August 2001.	301 consecutive patients of PTB registered at RNTCP all the four sub-centers of the New Delhi TB Center.	Pre-tested semi-structured questionnaire by a trained investigator.	Self-report	Diagnosis per guidelines of WHO and IUATLD
Godfrey-Faussett<br>2002	All adult patients (>=15 years)presenting to the urban health centers with a cough that had not previously been assessed in the network ofurban health centers in Lusaka	Only selected those who sought health care; convenient sample of eligible population; imbalanced gender distribution (59.5% women vs 40.5% men)	Interviewed by trained staff in local languages; had addressed on improving patient recall	Self-report	NA
Malhotra<br>2002	Household heads of the study site	Systemic sampling one third of all household heads	Pre-tested structured interview	Self-report	NA
Masjedi<br>2002	Confirmed cases of PTB admitted for the first time to the National Research Institute for Tuberculosis and Lung Disease, between August 1995 and February 1996, in Teheran. Diagnosed for the first time; could remember his/her disease course accurately; diagnosis of pulmonary TB was confirmed by a positive sputum smear; history given by the patient could be correlated with the date of prescriptions and laboratory test results.	All newly confirmed cases admitted for the first time were included.	Patients were all questioned by a single physician.	Self-report	Smear test
Rajeswari<br>2002	New smear-positive pulmonary tuberculosis patients aged >=15 years diagnosed during the preceding month and subsequently undergoing treatment, over a 20-month period, from April 1997 through November 1998. Those diagnosed only with X-ray and previous history of treatment wereexcluded.	Four adjacent districts to Chennai city were selected for convenience. In all, more than 95% of the eligible patients were interviewed. All new patients were eligible.	After pre-testing on 30 new smear-positive TB patients, a structured questionnaire was used. The interviews were conducted at health facilities. Patients who could not be interviewed at health facilities were visited at home to obtain the required information.	Self-report	CXR, sputum smear
Getahun<br>2001	Heads of household in Amhara Region, North Western Ethiopia, April 1997.	2 of the 'Woredas' were randomly selected by a lottery system and included. 5 rural kebeles were randomly selected by a lottery system and systematic sampling was used to enroll 100 households from each.	20 high school graduates trained for data collection. An open and close-ended pre-tested questionnaire in Amharic was used. Investigators and supervisors regularly monitored.	Self-report	NA
Lienhardt<br>2001	Newly diagnosed TB patients aged over 15 years presenting to TB control staff in four health centers in from January to April 1997	Only selected those who sought health care; 89% of eligible patients participated the study; imbalanced gender distribution (72.4% men vs 27.6% women)	Trained staff with structured interview within 2 weeks of diagnosis; FDGs prior to interview recognized local terms related to TB symptoms and signs; local event calendar was used to help recall	Self-report	Sputum smear
Needham<br>2001	Newly diagnosed adult pulmonary TB patients in the centralized chest clinic over 9 weeks in study site	Only selected those who sought health care; 89.8% eligible patients participated	In-depth interview; did not mention which type of interview was used	Self-report	Sputum smear and culture

Table S3.Methodological summary of included studies

First Author Year	Inclusion criteria	Selection of study population	Data collection	Measurement of outcome	Assessment of disease
Ngamvithayapong 2001	New cases of smear-positive pulmonary TB between April 1998 and May 2000 presenting to Chiang Rai Regional Hospital, Thailand, age > 15, and able to communicate in Thai or northern dialect.	All new cases. 124 cases were excluded because the interview could not be done, including prisoners, unconscious or seriously ill, patients who died at the hospital, hill tribe patients who could not speak Thai or northern dialect, or inpatients who left the hospital before results. 12 could not be located.	One research assistant was posted in the laboratory section to conduct interviews with the patients using a structured questionnaire. Interviews were carried out immediately when the results of sputum smears were known to be positive.	Self-report	X-ray and sputum examination
Pronyk 2001	Hospitalized patients diagnosed with PTB between mid-August and mid-December 1999, Bushbuckridge Region of South Africa's Northern Province. Both new and re-treatment patients, as well as those with sputum negative disease, were included. Patients aged 10 years old or less wereexcluded.	TB registers at the three regional hospitals were regularly searched. There were nine refusals, and several patients could not be interviewed for other reasons.	Semi-structured questionnaire was extensively piloted. The questionnaire was administered by a medical anthropologist and one research assistant; it was conducted in the patients' native language (Shangaan, Swati or Sepulana).	Self-report	Sputum
Sadiq 2001	Newly registered pulmonary tuberculosis patients (less than one week) at TB Center Rawalpindi from 20 November to 21 December 1998, in Pakistan. Sputum smear positive and/or sputum smear negative but X-ray suggestive of pulmonary TB.	All new patients. Exclusion criteria were age less than 13 years, relapses (recurrence after treatment) and re-treatment after previous default of TB Center's patients.	Standardized open-ended questionnaire. Interviewers were trained and a pretest was done for three days to assess the reliability of interviewers. The pretest data was not included.	Self-report	Sputum smear, CXR
Ward 2001	Symptomatic patients from 1985 to 1998 diagnosed on the basis of positive bacteriology and/or histology were included. Visitors with an interval of 2 weeks between arrival and first consultation, relapsed cases and persons whose diagnosis was made post mortem with no record of prior medical consultation, and cases diagnosed via screening were excluded	Queensland TB database contains information on all cases of TB notified in Queensland from 1985.<br>Since the early 1980s the QTCC has been sending standardized post notification questionnaire and follow-up forms to treating physicians	Data extracted from the Queensland TB Control Center database and review of charts.	Database	Bacteriology and/or histology, CXR
Yamasaki-Nakagawa 2001	New cases of TBwho began to receive DOTS in the study area from mid-December 1997 to mid-June 1999	Only selected those who sought health care; 27 men and 27 women were not interviewed, mainly due to death or default, and proportion of interviewed differed significantly across gender (89.8% of men vs 78.4% of women, p=0.002)	Interviewed by trained staff in local languages plus data retrieval from registration documents	self-report	Sputum smear
Thorson 2000	Population-based survey in Ha Tay Province. Participants were aged 15 or over.	Did not provide details of how the selection was done in the survey process.	Structured questionnaire; interview at household	Number of healthcare actions taken, type of actions, delay between symptom onset and first visit of a hospital, cost per visit	Cough > 3 weeks as TB suspect
Wandwalo 2000	New consecutive smear-positive pulmonary tuberculosis patients in Mwanza region, Tanzania, from May to July 1998. Relapses and retreatment cases, as well as children and people below the age of 15 years, were excluded.	Two different populations,, an urban population in Mwanza municipality, and a rural population from three neighboring districts. No randomization.	Interviewed by one of the authors or a trained assistant, using a structured questionnaire. Patient files and TB treatment cards were also checked. Factors responsible for delay and the health facility first visited by the patients were recorded.	Self-report, patient files, treatment cards	Sputum microscopy
Wandwalo 2000	Consecutive smear-positive pulmonary tuberculosis patients in Mwanza region, Tanzania, during a 2month period, from May to July 1998. Relapses and retreatment cases, as well as children and people below the age of 15 years, were excluded.	All new eligible patients. Four patients were excluded from analysis because of insufficient information.	Interviewed by one of the authors or a trained assistant, using a structured questionnaire. Patient files and TB treatment cards were also checked.	Self-report, patient files, treatment cards	Knowledge score, sputum smear
Long 1999	Patients aged 15–49 years withnew smear-positive pulmonary TB detected in the selected districts during 1996	Only selected those who sought health care	Trained staff and interviewed in local languages	Self-report	Sputum smear

Table S3.Methodological summary of included studies

First Author Year	Inclusion criteria	Selection of study population	Data collection	Measurement of outcome	Assessment of disease
Rajeswari 1999	Newly-detected sputum-positivepulmonary tuberculosis patients receiving short-course chemotherapy were enrolled in the study	Rural and urban institutions were selected to be able to obtain a representative sample. Patient selection was random at patient's availability, however this was subject to selection bias already.	Semi-structured pre-coded interview;	Income were calculated from patient's report and validated by prevailing rates in the community. However indirect cost was not calculated for unemployed women because of difficulty to assess, which may lead to bias. 71% of women were not income-earning whereas only 28% of men were not income-earning.	Sputum smear
Sherman 1999	All TB patients in NYC who had a specimen that grew Mycobacterium tuberculosis in April 1994 and without a history of treatment for >=2 days. Patients who first presented to a non-NYC medical provider or who were first diagnosed outside NYC were excluded	TB patients in NYC who had a specimen that grew Mycobacterium tb had been previously identified for a one-month drug resistance prevalence survey (selection?)	Retrospective medical record review by New York City Department of Health (NYC DOH) Bureau of Tuberculosis Control and interview either face-to-face or via telephone	Self-report, medical records	Smear test, CXR
Tulsky 1999	All residents of five homeless shelter in San Francisco on nights during one month in 1993	62.8 % eligible individuals agreed to be interviewed; most common reason for refusal of interview was inadequate financial incentive	Trained staff with pre-tested questionnaire	Proportions to corrected answers and to each categorical responses	NA
Asch 1998	Cases of confirmed active TB, reported between April and September 1993, as they were reported to the Los Angeles County TB control authority by care providers, governmental agencies, and laboratories, as required by law.	Consecutive cases. Children, prisoners, deceased patients, and those who could not speak one of five common languages were excluded.	Trained bilingual interviewers contacted all eligible patients, using the phone numbers provided by the county TB registry, clinics, directory assistance, or friends or relatives listed in medical records.	Self-report, county TB registry, clinics, directory assistance, medical records	Cultures, AFB smears, and X-ray
Lawn 1998	Newly diagnosed smear-positive pulmonary TB patients in study site in 1995	Only selected those who sought care; 70 out-patients and 30 in-patients	Same medical officers interviewed patients shortly after diagnosis in local languages; case-notes were used to cross-validate	Self-report	Sputum smear; chest X rays
Marinac 1998	Non-Caucasian individual over 18 years of age visiting, employed by, or receiving medical care at Truman Medical Center-West, Children's Mercy Hospital, or Samuel Rogers Health Center of Kansas City, Missouri, between July 1994 and January 1995.	Those with employment or training as a health care provider, unwilling to participate in the survey and unable to speak or understand English (unless a translator was available), were excluded. Primarily African Americans born in the USA, with about twice as many women as men.	Interviewed by one of three investigators. The encounter was face-to-face. A $5 honorarium was provided as compensation. The 6-page survey was constructed using the National Health Interview Survey 1992 (DHHS Pub. No. PHS 94-1617) as a model.	Self-report	Knowledge score
Enkhbat 1997	Newly registered patients > 15 yrs old at the 9 specialized TB facilities in Ulaanbaatar, Mongolia, from May 1995 to March 1996	All newly registered patients. Extra-pulmonary TB, with negative findings or no bacteriological examinations and those with unknown dates of initial symptoms excluded.	Skilled medical staff of the Center for Tuberculosis of Mongolia visited the facilities where eligible patients were registered and interviewed them within days after commencement of treatment.	Self-report	Sputum smear, X-ray
Hooi 1994	Patients with pulmonary tuberculosis treated by the Chest Clinic, Penang Hospital	Consecutive patients with pulmonary tuberculosis treated by the Chest Clinic, Penang Hospital; only studied those who presented.	Patient interview and tracking patient's old medical records	Self-report delays with the help of old medical record at different time points along the pathway of healthcare seeking	NA
Mori 1992	Newly detected tuberculosis patients over several months, starting from April 1980, Korea, until the target numbers for each health center and each bacteriological type were reached.	7 health centers, 3 urban and 4 rural, were selected for the study areas (how?). All newly registered patients were eligible. The number of bacteriologically confirmed cases had to be equal to that of non-confirmed ones for each health center, and the total numbers of patients in urban and rural areas equal.	Interview was performed soon after the patient's registration by the tuberculosis workers in charge of the case, as a part of their initial motivation for regular treatment.	Self-report	Knowledge score
Westaway 1989	Black adult clinic attenders aged between 15 and 68 years over a two-week period in April 1988	Not randomly selected but consisted of available respondents who were attending the Primary Health Care Clinic or the Sexually Transmitted Diseases Clinic.	11 black medical/paramedical staff were trained in the use of the structured questionnaire which was written in English. They were asked to translate the items where necessary	Self-report	Knowledge score

Table S3.Methodological summary of included studies

First Author Year	Inclusion criteria	Selection of study population	Data collection	Measurement of outcome	Assessment of disease
Jenkins 1977	Registered ("status") native Indians throughout British Columbia, during 4 months of the summer of 1976	Native communities visited selected by accessibility, availability of some local native "contact" person, and approval of local band council. Native contact was major source of bias. The majority of people interviewed were selected by these contacts as being representatives of their respective communities.	Questionnaire was presented orally, a modified version of one developed by K. Jang, MA. M.sc. for a similar study. Usually accompanied to the homes by a band member, often a social service worker of some kind. If language was a difficulty, the questionnaire was given by the local native community health worker.	Self-report	Knowledge score
CASE-CONTROL STUDY					
Crampin 2004	All confirmed and probable tuberculosis cases who had not had previous tuberculosis, who were normally resident in the district, who were aged at least 15 years, and who were diagnosed between November 1996 and September 2001; controls needed to be without TB infection history	Controls were randomly selected in the community to have same sex, age, and area distribution as to the cases	Questionnaire was only introduced midway in the enrollment period; cases might have died or defaulted	Self-report	Sputum smear, culture, and tissue biopsy of lymph nodes for TB; ElISA and particle agglutination for HIV test
COHORT STUDY					
Jiménez-Corona 2006	TB cases and suspects (cough longer than 15 days)	Passive case finding and active screening for suspects.	Trained staff and interviewed with standardized questionnaire	Self-report for delay;WHO definition of treatment outcomes.	Sputum smear, culture, and genotypic analysis of TB isolates
Rajeswari 2005	Patients registered for treatment between July and December 2000 who completed continuation phase of short course chemotherapy	At risk of selection bias because of attrition during follow-up (32% unavailable at second interview)	Trained field staff with semi-structured interview and pre-coded questionnaire, which was modified from widely-used SF36 questionnaire	Self-report	TB assessment based on Indian RNTCP guideline
Bashour 2003	New smear-positive TB patients seen at all provincial TB centers (14 centers) in the Syrian Arab Republic during the period January 2002 to July 2002	Only 552 of 800 eligible patients were interviewed, and reasons of refusal included poor transportation. Gender distribution was imbalanced, however, there was no difference in female-to-male ratio between study sample (1:1.97) and registered patients (1:1.87)	Trained staff to interview; previously tested questionnaire; patients' treatment card review; younger population tended to have missing information on TB knowledge and perception	Self-report for attitude; WHO definitions for treatment compliance and outcomes	Sputum smear
Box 2003	Self-reported history of HIVinfection and lack of private insurance from July 1994 to February 1997	90% eligible individuals were included	Hospital billing record and hospital information system; systemic queries of reports from non-DUMC care providers	Time and frequency of hospital visit/admission; provision of screening/prevention/antiretroviral therapy/advance directive	Disease classification based on 1993 CDC criteria
Long 2002	Newly smear-positive patients aged 15 to 49 who were diagnosed in 1996	Only selected those who sought health care; stratified sampling for study districts and selected all eligible TB cases	Trained staff with structured interview; patients were interviewed just after the confirmation of diagnosis	Self-report	Sputum smear
Creek 2000	TB patients with at least one sputum smear-positive for AFB, submitted by an out-patient setting, was identified in the Gaborone TB laboratory register for the period 6 January 1997 to 20 May 1997.	All new TB patients. Some were excluded because they were not found in the TB register, missing treatment initiation date, or because their positive sputum exam appeared to have been for routine follow-up rather than diagnostic purposes.	Electronic TB Register is a district-based computerized surveillance system for all registered TB cases in the country, and is used to generate notification reports and cohort analyses. TB surveillance data, obtained by district staff from clinic treatment registers in 24 Botswana health districts, is entered into a PC in each district regularly.	TB laboratory sputum register, annual notification reports, medical records, surveillance data	Sputum smear
Liam 1999	Adult patients commenced on anti-tuberculosis chemotherapy from September 1994 to February 1996	Only selected those who sought diagnosis	Interviewed by one of the authors with a standardized questionnaire; compliance and treatment outcome were retrospectively reviewed from records	Knowledge score; compliance assessed by a dichotomous criteria; treatment outcome determined by WHO definition	Assessment of TB was not reported

Table S3.Methodological summary of included studies

First Author<br>Year	Inclusion criteria	Selection of study population	Data collection	Measurement of outcome	Assessment of disease
Cassel<br>1982	All individuals in selected districts during time of study	Active and passive case finding	Sociodemographic characteristics collected on case identification	TB case detection; patient compliance and TB treatment outcomes; WHO definition to determine treatment outcomes	Sputum smear
RANDOMIZED TRIAL					
Daitiko<br>2009	All individuals in selected districts during time of study	All kebeles at study districts were randomly assigned into intervention or control group; assignment was not blinded to general health worker or TB program manager	Information retrieved from TB registers	Sputum smear (laboratory technician had no knowledge whether sputum samples were from intervention or control kebeles; TB treatment outcome determined by definition used in NTLCP	Sputum smear
Khan<br>2007	Patients were screened by doctors, and those meeting National Tuberculosis Programme (NTP) criteria (history of cough for longer than 3 weeks or fever for 1 month, or both, blood in sputum, night sweating, weight loss and loss of appetite, and age between 14 and 75 years)	All eligible participants were randomly assigned into intervention or control group; assignment was not blinded to patient	From patient's sputum submission	the eff ect of sputum-submission guidance on female patients testing smear positive (primary outcome), male patients testing smear positive, smearpositive sputum specimens submitted by men and women, and inadequate sputum specimens (ie, saliva specimens) submitted by men and women (secondary outcomes)	Sputum smear
