# Supplementary material for: Barriers and Delays in Tuberculosis Diagnosis and Treatment Services: Does Gender Matter?
Source: Tuberc Res Treat. 2014 Apr 28;2014:461935. doi: 10.1155/2014/461935 (PMC4020203; doi:10.1155/2014/461935)
Supplement: Supplementary file 1 — Supplementary Material contains six data tables, which provide detailed summaries of various aspects of the included studies. Tables S1, S2 and S3 present individual study characteristics, the results of the quality assessment and individual study methodologies, respectively. Tables S4, S5, and S6 summarize WHO regional distribution, study population and degree of urbanization, respectively, by outcome type. [file 461935.f1.zip › Table S4.pdf]

Table S4. Summary of WHO regional distribution by outcome type

Outcome Type	Gender difference n (%)		No gender difference n (%)
	**Women > Men**	**Men >Women**	
Individual-level barriers			
Financial	AFRO – 5 (45%); EMRO – 2 (18%); SEARO – 3 (27%); WPRO – 1 (9%)	AFRO – 2 (40%); SEARO – 2 (40%); Multiple – 1 (20%)	AFRO – 2 (33%); EMRO – 1 (16%); SEARO – 2 (33%); WPRO – 1 (16%)
Physical	AFRO – 1 (100%)		AFRO – 1 (13%); AMRO – 1 (13%); EMRO – 2 (25%); SEARO – 3 (38%); WPRO – 1 (13%)
Stigma	AFRO – 2 (18%); AMRO – 1 (9%); EURO – 1 (9%); EMRO – 1 (9%); SEARO – 5 (45%); WPRO – 1 (9%)	AFRO – 1 (50%); AMRO – 1 (50%)	AFRO – 1 (8%); EURO – 1 (8%); EMRO – 2 (16%); SEARO – 4 (33%); WPRO – 3 (25%); Multiple – 1 (8%)
Health Literacy	AFRO – 2 (12%); AMRO – 1 (6%); EMRO –4 (24%); SEARO – 4 (24%); WPRO – 6 (35%)	AFRO – 1 (13%); AMRO – 2 (25%); EURO – 1 (13%); SEARO – 3 (38%); Multiple – 1 (13%)	AFRO – 9 (38%); AMRO – 4 (17%); EMRO – 4 (17%); SEARO – 4 (17%); WPRO – 3 (13%)
Socio-demographic	WPRO – 1 (25%); SEARO – 3 (75%)		AFRO – 1 (50%); WPRO – 1 (50%)
Provider/system- level barriers	AFRO – 2 (25%); AMRO – 1 (13%); EURO – 1 (13%); EMRO – 1 (13%); SEARO – 2 (25%); WPRO – 1 (13%)		AFRO – 3 (27%); AMRO – 1 (9%); EMRO – 3 (27%); SEARO – 3 (27%); WPRO – 1 (9%)
Combined individual, provider, and system-level barriers	AFRO – 2 (40%); AMRO – 1 (20%); SEARO – 1 (20%); WPRO – 1 (20%)	SEARO – 1 (100%)	SEARO – 1 (100%)
Individual-level delay	AFRO – 3 (23%); EURO – 1 (8%); EMRO – 2 (15%); SEARO – 3 (23%); WPRO – 4 (31%)	AFRO – 1 (14%); AMRO – 1 (14%); EURO – 1 (14%); EMRO – 1 (14%); SEARO – 1 (14%); WPRO – 2 (29%)	AFRO – 14 (37%); AMRO – 1 (3%); EURO – 3 (8%); EMRO – 3 (8%); SEARO – 8 (21%); WPRO – 9 (24%);
Provider/system-level delay	AFRO – 3 (27%); AMRO – 1 (9%); EURO – 1 (9%); EMRO – 1 (9%); SEARO – 1 (9%); WPRO – 4 (36%)	AFRO – 1 (50%); EMRO – 1 (50%)	AFRO – 5 (21%); AMRO – 2 (8%); EURO – 3 (13%); SEARO – 7 (29%); WPRO – 7 (29%)
Combined individual, provider, and system-level delay	AFRO – 1 (11%); AMRO – 1 (11%); EURO – 1 (11%); EMRO – 1 (11%); Multiple – 1 (11%); SEARO – 2 (22%); WPRO – 2 (22%)	EMRO – 1 (100%)	AFRO – 5 (29%); AMRO – 3 (18%); EURO – 2 (12%); EMRO – 3 (18%); SEARO – 1 (6%); WPRO – 3 (18%)

WHO region: AFRO, African region; AMRO, region of the Americas; EMRO, Eastern Mediterranean region; EURO, European region; SEARO, South East Asia region; WPRO, Western Pacific region.
