# Supplementary material for: Barriers and Delays in Tuberculosis Diagnosis and Treatment Services: Does Gender Matter?
Source: Tuberc Res Treat. 2014 Apr 28;2014:461935. doi: 10.1155/2014/461935 (PMC4020203; doi:10.1155/2014/461935)
Supplement: Supplementary file 1 — Supplementary Material contains six data tables, which provide detailed summaries of various aspects of the included studies. Tables S1, S2 and S3 present individual study characteristics, the results of the quality assessment and individual study methodologies, respectively. Tables S4, S5, and S6 summarize WHO regional distribution, study population and degree of urbanization, respectively, by outcome type. [file 461935.f1.zip › Table S5.pdf]

Table S5. Summary of study population by outcome type

Outcome Type	Gender difference n (%)		No gender difference n (%)
	**Women > Men**	**Men >Women**	
Individual-level barriers			
Financial	C – 10 (91%); S – 1 (9%)	C-3 (60%); G – 2 (40%)	C – 3 (50%); S – 1 (17%); G – 1 (17%); M – 1 (17%)
Physical	C – 1 (100%)		C -6 (75%); G – 2 (25%)
Stigma	C – 7 (64%); G – 4 (36%)	S – 1 (50%); G – 1 (50%)	C – 5 (42%); G – 6 (50%); M – 1 (8%)
Health Literacy	C – 4 (24%); S – 2 (12%); G – 10 (59%); M – 1 (6%)	C – 4 (50%); G – 3 (38%); M – 1 (12%)	C – 13 (54%); S – 1 (4%); G – 8 (33%); M – 2 (8%)
Socio-demographic	C – 4 (100%)		C – 2 (100)
Provider/system- level barriers	C – 7 (88%); G – 1 (12%)		C – 7 (64%); G – 2 (18%); M – 2 (18%)
Combined individual, provider, and system-level barriers	C – 1 (20%); G – 1 (20%); M – 3 (60%)	C – 1 (100%)	C – 1 (100%)
Individual-level delay	C – 10 (77%); S – 2 (15%) ; M – 1 (8%)	C – 7 (86%); S – 1 (14%)	C – 33 (87%); S – 3 (8%); M – 2 (5%)
Provider/system-level delay	C – 10 (91%); M – 1 (9%)	C – 2 (100%)	C – 24 (100%)
Combined individual, provider, and system-level delay	C – 9 (100%)	C – 1 (100%)	C – 16 (94%); M – 1 (6%)

Study population: C – Persons with diagnosed TB; S –Persons with suspected TB; G – General population; M – Multiple populations
